# Targeting macrophage ferritin heavy chain mitigates ferroptosis and lung injury in experimental acute respiratory distress syndrome

**DOI:** 10.1038/s41467-026-74828-w

**Published:** 2026-06-27

**Authors:** William Z. Zhang, Kihwan Kim, Divya Bhatia, Lynne Faherty, Will Simmons, Eleni Kallinos, Sebastian E. Carrasco, Katherine L. Hoffman, Sean Houghton, Chia-Lang Hsu, Leora Haber, Cem Meydan, Christopher E. Mason, Ananda S. Mirchandani, Sarah R. Walmsley, Parag Goyal, Kuei-Pin Chung, Karla V. Ballman, David Redmond, Joseph D. Mancias, Augustine M. K. Choi, Edward J. Schenck, Maria Plataki, Suzanne M. Cloonan

**Affiliations:** 1 Joan and Sanford I. Weill Department of Medicine, Division of Pulmonary and Critical Care Medicine, Weill Cornell Medicine, New York, NY USA; 2 Joan and Sanford I. Weill Department of Medicine, Division of Nephrology and Hypertension, Weill Cornell Medicine, New York, NY USA; 3 School of Medicine, Trinity Biomedical Sciences Institute, Trinity College Dublin, Dublin, Ireland; 4 Department of Population Health Sciences, Division of Biostatistics, Weill Cornell Medicine, New York, NY USA; 5 Laboratory of Comparative Pathology, Weill Cornell Medicine, Memorial Sloan-Kettering Cancer Center, and Rockefeller University, New York, NY USA; 6 Division of Regenerative Medicine, Ansary Stem Cell Institute, Weill Cornell Medicine, New York, NY USA; 7 Department of Medical Research, National Taiwan University Hospital, Taipei, Taiwan; 8 Weill Cornell Medical College, Weill Cornell Medicine, New York, NY USA; 9 Department of Physiology and Biophysics, Weill Cornell Medicine, New York, NY USA; 10 The HRH Prince Alwaleed Bin Talal Bin Abdulaziz Alsaud Institute for Computational Biomedicine, Weill Cornell Medicine, New York, NY USA; 11 University of Edinburgh Centre for Inflammation Research, Institute for Regeneration and Repair, University of Edinburgh, Edinburgh, UK; 12 Joan and Sanford I. Weill Department of Medicine, Division of Cardiology, Weill Cornell Medicine, New York, NY USA; 13 Department of Laboratory Medicine, College of Medicine, National Taiwan University, Taipei, Taiwan; 14 Department of Laboratory Medicine, National Taiwan University Hospital, Taipei, Taiwan; 15 Department of Radiation Oncology, Dana-Farber Cancer Institute, Boston, MA USA; 16 Department of Academic Medicine, Tallaght University Hospital and Trinity College Dublin, Dublin, Ireland

**Keywords:** Cell death, Respiratory distress syndrome, Iron, Cell death and immune response

## Abstract

Ferritin, composed of heavy chain (FTH1) and light chain (FTL) subunits, is a key intracellular iron storage protein, but the origin and biological role of extracellular ferritin (ex-ferritin) remain poorly understood. Elevated serum ex-ferritin is associated with worse outcomes in acute respiratory distress syndrome (ARDS). Here, we show that both FTH1 and FTL are significantly enriched in the serum, blood monocytes, and alveolar macrophages (AM) of individuals with ARDS, findings we replicate in a murine hyperoxia-induced acute lung injury model. Myeloid-specific FTH1 (*Fth1*^*ΔLysM*^) deletion attenuates lung injury, and is associated with reduced macrophage ferroptosis, altered airway inflammatory responses, lower extracellular iron and compensatory secretion of FTL-ex-ferritin. While pharmacologic ferroptosis inhibition prior to hyperoxia had no effect, transplantation of FTL-ex-ferritin-enriched bronchoalveolar lavage fluid conferred protection from lung injury. These findings identify macrophage ferritin metabolism and ex-ferritin secretion as critical regulators of lung injury, offering new insights into the pathobiology of ARDS.

## Abstract (web summary)

This study shows that the iron-storage protein ferritin shapes lung injury. Altering macrophage ferritin handling reduces iron-driven cell death and inflammation, improves survival, and reveals ferritin as a regulator, not just a marker, of acute respiratory distress syndrome.

## Introduction

Acute respiratory distress syndrome (ARDS) is a heterogeneous syndrome that is characterized clinically by rapid onset of respiratory failure and histologically by acute lung injury (ALI) and diffuse alveolar damage^1,2^. While both infectious and noninfectious etiologies can precipitate ARDS development, ARDS, at its core, is characterized by abnormal inflammation and alveolar barrier disruption that precipitates an influx of protein- and immune cell-rich edema fluid^1^, impeding gas exchange. Among the immune cells that play a central role in ARDS are lung macrophages^3^, a mixed population of resident alveolar and interstitial macrophages, as well as recruited monocytes^4^. Macrophages are also accompanied by the arrival of neutrophils which migrate to the lung in response to chemotactic signaling and increased epithelial permeability specific to ARDS^5,6^. Macrophages and monocytes shape the trajectory of ARDS both early in the disease course^5,7,8^ and later in lung injury resolution^9,10^, and have both protective and pathogenic roles in experimental ARDS models^11,12^.

In addition to orchestrating the immune response, macrophages are master regulators of tissue iron homeostasis^13^. While iron levels vary across different organs, tissue-resident macrophages possess complex iron-handling mechanisms that carefully regulate iron in the extracellular milieu^14^. This regulation is crucial because insufficient intracellular iron disrupts normal cellular function, while excess free iron can catalyze the formation of oxygen and nitrogen radicals, leading to the production of phospholipid peroxides^15^. These peroxides in turn propagate along biological membranes and trigger a regulated cell death pathway termed ferroptosis^16^. Although the precise roles of these iron-catalyzed processes in ARDS pathogenesis remain unclear, there are observational and experimental data to support a connection between macrophage iron handling and ARDS^17–20^. Specifically, iron and iron-related proteins are increased in the lower respiratory tract of individuals with ARDS^21^, and macrophage or neutrophil iron loading increases injury in experimental ALI models^22–24^. One possible way iron may contribute to ARDS pathology involves the iron storage protein ferritin. Intracellular ferritin is a heteropolymer composed of ferritin heavy chain (FTH1) and ferritin light chain (FTL) subunits and is induced by cellular iron loading as a way to sequester intracellular iron in a non-toxic form^25^. FTH1 in particular is important and essential for survival, as demonstrated by the lethal effects of ferritin gene deletion in murine embryos^26,27^. Systemic loss of FTH1 in adulthood leads to severe multi-organ damage which is fatal in mice^28^, and mutations in critical *FTH1* functional regions are likely to be fatal in humans. Ferritin is also secreted into the extracellular space^29^ where it functions as a short-range cell-to-cell carrier of ferric iron^30–32^. This extracellular ferritin (ex-ferritin) is also composed of FTH1 and FTL and likely similarly regulated by iron, and has immunomodulatory and myelopoiesis-altering properties^33,34^. In multiple studies, elevated serum ex-ferritin levels precede clinical deterioration and are linked to adverse outcomes in individuals with ARDS^35–39^. Nevertheless, the biological role of ex-ferritin in ARDS is unknown, and whether increased ex-ferritin is merely a non-specific feature of inflammation and critical illness or whether there is a mechanistic reason for its production remain undetermined.

In this study, we hypothesized that macrophage ferritin regulation and secretion play a mechanistic role in the dysregulated systemic inflammatory response commonly observed in ARDS. Using three COVID-19-associated ARDS cohorts combined with mechanistic studies in murine ALI models, we show that ferritin is central to the response of macrophages to acute lung injury both via cell-intrinsic ferritin expression as well as active ferritin secretion. We propose that macrophage ferritin is upregulated in response to lung injury as a protective mechanism to regulate iron homeostasis and limit iron-driven cell death, such as ferroptosis. Furthermore, we suggest that the secretion of ferritin into the extracellular space represents an adaptive response, both to sequester excess extracellular iron and to modulate the innate immune response during tissue injury. Importantly, the composition of ex-ferritin appears to be functionally relevant, with FTL‑enriched ex‑ferritin conferring protection from injury in experimental models, highlighting context‑dependent effects across systems. Our results implicate macrophage ferritin as a crucial regulator of immune activation and macrophage survival in ARDS, and suggest that macrophage ferritin metabolism may be an important and potentially targetable pathway in ARDS pathogenesis.

## Results

### Serum ferritin trajectory associates with increased mortality in COVID-19 ARDS

**Fig. 1:**
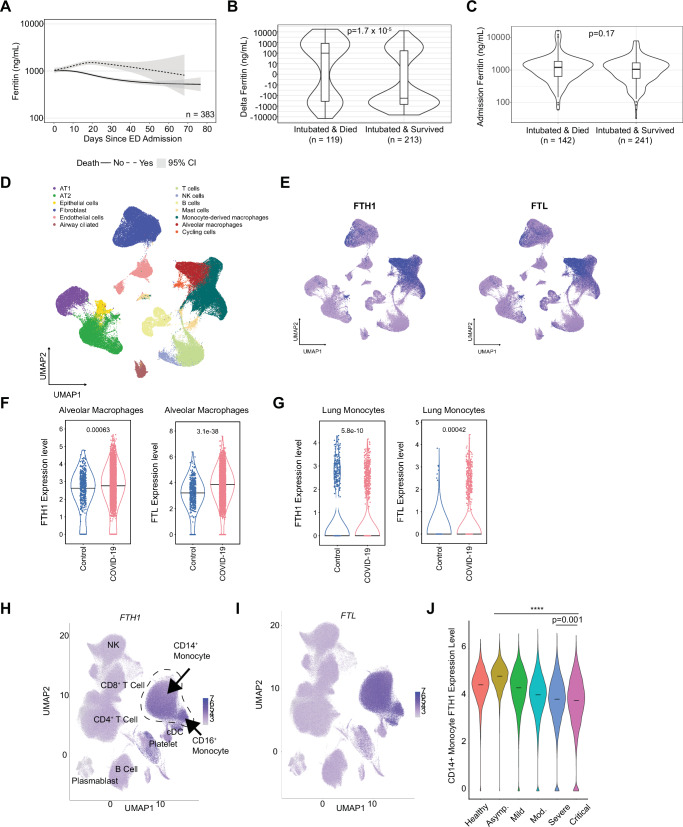
Serum ferritin associates with mortality in patients with COVID-19 ARDS. **A** Serum ferritin trajectory (with gray band indicating 95% confidence interval) in patients who were intubated due to respiratory failure from severe COVID-19 in those who survived (*n* = 241, solid line) and those who died (*n* = 142, dashed line). **B** Delta ferritin, defined as last serum ferritin level within 21 days of hospitalization subtracting ferritin upon ICU admission; and **C** ferritin level upon ICU admission, dichotomized by mortality in the NYP COVID-19 Cohort. **D** UMAP representation of *FTH1* and *FTL* (**E**) in main lung cell populations, with alveolar macrophage (**F**) and monocyte (**G**) expression presented as violin plots with medians. UMAP representation feature plot showing main peripheral blood cell types across all donors and expression of *FTH1* (**H**) and *FTL* (**I**). **J** Violin plots of CD14^+^ Monocytes of *FTH1* expression by donor cells across increasing covid severity. Data (**B**, **C**) presented using violin plots with median and box indicating upper and lower quartiles. *p* values calculated by Kruskal–Wallis test. ****p < 0.0001.

We first assessed if increased extracellular ferritin (ex-ferritin) levels correlated with ARDS in a large clinical cohort (*n* = 1408) of individuals hospitalized with COVID-19 at New York Presbyterian (NYP) Weill Cornell Medicine Center and New York Presbyterian Lower Manhattan Hospital. A total of 1102 of these hospitalized individuals had one or more serum ex-ferritin measurements, with 383 requiring mechanical ventilation for severe hypoxemic respiratory failure with bilateral infiltrates on imaging, meeting the Berlin criteria^2^ for ARDS (Supplementary Fig. 1A and Supplementary Table 1). On average, patients who were intubated and died from COVID-19 had a rising serum ex-ferritin trajectory during their hospital stay compared to patients who were intubated but survived (Fig. 1A). This difference in trajectory can be quantified by comparing delta ex-ferritin, the difference between a subject’s initial ex-ferritin at admission and their last ex-ferritin value before day 21 of hospitalization (illustrated in Supplementary Fig. 1B). The median delta ex-ferritin among individuals who were intubated and died (*n* = 119) was positive (+96.6 ng/mL), whereas the median delta ex-ferritin who were intubated and survived was significantly negative (−181.1 ng/mL, *n* = 213, *p* < 0.0001, Fig. 1B), despite the subgroups having similar initial ex-ferritin levels upon intensive care unit (ICU) admission (1209.4 ng/mL died vs. 1055.5 survived, *p* = 0.17) (Fig. 1C). Individuals who had delta ex-ferritin above the median had significantly higher 28-day mortality (37%) compared to subjects who had a delta ex-ferritin below the median (18%, *p* < 0.001), as well as fewer ventilator-free days (*p* = 0.019), despite having similar ventilator parameters at ICU admission and similar rates of COVID-19 related complications (Table 1). Adjusting for age, sex, and comorbidities such as obesity, diabetes, active malignancy, and coronary artery disease, serum ferritin was an independent predictor of 28-day mortality (adjusted odds ratio 1.012, confidence interval 1.00–1.022, *p* = 0.0139). To rule out the possibility that this phenomenon was exclusive to ARDS due to COVID-19, serum ferritin values were retroactively examined in subjects with (*n* = 447) and without (*n* = 3232) ARDS measured within 3 days of ICU admission using the Critical carE Database for Advanced Research (CEDAR) Biobank^40,41^. Individuals with ARDS had significantly higher serum ferritin (774.1 ng/mL) compared to those without ARDS (223.0 ng/mL, *p* < 0.0001, Supplementary Fig. 1C). These data show that ex-ferritin levels rise in ARDS and associate with worse clinical outcomes.

**Table 1:** Clinical outcomes by delta ferritin in ICU patients

Parameter/Ferritin (ng/mL)	Above median delta ferritin (*n* = 182)	Below median delta ferritin (*n* = 182)	*p* value
Delta ferritin	+398 (85, 921)	−512 (980, −220)	<0.001
28-day mortality *n* (%)	67 (37%)	33 (18%)	<0.001
Days free from ventilator	0 (0, 9)	0 (0, 14)	0.019
P/F ratio	121 (86, 174)	112 (86, 167)	0.4
Driving pressure	15 (12, 18)	14 (11, 17)	0.3
SOFA (day on intubation)	11 (10, 13)	11 (11, 13)	0.2
SOFA (72 h)	11 (10, 13)	11 (9, 12)	0.2
Renal replacement therapy *n* (%)	49 (27%)	38 (21%)	0.2
Venous thromboembolism *n* (%)	44 (24%)	39 (21%)	0.5

Data are presented as *n* (%) or median (interquartile range). Comparisons between groups were performed using Wilcoxon rank-sum tests for continuous variables and Pearson’s chi-squared tests for categorical variables. All tests were two-sided. *p* values were not adjusted for multiple comparisons.

*SOFA* sequential organ failure assessment, *PF* PaO2/FiO2.

### FTH1 and FTL expression are abundant in monocytes and lung macrophages in individuals with ARDS

The source of serum ex-ferritin in ARDS is unknown, but one of the leading candidates for ex-ferritin secretion is the macrophage^31,42^. We thus next examined the association between ferritin and macrophages in the setting of COVID-19^43^ by querying two available single-cell RNASeq (scRNA-Seq) datasets. Using a single-nucleus lung atlas of severe COVID-19 (containing 7 healthy non-COVID-19 controls who underwent lung resection or biopsy and 19 individuals with lethal COVID-19)^44^, we found that lung monocytes and macrophages had the highest abundance in expression of *FTH1* and *FTL* compared to other immune and structural cells in the lung (Fig. 1D, E). Lung resident macrophage *FTH1* and *FTL* expression were significantly higher in individuals who died from COVID-19 when compared to healthy controls^44,45^ (Fig. 1F). Conversely, lung monocyte *FTH1* and *FTL* expression were significantly lower in individuals with lethal COVID-19 when compared to healthy controls (Fig. 1G). We next analyzed an available peripheral blood scRNA-Seq dataset (containing 41 non-COVID-19 controls and 102 COVID-19 patients of varying severity, not exclusive to those with COVID-19 ARDS)^43^. Compared to other innate and adaptive immune cells such as NK cells, B cells, and T cells, monocytes (both classical CD14^+^ and nonclassical CD16^+^) had the highest abundance of *FTH1* and *FTL* transcripts (Fig. 1H, I). In CD14^+^ classical monocytes, the expression of *FTH1* increased in asymptomatic COVID-19 patients compared to healthy controls, subsequently declining with increasing disease severity (adjusted *p* < 0.0001 for all comparisons except for between severe and critical subgroups for *FTH1*, for which *p* = 0.0011, Fig. 1J). This decrease was also observed for *FTL* in CD14^+^ monocytes (Supplementary Fig. 2A). A trend for lower monocyte *FTH1* expression was also observed in the subset of subjects in the NYP COVID-19 cohort (*n* = 48 COVID-19 ARDS compared to *n* = 6 COVID-19 pneumonia) who had blood drawn for bulk RNA Sequencing (RNA-Seq) of peripheral blood mononuclear cells (PBMC) (Supplementary Fig. 2B). To correlate *FTH1* and *FTL* mRNA expression with protein expression, we analyzed blood monocyte FTH1 and FTL protein levels in control subjects (*n* = 3) and subjects with ARDS^46^ (*n* = 4), and observed an increase in classical monocyte FTH1 and FTL protein expression in subjects with ARDS (Supplementary Fig. 2C, D). The above findings reveal that FTH1 and FTL are abundant in myeloid cells within the lungs and peripheral blood of patients with severe COVID-19 and those with non-COVID-19 ARDS, and suggest both a local and systemic ferritin response to acute lung injury.

### Hyperoxia-induced ARDS modulates macrophage ferritin expression and extracellular ferritin

**Fig. 2:**
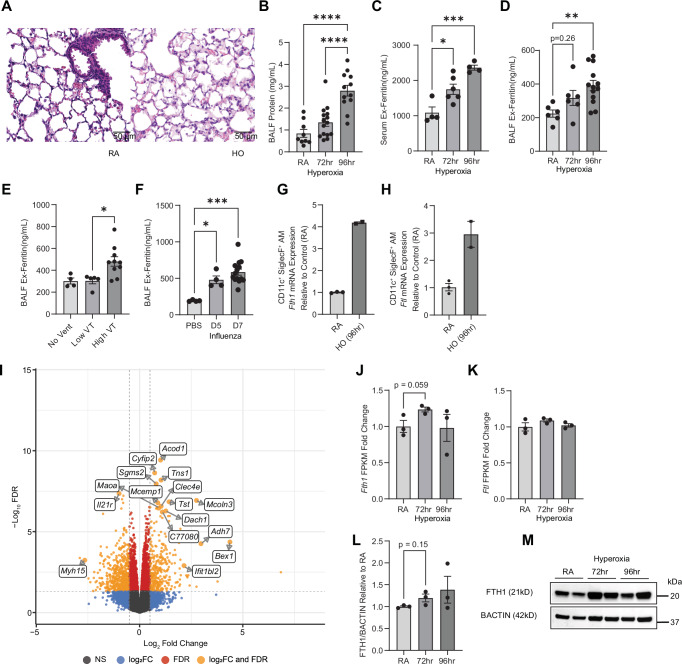
Hyperoxia-induced lung injury (HALI) as a murine ARDS model. **A** Representative immunohistochemical hematoxylin and eosin staining of lungs isolated from control mice (*Fth1*^*fl/fl*^) exposed to room air (RA) or 72 h of hyperoxia exposure, showing alveolar injury characterized by alveolar epithelial cell degeneration and necrosis, neutrophilic infiltrates in alveolar septum, and fibrillar proteinaceous material in alveolar spaces. Scale bars 50 µm. Bronchoalveolar lavage fluid (BALF) protein (**B**, *n* = 9/15/12 at RA/72 h/96 h timepoints, *p* < 0.0001 for RAv72 and 72v96), serum ex-ferritin (**C**, *n* = 4/6/4 at RA/72 h/96 h timepoints, *p* = 0.0122 for RAv72, *p* = 0.0002 for 72v96), and BALF ex-ferritin (**D**, *n* = 6/6/14 at RA/72 h/96 h timepoints, *p* = 0.0073 RAv96) with progressive hyperoxia exposure. **E** BALF ex-ferritin in unventilated and ventilated mice using low and high tidal volumes (VT) (*n* = 4/5/10 for unventilated/low VT/high VT conditions, *p* = 0.01). **F** BALF ex-ferritin in PBS-instilled control mice and mice instilled with influenza 5 days (D5) and 7 days (D7) post-instillation (*n* = 4/4/14 for PBS/D5/D7 timepoints, *p* = 0.019 PBSvD5, *p* = 0.0002 PBSvD7). mRNA expression of *Fth1* (**G**) and *Ftl* (**H**) in flow sorted CD11c^+^SiglecF^+^ AMs isolated from *Fth1*^*fl/fl*^ control mice following 96 h of hyperoxia exposure (*n* = 2 biological replicates), relative to RA expression (*n* = 3 biological replicates). *p* = 0.016 for *Ftl*. **I** Volcano plot highlighting gene expression (*n* = 3 for both groups) changes comparing bone marrow-derived macrophages (BMDMs) from *Fth1*^*fl/fl*^ mice exposed to 96 h of hyperoxia compared to those from RA *Fth1*^*fl/fl*^ mice, using a cut-off of false discovery rate (FDR) less than 0.05 and |log_2_FC | > 1, with genes meeting both cut-offs in orange, only the FDR cut-off in red, only the log_2_FC cut-off in blue, and neither cut-offs in black. BMDM expression of *Fth1* (**J**) and *Ftl* (**K**) in *Fth1*^*fl/fl*^ mice from the RA and 72- and 96-h hyperoxia conditions (*n* = 3 biological replicates) in Fragments Per Kilobase of transcript per Million mapped reads (FPKM), normalized to RA expression. **L** Quantified FTH1 protein expression in BMDMs from *Fth1*^*fl/fl*^ mice from RA and following 72 and 96 h of hyperoxia exposure (*n* = 3 for all conditions), with representative immunoblot in (**M**). Data (**B**–**H**, **J**, **L**) presented with mean ± SEM with *p* value by two-way analysis of variance (ANOVA) with Šídák’s correction for multiple comparisons, or by unpaired Student’s *t* test, as appropriate. *p < 0.05, **p < 0.01, ***p < 0.001, ****p < 0.0001.

Given the importance of lung macrophages including monocyte-derived lung macrophages to the pathophysiology of ARDS, we utilized a murine hyperoxia-induced acute lung injury (HALI) model^47–50^ to examine ferritin expression in alveolar macrophages (AM) and monocyte-derived macrophages during lung injury. HALI is a commonly used experimental model for ARDS^51^ whereby prolonged exposure to high oxygen ( > 95%) results in robust alveolar and bronchiolar epithelial cell injury (Fig. 2A). Such injury is characterized by alveolar and bronchiolar epithelial cell necrosis and degeneration along with neutrophils and macrophages infiltrating alveolar and peribronchiolar spaces. This results in increased alveolar capillary permeability characterized by increased bronchoalveolar lavage fluid (BALF) protein (Fig. 2B), eventually resulting in mortality starting approximately 72 h after hyperoxia onset. Notably, in this model, exposure to hyperoxia increased serum ex-ferritin in a time dependent manner (Fig. 2C), mirroring the increase in serum ex-ferritin with human ARDS. Similarly, BALF ex-ferritin also increased upon hyperoxia exposure, suggesting that a similar response is occurring in the lung microenvironment (Fig. 2D). BALF ex-ferritin levels also increased in mice ventilated using high tidal volumes (a ventilator-induced lung injury or VILI model)^52^, and had increased trends in two infection-associated lung injury models (influenza-induced and *Streptococcus pneumoniae*-induced) (Fig. 2E, F and Supplementary Fig. 3A).

To examine lung macrophage-specific ferritin expression, we isolated RNA from BALF cells and BALF-sorted Cd11c^+^ Siglec-F^+^ AM from room air (RA) mice and mice exposed to 96 h of hyperoxia. *Fth1* and *Ftl* mRNA expression were higher in the AM and BALF cells of mice exposed to hyperoxia, compared to those from RA mice (Fig. 2G, H and Supplementary Fig. 3B, C). *Fth1* mRNA expression was also significantly higher in AM isolated from mice exposed to intratracheal lipopolysaccharide (LPS, 6 h) by bulk RNA-Seq^53^ compared to those from untreated mice (Supplementary Fig. 3D). To assess transcriptomic changes in macrophages in the HALI model, we isolated bone marrow from mice exposed to 72 or 96 h of hyperoxia (*n* = 3 in each timepoint) along with RA control mice (Supplementary Fig. 4A). We differentiated bone marrow precursor cells to bone-marrow derived macrophages (BMDM) in vitro using M-CSF, then collected these cells and performed bulk RNA-Seq and analysis. Using cut-offs of false discovery rate (FDR) of 0.05, we found that hyperoxia induced significant gene expression changes in BMDM from mice exposed to 96 h of hyperoxia compared to those from RA mice, with 260 genes downregulated and 334 upregulated (Fig. 2I). GO (gene ontology) Biological Process analysis indicated that BMDM from mice exposed to 96 h of hyperoxia had increased expression of genes associated with response to increased oxygen levels as well as pathways involved in fatty acid binding and metabolism (Supplementary Fig. 4B). BMDM *Fth1* mRNA expression was increased following 72 h of hyperoxia and trended towards a decline with further exposure, while *Ftl* mRNA expression was unchanged (Fig. 2J, K). BMDM *Fth1* mRNA expression changes were mirrored by changes in FTH1 protein expression by immunoblotting (Fig. 2L, M). These findings indicate that hyperoxia significantly elevates FTH1 expression, and to a lesser extent FTL, in AM and in BMDM.

### Macrophage FTH1 deficiency protects against hyperoxia-induced lung injury

To examine the mechanistic role of FTH1 in ARDS, we generated a murine model of FTH1 depletion in myeloid cells using Cre-Lox recombination, placing *Fth1* under the control of the *Lyz2* promoter (*Fth1*^*ΔLysM*^, Supplementary Fig. 5A). Targeted *Fth1* depletion was confirmed in both BMDM as well as AM (Supplementary Fig. 5B–E) with no change in expression of FTH1 in CD45^−^ EpCAM^+^ alveolar epithelial cells^54^ (Supplementary Fig. 5F). FTH1 depletion did not affect BMDM growth, but in response to LPS stimulation, FTH1-depleted BMDM released lower levels of IL-6 and TNF-α (Supplementary Fig. 5G, H).

**Fig. 3:**
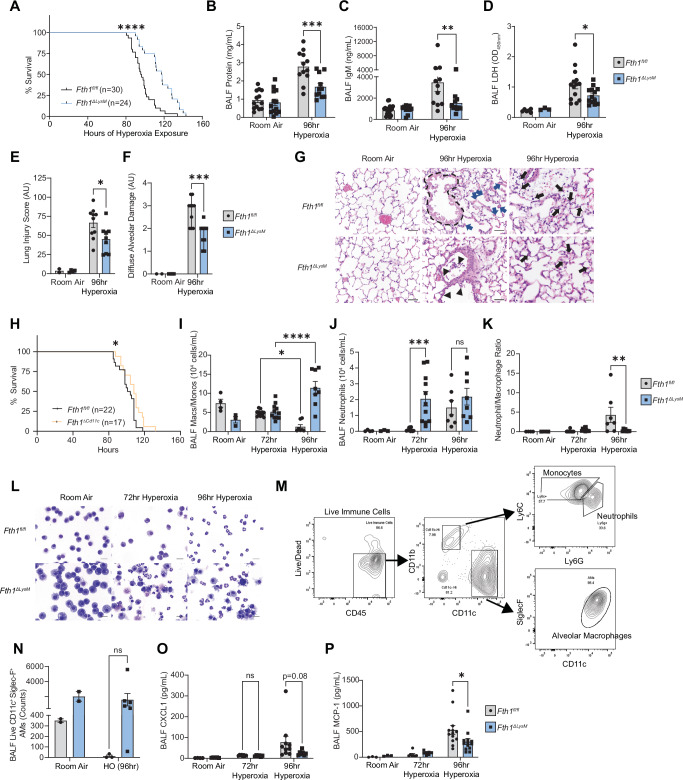
Myeloid FTH1-deficient mice are protected against hyperoxia-induced acute lung injury. **A** Percent survival of *Fth1*^*fl/fl*^ and *Fth1*^*ΔLysM*^ mice in hyperoxia with survival analysis. BALF protein (**B**, *n* = 13/12 for *Fth1*^*fl/fl*^ mice and *n* = 13/11 for *Fth1*^*ΔLysM*^ mice at the room air (RA) and 96 h time points, *p *= 0.0005 at 96 h), IgM (**C**, *n *= 21/11 for *Fth1*^*fl/fl*^ mice and *n *= 10/11 for *Fth1*^*ΔLysM*^ mice at the RA/96 h time points, *p *= 0.0021 at 96 h), and LDH (**D**, *n *= 6/14 for *Fth1*^*fl/fl*^ mice and *n *= 3/13 for *Fth1*^*ΔLysM*^ mice at the RA/96 h time points, *p *= 0.040 at 96 h) in *Fth1*^*fl/fl*^ and *Fth1*^*ΔLysM*^ mice exposed to hyperoxia for 96 h. Histological lung injury at 96-h measured by the Lung Injury Score (**E**) or diffuse alveolar damage (DAD, **F**) in arbitrary units (AU) presented as median with 95% confidence interval, *p* values by unpaired Student’s *t* test (*n *= 2/10 for *Fth1*^*fl/fl*^ mice and *n *= 4/9 for *Fth1*^*ΔLysM*^ mice at the RA/96 h time points for both measures, *p *= 0.026 for Lung Injury Score, *p *= 0.0004 for DAD). *p < 0.05, ***p < 0.001. **G** Representative hematoxylin and eosin staining of lung sections from mice exposed to hyperoxia or RA for 96 h (*n *= 2 *Fth1*^*fl/fl*^ mice and *n *= 4 *Fth1*^*ΔLysM*^ mice in RA, *n *= 10 *Fth1*^*fl/fl*^ mice and *n *= 9 *Fth1*^*ΔLysM*^ mice in hyperoxia). Dotted line highlights marked diffuse epithelial necrosis in a bronchiole with adjacent areas of alveolar epithelial cell necrosis and septal wall hyalinization (blue arrows). Arrowheads draw attention to multifocal foci of bronchiolar epithelial attenuation and necrotic debris in the lumen of a bronchiole. Black arrows indicate neutrophils in the alveolar septum and perivascular space. Scale bars indicate 40 µm in left and middle panels and 60 µm in right panels. **H** Percent survival of *Fth1*^*fl/fl*^ and *Fth1*^*ΔCd11c*^ mice in hyperoxia. BALF macrophages and monocytes (**I**, *n *= 4/12/6 for *Fth1*^*fl/fl*^ mice and *n *= 4/11/6 for *Fth1*^*ΔLysM*^ mice at the RA/72 h/96 h time points, *p *= 0.012 72v96 for *Fth1*^*fl/fl*^, *p *< 0.0001 for *Fth1*^*ΔLysM*^) and neutrophils (**J**, *n *= 4/12/6 for *Fth1*^*fl/fl*^ mice and *n *= 4/11/6 for *Fth1*^*ΔLysM*^ mice at the RA/72 h/96 h time points, *p *= 0.0006 for 72 h, *p *= 0.57 for 96 h) cell numbers, and neutrophil/macrophage ratio (**K**, *n *= 4/12/8 for *Fth1*^*fl/fl*^ mice and *n *= 4/11/8 for *Fth1*^*ΔLysM*^ mice at the RA/72 h/96 h time points, *p *= 0.0016 for 96 h) by manual counting of cytospin slides made from lavage cells from *Fth1*^*fl/fl*^ and *Fth1*^*ΔLysM*^ mice in RA and 72- and 96-h hyperoxia conditions, with representative ×40 magnification images in (**L**), scale bars indicate 20 µm. **M** Flow cytometry gating strategy and **N** counts of CD11c^+^ SiglecF^+^ AMs in *Fth1*^*fl/fl*^ and *Fth1*^*ΔLysM*^ mice exposed to RA or 96-h hyperoxia conditions (*n *= 2/3 for *Fth1*^*fl/fl*^ mice and *n *= 2/6 for *Fth1*^*ΔLysM*^ mice at the RA/96 h time points, *p *= 0.30 for 96 h). BALF CXCL1 (**O**, *n *= 4/8/10 for *Fth1*^*fl/fl*^ mice and *n *= 5/6/7 for *Fth1*^*ΔLysM*^ mice at the RA/72 h/96 h time points) and MCP-1 (**P**, *n *= 3/7/13 for both *Fth1*^*fl/fl*^ and *Fth1*^*ΔLysM*^ mice at the RA/72 h/96 h time points, *p *= 0.024 for 96 h). *p* values in survival studies by (**A**, **H**) Gehan-Breslow-Wilcoxon test. Data (**B**–**F**, **I**–**K**, **O**, **P**) presented as mean ± SEM, *p* values by two-way analysis of variance (ANOVA) with Šídák’s correction for multiple comparisons. *p  < 0.05, **p  < 0.01, ***p  < 0.001, ****p  < 0.0001.

In response to lung injury, *Fth1*^*ΔLysM*^ mice consistently survived longer in hyperoxia than littermate *Fth1*^*fl/fl*^ control mice, with a median survival of 118 h vs. 96 h (*p* < 0.0001, Fig. 3A). Pathological assessment of *Fth1*^*ΔLysM*^ mice at 96 h of hyperoxia exposure revealed that this difference in survival was associated with decreased lung injury. *Fth1*^*ΔLysM*^ mice had lower BALF protein and IgM compared to *Fth1*^*fl/fl*^ mice exposed to 96 h of hyperoxia, reflecting protection from alveolar capillary permeability (Fig. 3B, C). *Fth1*^*ΔLysM*^ mice had lower BALF lactate dehydrogenase (LDH) relative to *Fth1*^*fl/fl*^ mice exposed to 96 h of hyperoxia (Fig. 3D). These surrogate lung injury markers were consistent with histopathological changes in the lung, which showed decreased bronchiolar and alveolar epithelial cell necrosis and neutrophilic infiltrates in the alveoli and interstitial spaces as well as decreased histological diffuse alveolar damage in hyperoxia-exposed *Fth1*^*ΔLysM*^ mice when compared to *Fth1*^*fl/fl*^ mice, as quantified by a modified acute lung injury scoring system^51^ (Fig. 3E, F, representative images in Fig. 3G). These findings were recapitulated and validated in mice with a targeted deletion of *Fth1* in the resident alveolar macrophage population using a *Cd11c*^*Cre*^ driver^55^ whereby *Fth1*^*ΔCd11c*^ mice had improved survival in hyperoxia compared to littermate control *Fth1*^*fl/fl*^ mice (Fig. 3H). Additionally, *Fth1*^*ΔLysM*^ mice had trends for decreased weight loss and BALF protein in an influenza-induced ALI model (Supplementary Fig. 6A, B). These results demonstrate that macrophage FTH1 modulates lung injury and mortality arising from hyperoxia-induced lung injury.

### Macrophage FTH1 depletion alters the cellular inflammatory response to hyperoxia

To examine how macrophage FTH1 drives lung injury upon hyperoxia exposure, we next examined the effect of macrophage FTH1 depletion on the immune response to hyperoxia and on hyperoxia-induced cell injury. In addition to macrophages, neutrophils play a significant role in ARDS development^56^. In human ARDS, the number of neutrophils found in BALF is associated with the severity of gas exchange impairment and lung protein permeability^57^ as well as BALF inflammatory cytokine levels and degree of lung injury^58^. Therefore, we first quantified the amount of BALF macrophages and BALF neutrophils that infiltrated the airways upon hyperoxia using manual differential counting on BALF cell cytospin preparations. *Fth1*^*ΔLysM*^ mice, which had increased survival under hyperoxia initially displayed an early onset of neutrophil infiltration in the airspaces at 72 h when compared to *Fth1*^*fl/fl*^ controls (Fig. 3I, J). At 96 h, *Fth1*^*ΔLysM*^ mice had significantly higher numbers of macrophages compared to *Fth1*^*fl/fl*^ mice, whereas in contrast neutrophils were the dominant airway immune cell found in the *Fth1*^*fl/fl*^ mice (Fig. 3I, J). This neutrophil-macrophage imbalance was represented by a lower neutrophil to macrophage ratio in *Fth1*^*ΔLysM*^ mice when compared to the *Fth1*^*fl/fl*^ mice at 96 h (Fig. 3K, representative images Fig. 3L). Similar trends in BALF macrophage counts were also observed in *Fth1*^*ΔCd11c*^ mice exposed to hyperoxia (Supplementary Fig. 7A, B). Flow cytometry confirmed a trend for increased number of macrophages in *Fth1*^*ΔLysM*^ mice following hyperoxia exposure as well as a reduction in Cd11c^+^ SiglecF^+^ AM in *Fth1*^*fl/fl*^ mice after 96 h of hyperoxia exposure (Fig. 3M, N). BALF from *Fth1*^*ΔLysM*^ mice had lower levels of CXCL1, the principal neutrophil chemoattractant, as well as lower levels of macrophage/monocyte chemoattracts MCP-1 (CCL2), MCP-3 (CCL7), and MCP-5 (CCL12), compared to *Fth1*^*fl/fl*^ mice (Fig. 3O, P and Supplementary Fig. 8A, B).

### FTH1 depletion protects macrophages from ferroptosis

**Fig. 4:**
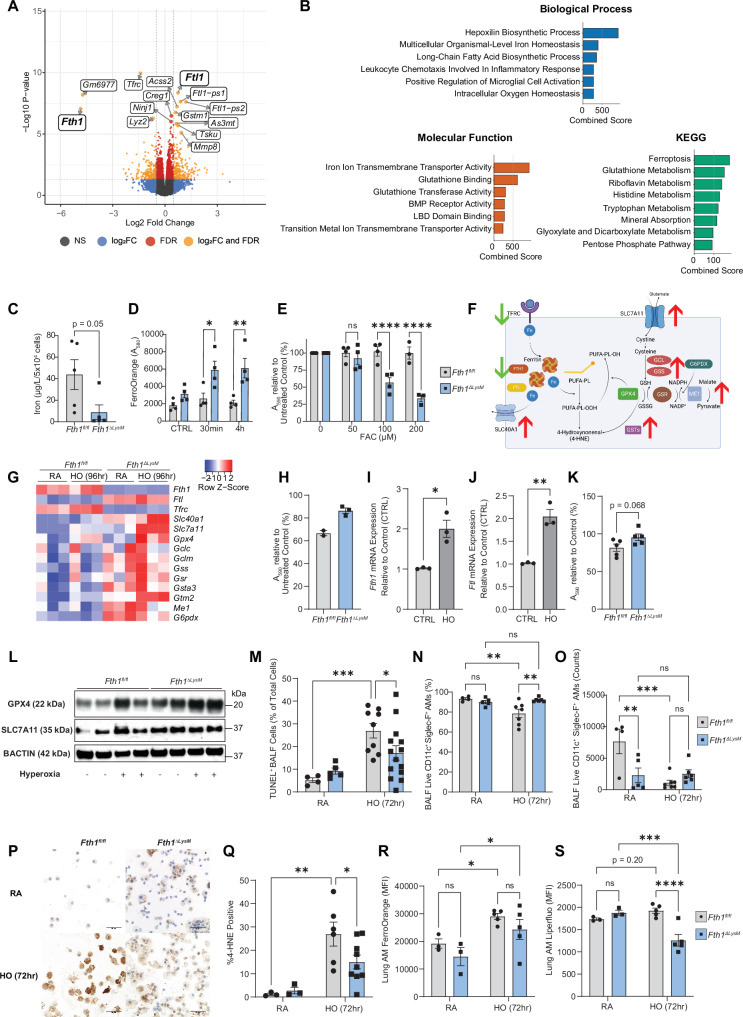
Myeloid FTH1-defiency protects against ferroptosis. **A** Volcano plot highlighting gene expression (*n *= 3 for all groups) changes comparing BMDM from (RA) *Fth1*^*fl/fl*^ and *Fth1*^*ΔLysM*^ mice exposed to 96 h of hyperoxia, using a cut-off of FDR less than 0.05 and |log_2_FC | > 1, with genes meeting both cut-offs in orange, only the FDR cut-off in red, only the log_2_FC cut-off in blue, and neither cut-offs in black. **B** Upregulated genes meeting the FDR cut-off analyzed using GO (biological process and molecular function) and KEGG, illustrated using Enrichr. **C** Total cellular iron, normalized to cell count, in BMDM isolated from *Fth1*^*fl/fl*^ (*n *= 5) and *Fth1*^*ΔLysM*^ mice (*n *= 5). **D** Fe^2+^ as measured by FerroOrange fluorescence at 580 nm (excitation 561 nm) in *Fth1*^*fl/fl*^ (*n *= 4) and *Fth1*^*ΔLysM*^ mice (*n *= 4) BMDM at baseline and 30 min or 4 h after 100 µM ferric ammonium citrate (FAC) treatment. *p *= 0.013 for 30 min, *p *= 0.0027 for 4 h. **E** Viability as measured by Alamar Blue fluorescence intensity at 590 nm (A_590_) of *Fth1*^*fl/fl*^ and *Fth1*^*ΔLysM*^ BMDM treated with FAC at 0, 50 µM, 100 µM and 200 µM concentrations for 24 h compared to untreated cells, *n *= 4 for all groups except for *n *= 3 at the 200 µM FAC concentration. *p *= 0.82 for 50 μM. Schematic (**F**) and heatmap (**G**) of critical proteins involved in iron-handling and ferroptosis, with their expression in BMDM from *Fth1*^*fl/fl*^ and *Fth1*^*ΔLysM*^ mice from room air and hyperoxia via Heatmapper (http://www.heatmapper.ca/expression/). Schematic created in BioRender. Cloonan, S. (2026) https://BioRender.com/l502xml. **H** Viability as measured by Presto Blue fluorescence intensity at 590 nm (A_590_) in BMDM from *Fth1*^*fl/fl*^ (*n *= 2) and *Fth1*^*ΔLysM*^ (*n *= 3) mice 4 h after treatment with 1 μM RSL3, relative to cells treated with DMSO. mRNA expression of *Fth1* (**I**) and *Ftl* (**J**) in *Fth1*^*fl/fl*^ and *Fth1*^*ΔLysM*^ BMDM (*n *= 3 for both groups) exposed to in vitro hyperoxia. *p *= 0.010 for *Fth1*, *p *= 0.0026 for *Ftl*. **K** Viability as measured by Presto Blue fluorescence intensity at 590 nm (A_590_) *in Fth1*^*fl/fl*^ and *Fth1*^*ΔLysM*^ BMDM (*n *= 5 for both groups) exposed to 24 h of in vitro hyperoxia as percentage of absorbance values of matched control cells. **L** Representative immunoblot of GPX4 and SLC7A11 protein expression in bronchoalveolar lavage fluid (BALF) cells from *Fth1*^*fl/fl*^ and *Fth1*^*ΔLysM*^ mice from RA and hyperoxia conditions (*n *= 2 in each group). **M** Total TUNEL^+^ immune cells on cytospin slides made from lavage cells from *Fth1*^*fl/fl*^ and *Fth1*^*ΔLysM*^ mice in room and 72-h hyperoxia condition (*n *= 4/9 for *Fth1*^*fl/fl*^ mice and *n *= 5/14 for *Fth1*^*ΔLysM*^ mice at the room air and hyperoxia time points, *p *= 0.0017 RAv72 in *Fth1*^*fl/fl*^ mice). Proportions (**N**) and counts (**O**) of BALF live AMs on flow cytometry (*n *= 4/7 for *Fth1*^*fl/fl*^ mice and *n *= 5/6 for *Fth1*^*ΔLysM*^ mice at the RA/72 h time points for both measures). *p *= 0.74 and *p *= 0.0049 for *Fth1*^*fl/fl*^ vs. *Fth1*^*ΔLysM*^ mice in RA and 72 h HO, respectively, *p *= 0.0066 and *p *= 0.86 for RAv72hr for *Fth1*^*fl/fl*^ and *Fth1*^*ΔLysM*^ mice, respectively. Representative 4-hydroxynonenal staining by immunocytochemistry (**P**) on BAL cell cytospins from *Fth1*^*fl/fl*^ and *Fth1*^*ΔLysM*^ mice (*n *= 3/6 for *Fth1*^*fl/fl*^ mice and *n *= 3/9 for *Fth1*^*ΔLysM*^ mice at the RA/72 h time points) under room air and following hyperoxia exposure, quantified in (**Q**). Median fluorescence intensity of FerroOrange (**R**) and Liperfluo (**S**) dye signal in lung CD11c^+^ SiglecF^+^ AMs (*n *= 3/5 for both *Fth1*^*fl/fl*^ mice and *Fth1*^*ΔLysM*^ mice at the RA/72 h time points for both measures). For FerroOrange: *p *= 0.32 and *p *= 0.20 for *Fth1*^*fl/fl*^ vs. *Fth1*^*ΔLysM*^ mice in RA and HO, respectively, *p *= 0.032 and *p *= 0.032 for RAvHO for *Fth1*^*fl/fl*^ and *Fth1*^*ΔLysM*^ mice, respectively. For Liperfluo, *p *= 0.37 and *p *= 0.0001 for *Fth1*^*fl/fl*^ vs. *Fth1*^*ΔLysM*^ mice in RA and 72 h HO, respectively, *p *= 0.20 and *p *= 0.0008 for RAv72hr for *Fth1*^*fl/fl*^ and *Fth1*^*ΔLysM*^ mice, respectively. Scale bars indicate 50 µm. Data (**C**–**E**, **H**–**K**, **M**–**O**, **Q**–**S**) presented as mean ± SEM, *p* values by two-sided unpaired Student’s *t* test (**C**, **I**–**K**) and two-way analysis of variance (ANOVA) with Šídák’s correction for multiple comparisons (**D**, **E**, **M**–**O**, **Q**–**S**). *p < 0.05, **p < 0.01, ***p < 0.001, ****p < 0.0001.

We next explored whether the increased abundance of airspace macrophages and the lower neutrophil:macrophage ratios in the lungs of *Fth1*^*ΔLysM*^ mice in hyperoxia was due to increased macrophage survival. Alveolar macrophage cell death plays an important role in the response of the lung to injury with macrophage inflammation and cell death mutually influencing each other to create a self-amplifying loop that exacerbates inflammation^59–61^. To assess if protection from macrophage cell death played a role in the lower neutrophil:macrophage ratios observed in the *Fth1*^*ΔLysM*^ mice under hyperoxia, we isolated bone marrow from *Fth1*^*fl/fl*^ and *Fth1*^*ΔLysM*^ mice exposed to room air or hyperoxia (*n *= 3 in each group), and generated BMDM in vitro. Bulk RNA-Seq analysis using cut-offs of false discovery rate (FDR) of 0.05 revealed 101 differentially expressed genes in macrophages harvested from *Fth1*^*fl/fl*^ mice exposed to 96 h of hyperoxia compared to those from *Fth1*^*ΔLysM*^ mice exposed to 96 h of hyperoxia (28 down and 73 up, Fig. 4A). Aside from a decrease in *Fth1* expression, BMDM from *Fth1*^*ΔLysM*^ mice had increased expression of genes involved in glutathione metabolism and defense against ferroptosis, an iron-catalyzed cell death pathway^16^, both via GO analysis and pathway analysis via KEGG (Fig. 4B). Ferroptosis is a form of regulated cell death that is mediated by membrane lipid peroxides and is catalyzed by iron. Consistent with this, FTH1-deficient BMDM demonstrated significantly lower total iron levels measured by graphite furnace atomic absorption spectrometry (GFAAS) when compared to *Fth1*^*fl/fl*^ controls (Fig. 4C). Since total iron may not represent biologically redox active free iron (Fe^2+^), Fe^2+^ was quantified in *Fth1*^*fl/fl*^ and *Fth1*^*ΔLysM*^ BMDM at baseline and after treatment with iron (ferric ammonium citrate or FAC) using the FerroOrange fluorescent probe^62^. *Fth1*^*ΔLysM*^ BMDM had significantly increased Fe^2+^ at 30 min and 4 h after FAC treatment relative to *Fth1*^*fl/fl*^ BMDM (Fig. 4D). Furthermore, *Fth1*^*ΔLysM*^ BMDM were sensitive to exogenous iron with FAC rapidly reducing *Fth1*^*ΔLysM*^ BMDM viability relative to *Fth1*^*fl/fl*^ BMDM at 24 h (Fig. 4E).

We postulated that these observations result from the inability of FTH1-deficient BMDMs to sequester toxic free iron, thereby triggering compensatory protective responses against ferroptosis. Consistently, *Fth1*^*ΔLysM*^ BMDM showed decreased expression of the transferrin receptor (*Tfrc*) and increased expression of ferroportin (*Slc40a1*), which limit iron uptake and facilitate iron release, respectively (Fig. 4F, G). Additionally, the expression of two key proteins that safeguard against ferroptosis, glutathione peroxidase 4 (*Gpx4*), which reduces lipid peroxides to harmless alcohols using glutathione, and *Slc7a11* (also known as system Xc^−^ or xCT), a glutamate/cystine antiporter which is essential for glutathione synthesis, were both significantly increased in hyperoxia and in *Fth1*^*ΔLysM*^ BMDM when compared to *Fth1*^*fl/fl*^ BMDM isolated from mice exposed to hyperoxia (Fig. 4F, G). The glutathione synthesis pathway downstream of SLC7A11 was also upregulated, with increased transcriptomic expression of glutamate–cysteine ligase (*Gclc*), glutathione synthetase (*Gss*), glutathione reductase (*Gsr*), as well as several glutathione S-transferases (GSTs) which act on peroxidation products, in *Fth1*^*ΔLysM*^ BMDM, when compared to BMDM isolated from room air *Fth1*^*fl/fl*^ mice and *Fth1*^*fl/fl*^ mice exposed to hyperoxia (Fig. 4F, G). Glucose-6-phosphate dehydrogenase (*G6pdx*) and malic enzyme (*Me1*), which replenishes the NAPDH used to generate glutathione, were also upregulated in the *Fth1*^*ΔLysM*^ BMDM when compared to control *Fth1*^*fl/fl*^ BMDM (Fig. 4F, G).

We validated these expression differences in iron and anti-ferroptosis genes functionally. *Fth1*^*ΔLysM*^ BMDM had improved survival compared to *Fth1*^*fl/fl*^ BMDM following treatment with ras-selective lethal small molecule 3 (RSL3), a chemical ferroptosis inducer, as quantified by the Presto Blue Assay (Fig. 4H). In vitro hyperoxia induced BMDM *Ftl* and *Fth1* mRNA expression (Fig. 4I, J), similar to what was observed in vivo, with *Fth1*^*ΔLysM*^ BMDM being more resistant to in vitro hyperoxia-induced loss in cell viability compared to *Fth1*^*fl/fl*^ cells (Fig. 4K). BALF cells from *Fth1*^*ΔLysM*^ mice exposed to room air or hyperoxia conditions had higher protein levels of GPX4 and SLC7A11 when compared to BALF cells from *Fth1*^*fl/fl*^ mice (Fig. 4L). *Fth1*^*fl/fl*^ mice had increased numbers of TUNEL+ cells compared to *Fth1*^*ΔLysM*^ mice indicating more immune cell death in the *Fth1*^*fl/fl*^ mice (Fig. 4M). This was consistent with increased proportions and a trend for increased counts of live (Zombie NIR^-^) CD11c^+^ Siglec-F^+^ AMs as detected by flow cytometry in BALF of *Fth1*^*ΔLysM*^ mice following hyperoxia exposure when compared to *Fth1*^*fl/fl*^ mice (Fig. 4N, O and Supplementary Fig. 9A). A non-significant trend of increased live neutrophil (CD11b^+^ Ly6G^+^) counts in *Fth1*^*ΔLysM*^ mice exposed to hyperoxia compared to control mice was observed, with no differences in the percentage of live neutrophils (Supplementary Fig. 9B, C). Supporting a connection with ferroptosis, BALF cells from *Fth1*^*ΔLysM*^ mice following hyperoxia exposure also had decreased immunohistochemical staining of 4-hydroxynonenal (4-HNE), a lipid peroxidation byproduct from ferroptosis, when compared to those from *Fth1*^*fl/fl*^ mice (representative images in Fig. 4P and quantified in Fig. 4Q). Whole lung AMs isolated from both *Fth1*^*fl/fl*^ and *Fth1*^*ΔLysM*^ mice exposed to hyperoxia had significantly increased Fe^2+^ levels as measured by FerroOrange compared to matched RA mice, while AMs isolated from *Fth1*^*ΔLysM*^ mice had a non-significant trend for lower FerroOrange staining compared to AMs from *Fth1*^*fl/fl*^ mice in both RA and hyperoxia conditions (Fig. 4R). Lipid peroxide species, as detected by Liperfluo dye^63^, were significantly lower in lung AMs from hyperoxia exposed *Fth1*^*ΔLysM*^ mice when compared to AMs from hyperoxia exposed *Fth1*^*fl/fl*^ mice (Fig. 4S). Taken together, the above data shows that a loss in macrophage FTH1 increases the resistance of macrophages to ferroptosis and may be one potential mechanism by which FTH1 deficient macrophages are protected from cell death in hyperoxia-induced experimental ARDS.

### The intracellular iron chelator deferiprone and the ferroptosis inhibitor liproxstatin-1 do not protect against hyperoxia-induced lung injury

**Fig. 5:**
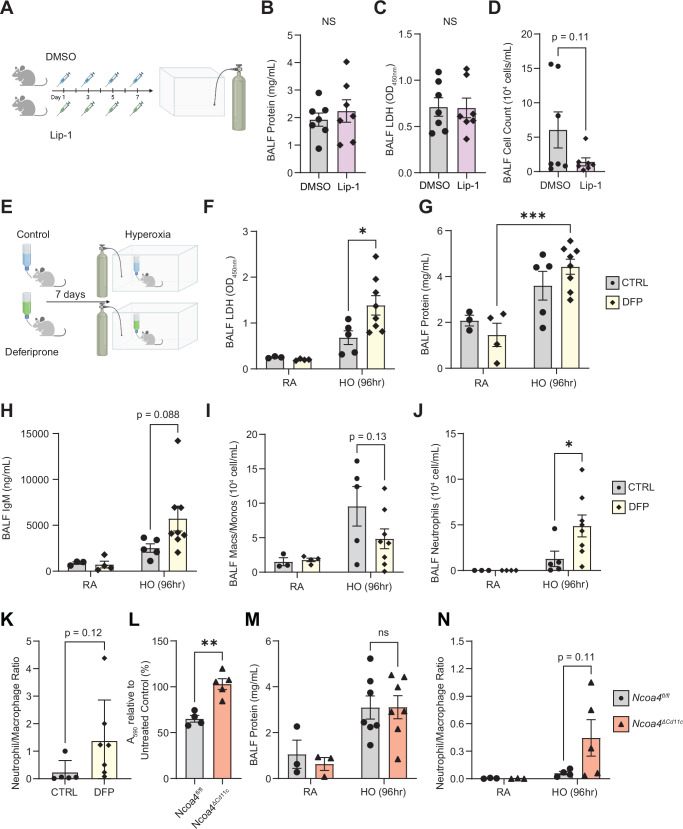
Systemic and macrophage ferroptosis inhibition do not protect against HALI. **A** Schedule of intraperitoneal liproxstatin-1 (Lip-1) injection prior to hyperoxia exposure. Created in BioRender. Cloonan, S. (2026) https://BioRender.com/0jcsk09. BALF protein (**B**), LDH (**C**), and total cell count (**D**) in untreated and Lip-1-treated wildtype mice exposed to hyperoxia (*n *= 7 in both groups). *p *= 0.52 for BALF protein and *p *= 0.95 for BALF LDH. **E** Administration of deferiprone (DFP) using drinking water prior to hyperoxia exposure. Created in BioRender. Cloonan, S. (2026) https://BioRender.com/ps47pu9. BALF LDH (**F**), protein (**G**), IgM (**H**) in wildtype mice untreated and given deferiprone (DFP) in room air (RA) or hyperoxia (HO) conditions, *n *= 3/4/5/8 in untreated RA, DFP RA, untreated HO, DFP HO groups for all experiments. *p *= 0.022 for BALF LDH CTRLvDFP in HO. *p *= 0.0005 for BALF Protein RAvHO in DFP-treated mice. BALF macrophage/monocyte count (**I**), neutrophil count (**J**, *n *= 3/5 for control mice and *n *= 4/8 for mice treated with DFP at the RA/96 h time points for both measures), and neutrophil/macrophage ratio (**K**) in untreated (*n *= 5) and DFP-treated (*n *= 7) mice exposed to 96 h of hyperoxia. *p *= 0.038 for BALF neutrophils CTRLvDFP in HO. **L** Viability as measured by Alamar Blue fluorescence at 590 nm (A_590_) of AM from *Ncoa4*^*fl/fl*^ (*n *= 4) and *Ncoa4*^*ΔCd11c*^ (*n *= 5) mice treated with 1 μM RSL4 for 4 h, relative to untreated cells, *p *= 0.0014. BALF protein (**M**) and neutrophil/macrophage ratio (**N**) in *Ncoa4*^*fl/fl*^ and *Ncoa4*^*ΔCd11c*^ mice in RA and hyperoxia conditions (*n *= 3/7 for *Ncoa4*^*fl/fl*^ and *Ncoa4*^*ΔCd11c*^ mice in RA/hyperoxia). *p *= 0.99 for BALF protein *Ncoa4*^*fl/fl*^ vs. *Ncoa4*^*ΔCd11c*^ in HO. Data (**B**–**D**, **F**–**N**) presented as mean ± SEM with *p* values by two-sided unpaired Student’s *t* test (**B**–**D**, **K**, **L**) or two-way analysis of variance (ANOVA) with Šídák’s correction for multiple comparisons (**F**–**J**, **M**, **N**), as appropriate. *p  < 0.05, **p  < 0.01, ***p < 0.001.

To assess if inhibiting ferroptosis pharmacologically could alter outcomes in experimental ARDS, we administered wildtype C57/BL6 mice with liproxstatin-1 (Lip-1, 10 mg/kg), which protects against ferroptosis by neutralizing reactive lipid radicals^64^ prior to hyperoxia exposure (Fig. 5A). Intraperitoneal Lip-1 administration had no effect on BALF protein and LDH of wildtype mice exposed to hyperoxia, although there was a trend for decreased BALF total cell counts in mice treated with Lip-1 following hyperoxia (Fig. 5B–D). Lip-1 failed to alter the survival of treated mice and had no effect on weight loss (Supplementary Fig. 10A, B). Similarly, intraperitoneal ferrostatin-1 administration (Fer-1) also did not protect C57/BL6 mice from hyperoxia exposure (Supplementary Fig. 10C, D).

Ferroptosis can also be curtailed with the use of ion chelators^16,65^. Although HALI is a lung injury model, serum hepcidin levels increased in *Fth1*^*fl/fl*^ mice following 72 h of hyperoxia exposure, with *Fth1*^*ΔLysM*^ mice having even higher serum hepcidin compared to *Fth1*^*fl/fl*^ mice. *Fth1*^*ΔLysM*^ mice also had higher BALF hepcidin compared to *Fth1*^*fl/fl*^ mice (Supplementary Fig. 11A, B), suggesting that hyperoxia exposure exerts an effect on systemic iron handling. Therefore, we next administered the intracellular iron chelator deferiprone (DFP) systemically (1 mg/mL in drinking water) to wildtype C57/BL6 mice 1 week prior to and for the duration of hyperoxia exposure (Fig. 5E). DFP reduced macrophage iron in vitro (Supplementary Fig. 12A) and reduced systemic iron in as short as 11–14 days in various animal organ injury models^66–68^. In this study, 1 week of DFP treatment did not affect hemoglobin levels (Supplementary Fig. 12B), but exacerbated lung injury following hyperoxia exposure, as evidenced by a significant increase in BALF LDH and a trend toward elevated BALF protein and IgM levels (Fig. 5F–H). DFP-treated mice also had decreased absolute BALF macrophage and monocyte counts and increased neutrophils (Fig. 5I, J), resulting in an increased neutrophil:macrophage ratio (Fig. 5K).

### Impaired macrophage ferritin recycling does not protect against HALI

The above results suggest that targeting ferroptosis systemically, via either inhibition of lipid peroxidation or iron chelation, does not protect from experimental ARDS in vivo. We next sought to ascertain if macrophage ferritin breakdown, a major source of intracellular iron and a regulator of ferroptosis, plays a role in HALI^69^. Ferritin degradation occurs via a selective autophagic process termed ferritinophagy, and is mediated by the autophagy adapter protein nuclear receptor coactivator 4 (NCOA4). NCOA4 is increased by intracellular iron depletion^70^ and binds to FTH1 and chaperones it to the autophagosome for the liberation of iron^69,71^. In vitro loss of NCOA4 is protective against chemically-triggered ferroptosis^69^, and in vivo disruption of the NCOA4-FTH1 interaction ameliorates ferroptosis-mediated ischemia-reperfusion injury^72^. We hypothesized that inhibiting this process could also protect macrophages from hyperoxia-induced ferroptosis and thus, ameliorate lung injury. Using *Cd11c*^*Cre*^, we depleted *Ncoa4* in lung macrophages in vivo, confirming loss of *Ncoa4* in AM (Supplementary Fig. 13A). AM from *Ncoa4*^*ΔCd11c*^ mice had significantly higher *Tfrc* levels and a trend for higher *Fth1* (*p *= 0.07) expression (Supplementary Fig. 13B, C). Although AM from *Ncoa4*^*ΔCd11c*^ mice had similar total iron levels when compared to those from *Ncoa4*^*fl/fl*^ mice (Supplementary Fig. 13D), NCOA4-deficient AM were protected from RSL3-induced ferroptosis in vitro (Fig. 5L). However, contrary to our hypothesis, *Ncoa4*^*ΔCd11c*^ mice were not protected from hyperoxia-induced lung injury compared with *Ncoa4*^*fl/fl*^ mice. Following 96 h of hyperoxia exposure, *Ncoa4*^*ΔCd11c*^ mice had similar BALF protein (Fig. 5M), with trends for decreased BALF total cell counts and macrophage counts, as well as a higher BALF neutrophil:macrophage ratio compared to *Ncoa4*^*fl/fl*^ mice (Fig. 5N and Supplementary Fig. 13E, F). These findings indicate that ferroptosis resistance alone is not sufficient to mitigate lung injury in this model. Taken together, these results show while FTH1-deficient macrophages exhibit enhanced protection from ferroptosis in vitro, inhibiting ferroptosis systemically or impairing AM ferritinophagy to limit intracellular iron availability for ferroptosis does not protect mice from hyperoxia-induced lung injury and may potentially exacerbate this injury.

### Loss of macrophage FTH1 results in lower extracellular iron and increased FTL ex-ferritin which is protective against hyperoxia

**Fig. 6:**
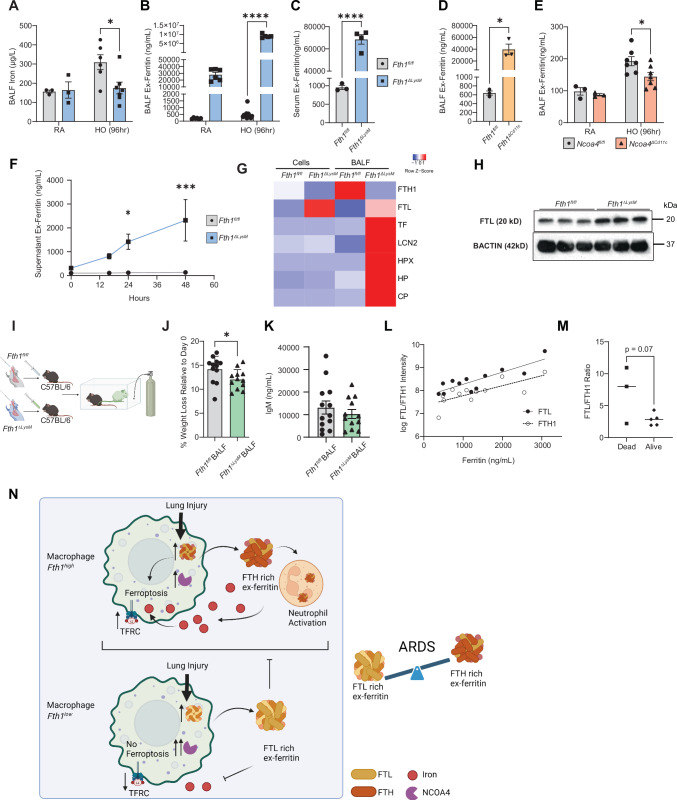
Extracellular ferritin controls extracellular iron levels and is protective against hyperoxia. **A** Total iron in BALF of *Fth1*^*fl/fl*^ and *Fth1*^*ΔLysM*^ mice at room air (RA) (*n *= 3 for both groups) and following 96 h of hyperoxia (*n *= 7 for *Fth1*^*fl/fl*^, *n *= 8 for *Fth1*^*ΔLysM*^ BALF), *p *= 0.0017 for *Fth1*^*fl/fl*^ vs. *Fth1*^*ΔLysM*^ mice in HO. BALF ferritin (**B**, *n *= 6/14 for *Fth1*^*fl/fl*^, *n *= 6/5 for *Fth1*^*ΔLysM*^ at RA/96 h hyperoxia conditions) and serum ferritin (**C**, *n *= 3 for *Fth1*^*fl/fl*^, *n *= 4 for *Fth1*^*ΔLysM*^) in *Fth1*^*fl/fl*^ and *Fth1*^*ΔLysM*^ in RA and hyperoxia conditions. BALF ferritin in *Fth1*^*fl/fl*^ and *Fth1*^*ΔCd11c*^ mice (**D**, *n *= 3 for both groups) and in *Ncoa4*^*fl/fl*^ and *Ncoa4*^*ΔCd11c*^ mice (**E**, *n *= 3 for both groups at RA, *n *= 7 for both groups at HO) at baseline and following hyperoxia exposure. *p *= 0.013 for *Fth1*^*fl/fl*^ vs. *Fth1*^*ΔCd11c*^ at RA and *p *= 0.024 for *Ncoa4*^*fl/fl*^ vs. *Ncoa4*^*ΔCd11c*^ in HO. **F** Ferritin measured via ELISA in media supernatant in culture of *Fth1*^*fl/fl*^ (*n *= 4) and *Fth1*^*ΔLysM*^ (*n *= 4) BMDM over time. *p *= 0.04 at 24 h and 0.0004 at 48 h. **G** Mass spectrometry proteomic analysis of *Fth1*^*fl/fl*^ and *Fth1*^*ΔLysM*^ AM and BALF. **H** Representative immunoblot of FTL in untreated *Fth1*^*fl/fl*^ and *Fth1*^*ΔLysM*^ BMDM. Each lane represents a separate biological replicate, samples isolated from 3 experiments. **I** Schematic of intratracheal BALF transplantation prior to hyperoxia exposure. Percent weight loss (**J**) and (**K**) BALF IgM levels in C57BL/6 mice transplanted with BALF from *Fth1*^*fl/fl*^ (*n *= 12) and *Fth1*^*ΔLysM*^ (*n *= 12) mice. *p *= 0.042 for weight loss and *p *= 0.43 for BALF IgM. **L** Ferritin heavy chain (FTH1) and light chain (FTL) intensity relative to serum ferritin from the same subjects, with FTL/FTH1 ratio stratified by patient survival (**M**, *n *= 3 dead and *n *= 5 alive). Data (**A**–**F**, **J**, **K**, **M**) presented as mea*n *± SEM, with *p* values calculated by unpaired Student’s *t* test (**C**, **D**, **J**, **K**, **M**) or 2-way analysis of variance (ANOVA) with Šídák’s correction for multiple comparisons (**A**, **B**, **E**, **F**) as appropriate.*p < 0.05, **p < −0.01, ***p < 0.001, ****p < 0.0001. **N** Overview schematic of FTH1/FTL balance modulating macrophage ferroptosis and neutrophil recruitment in lung injury. Created in BioRender. Cloonan, S. (2026) https://BioRender.com/oj3box6.

The above data suggested that there may be alternative mechanisms by which *Fth1*^*ΔLysM*^ mice are protected from HALI. Ferritin is predominantly known as an intracellular means of storing iron but is also known to be able to transport iron extracellularly^73^. We next assessed if changes in extracellular iron availability are linked to the protection from ferroptosis upon hyperoxia exposure in the *Fth1*^*ΔLysM*^ mice. By GFAAS, extracellular iron levels were significantly lower in the BALF of *Fth1*^*ΔLysM*^ mice when compared to *Fth1*^*fl/fl*^ mice 96 h after exposure (Fig. 6A). Significantly higher BALF ex-ferritin levels were detected by enzyme-linked immunosorbent assays (ELISA) in *Fth1*^*ΔLysM*^ mice at baseline, with *Fth1*^*ΔLysM*^ mice having over 1000-fold higher BALF ex-ferritin, rising further in response to hyperoxia (Fig. 6B). Serum ex-ferritin was also significantly higher in *Fth1*^*ΔLysM*^ mice when compared to *Fth1*^*fl/fl*^ mice at baseline (Fig. 6C). *Fth1*^*ΔCd11c*^ mice had similarly elevated BALF ex-ferritin levels at baseline (Fig. 6D), while in direct contrast, *Ncoa4*^*ΔCd11c*^ mice showed decreased BALF ex-ferritin levels in response to hyperoxia (Fig. 6E). NCOA4 is a major regulator of iron recycling in macrophages^74^, and NCOA4-deficient BMDM display an impaired iron recycling capacity in vivo and fail to degrade ferritin and export iron under conditions of iron deficiency^75^. Consistent with this, *Ncoa4*^*ΔCd11c*^ mice had similar BALF iron levels compared to *Ncoa4*^*fl/fl*^ in hyperoxia, whereas NCOA4 levels were elevated in FTH1-deficient BMDM when compared to *Fth1*^*fl/fl*^ mice (Supplementary Fig. 13G, H). In culture, higher levels of ex-ferritin were detected in BMDM cell culture supernatants in a time dependent manner in *Fth1*^*ΔLysM*^ mice when compared to *Fth1*^*fl/fl*^ mice (Fig. 6F). We hypothesized that this ex-ferritin was predominantly FTL as *Fth1*^*ΔLysM*^ BMDM have little to no FTH1 (Supplementary Fig. 5E). Using unbiased proteomics on *Fth1*^*fl/fl*^ and *Fth1*^*ΔLysM*^ AM and BALF, *Fth1*^*ΔLysM*^-derived BALF and BALF cells had significantly higher levels of FTL, as well as other iron-binding proteins when compared to *Fth1*^*fl/fl*^ BALF and BALF cells (Fig. 6G). BMDM isolated from *Fth1*^*ΔLysM*^ mice also had higher FTL expression by immunoblotting (Fig. 6H). The above data suggests that upon loss of FTH1 in macrophages, FTL is increased and secreted into the extracellular space and this is associated with lower extracellular iron levels during hyperoxia exposure.

To exclude the possibility this marked elevation in ferritin stems from a macrophage activation syndrome (MAS), which is often associated with very high serum ferritin (hyperferritinemia)^76^, we examined plasma blood counts and the liver and spleen of *Fth1*^*fl/fl*^ and *Fth1*^*ΔLysM*^ mice histologically in RA and hyperoxia conditions. While hyperoxia exposure was associated a decrease in leukocytes, this was not accompanied by a drop in hemoglobin (Supplementary Fig. 14A, B). There was also no evidence of hemophagocytosis in the liver and spleen of *Fth1*^*ΔLysM*^ mice (Supplementary Fig. 14C). Cytokines and chemokines associated with macrophage activation^77^ measured in the plasma from *Fth1*^*fl/fl*^ and *Fth1*^*ΔLysM*^ mice under RA and hyperoxia (72 h exposure) conditions were also similar (Supplementary Fig. 14D).

As mentioned, ferritin is a heteropolymer consisting of FTH1 and FTL chain subunits^25^. While structurally similar, FTH1 contains the ferroxidase center that converts Fe^2+^ to Fe^3+^ for iron incorporation into ferritin, whereas FTL enhances ferritin’s iron storage capacity^78^. Their ratio varies by tissue and modulates ferritin’s function^79^. We hypothesized that serum ex-ferritin, once thought to be a benign byproduct of cell damage, may instead reflect ferroptotic cell death and serve to sequester extracellular iron, limiting further ferroptosis^29^. Supporting this, extracellular FTL protects against ferroptosis in models such as lung adenocarcinoma and pre-eclampsia^80,81^ and recombinant FTL reduces inflammation in BMDM^82^. To test the hypothesis that a rise in FTL-ex-ferritin confers protection in the setting of hyperoxia-induced lung injury, we isolated BALF from *Fth1*^*fl/fl*^ and *Fth1*^*ΔLysM*^ mice and after centrifugation to remove BALF cells, intratracheally transplanted the supernatant fluid into C57/BL6 mice via intratracheal instillation, 2 h before exposing to hyperoxia (Fig. 6I). After 72 h of hyperoxia, mice receiving BALF (FTL-ex-ferritin high) from *Fth1*^*ΔLysM*^ mice had decreased weight loss compared to mice receiving BALF from *Fth1*^*fl/fl*^ mice (FTL-ex-ferritin low) (Fig. 6J) and a trend for decreased BALF LDH (Fig. 6K). The above findings suggest that in response to a loss of FTH1 macrophages up-regulate and secrete FTL into the extracellular space, with higher FTL levels in the extracellular space associating with lower extracellular iron levels and protection from HALI.

### Serum ex-ferritin consists predominantly of FTL in human ARDS

Increased ex-ferritin with low serum iron is linked to severe COVID-19^83^, though whether this is a maladaptive response that drives disease severity or is an adaptive one that ultimately fails is unclear. Serum ferritin in health is comprised almost exclusively of FTL and is generally thought to be iron poor^84^, but the makeup of serum ferritin in critical illness has yet to be examined. Given our above findings that FTH1 is increased in human and murine macrophages in ARDS, and that mice who have little macrophage FTH1 are protected from ferroptosis under hyperoxia likely due to the increased secretion of FTL which sequesters extracellular iron, we sought to determine the molecular composition of the increased serum ferritin in human COVID-19. We performed mass spectrometry on serum samples from subjects in our COVID-19 cohort (*n *= 12). Taking advantage of ferritin’s stability at high temperatures^85^, we heated serum samples to denature other proteins, thereby enriching serum for ferritin. We then isolated ferritin using native gel electrophoresis, whereupon under the guidance of a native gel ladder and liver ferritin standard, we excised two Coomassie blue-stained bands within the kDa range of native polymeric ferritin (black arrow heads, Supplementary Fig. 15A) and analyzed them using mass spectrometry. Both FTH1 and FTL mirrored the increase in clinically measured ferritin in the matched subjects (Fig. 6L). We found that native serum ferritin protein in COVID-19 patients was composed of approximately 75.4 ± 10.35% FTL and 24.6 ± 10.35% FTH1 (Supplementary Fig. 15B), and that in individuals with ARDS who died had a trend for higher levels of FTL when compared to patients with ARDS who survived (Fig. 6M). Both FTH1 and FTL did not correlate with serum iron levels in matched samples (Supplementary Fig. 15C). These data indicate that the elevated serum ferritin in COVID-19 is predominantly a higher ratio of FTL:FTH1 and thus may represent an adaptive response to injury that has failed. Therefore, the trajectory of how the molecular composition of serum ferritin changes over the course of disease may have clinical significance and reflect the underlying biological changes we observe in our murine studies (Fig. 6N).

## Discussion

In this study, we link macrophage ferritin metabolism to the regulation of the immune response and the development of lung injury in ARDS. Using a large COVID-19-associated ARDS data set, we first show high extracellular ferritin levels in the serum associate with increased mortality in intubated COVID-19 patients with ARDS. These findings are consistent with the findings of others showing that higher serum ferritin levels in individuals who developed respiratory failure or died from COVID-19^86,87^ or non-COVID-19 associated ARDS^39^. Elevated serum ferritin has long been observed in other inflammatory diseases and has largely been relegated to being a non-specific inflammatory marker with unclear effects. Here, we provide evidence that the regulation and balance of macrophage intracellular and secreted extracellular ferritin directly modulates lung inflammation and injury development in ARDS.

We show that ferritin transcripts are highly abundant in monocytes and macrophages in the lung and peripheral blood of patients with critical COVID-19 and that this enrichment associates with COVID-19 severity. Macrophage and neutrophil iron loading^23^ (which leads to increased intracellular ferritin) increases injury in experimental ALI models, but to our knowledge, macrophage ferritin expression and modulation has yet to be examined in COVID-19 or ARDS. To mechanistically interrogate the role of macrophage ferritin in the pathobiology of ARDS in vivo, we utilized a HALI model to show that hyperoxia exposure increases lung macrophage ferritin expression which correlates with a rise in serum ex-ferritin in similar manner to human ARDS. Having replicated these findings in other ALI (bacterial, viral and ventilator-induced) models, we focused our efforts on ferritin heavy chain or FTH1 as this particular subunit was the most highly regulated in human and murine macrophages and is essential for survival. Critically, mice with a loss of FTH1 in myeloid cells (*Fth1*^*ΔLysM*^ mice) or resident lung (*Fth1*^*ΔCd11c*^) macrophages have improved survival in hyperoxia. *Fth1*^*ΔLysM*^ mice also have decreased lung injury severity outcomes as well as lower inflammatory markers and cytokine levels in the lung in response to hyperoxia. This protection from lung injury in the *Fth1*^*ΔLysM*^ mice was also suggested in an influenza-induced lung injury model. This is in line with previous studies that have shown that *Fth1*^*ΔLysM*^ mice are protected from mortality in a cecal ligation-and-puncture (CLP) model of sepsis^82^ as well as from diabetes in a high fat diet-induced obesity model^88^. Both of these prior studies showed dampened inflammatory activation as represented by macrophage cytokine expression and systemic cytokine levels. This is consistent with a study identifying FTH1 as a strong predictor of alveolar macrophage transcriptomic states during LPS-induced lung inflammation, suggesting its potential as a marker of monocyte-to-macrophage differentiation^89^.

We present two possible hypotheses for the mechanism of this protection. We first show that FTH1-deficient macrophages resist ferroptotic cell death, which in turn alters macrophage-neutrophil interactions in the injured lung. Prior studies have implicated lung macrophage death, including pyroptosis^90^ and necroptosis^91^ in mediating acute lung injury and ARDS, including COVID-19 ARDS^92^, amplifying lung inflammation through the generation of mediators and damage-associated molecular patterns (DAMPs) in a vicious cycle. Ferroptosis is another form of regulated programmed cell death and drugs or molecules that inhibit ferroptosis alleviate experimental lung injury in vitro and in vivo^93–95^. In some studies, ferritin is required for protection against ferroptosis^96^. In other studies, ferritin increases with ferroptosis-inducing agents^69^ and ferritin breakdown by ferritinophagy activates ferroptosis^97^. Iron-rich ex-ferritin can also be secreted in exosomes in response to ferroptotic stress^32,98^ or excess iron challenge^75^ to reduce intracellular iron levels, thereby helping cells become more resistant to ferroptosis. Hyperoxia induces ferroptosis in the lung^99^, and in vitro studies suggest that macrophage iron loading increases hyperoxia-induced cell death and impairs the capacity of macrophages to sequester iron in ferritin in vitro^22^. Our findings that a loss of FTH1 increases resistance to macrophage ferroptosis in vivo and that systemic iron chelation and inhibiting ferritinophagy exacerbates injury in response to hyperoxia suggest that FTH1 promotes macrophage ferroptosis in vivo. Others have also shown that ferroptotic death of macrophages and neutrophils worsens disease in models such as sepsis, cancer, and infection^100^. Although FTH1 is widely regarded as cytoprotective^101–103^, our findings highlight a context‑dependent role for macrophage ferritin. In non‑immune or acute depletion models, FTH1 limits oxidative damage by sequestering intracellular iron. In contrast, sustained loss of FTH1 in macrophages permits adaptive rewiring of iron handling pathways, reducing intracellular iron retention and engaging anti‑ferroptotic programs. Notably, this protection is conditional, as FTH1‑deficient macrophages exhibit increased sensitivity to exogenous iron, indicating a loss of intrinsic iron‑buffering capacity despite enhanced survival under hyperoxia-induced stress. To the best of our knowledge, this is the first study to specifically target macrophage ferritin and impair ferritin breakdown via NCOA4 in vivo and in the setting of ARDS. Although our findings suggest that FTH1-mediated ferroptosis promotes hyperoxia-induced lung injury, systemic interventions, such as treatment with the ferroptosis inhibitor liproxstatin-1/ferrostatin-1 or iron chelation failed to mitigate the damage. This suggests that macrophage-specific iron handling and ferritin trafficking, rather than ferroptosis inhibition per se, may be the dominant regulator of injury severity^104^. While these experiments provided valuable insights, further studies are warranted to refine macrophage-targeted strategies. Specifically, manipulating key ferroptosis regulators such as GPX4, SLC7A11, and other lipid peroxidation detoxification pathways in macrophages could deepen our understanding and potentially uncover more effective therapeutic avenues.

The second potential mechanism of protection of the *Fth1*^*ΔLysM*^ mice to hyperoxia involves macrophage ferritin secretion. Importantly, this protective phenotype was not linked to erythrophagocytosis or MAS markers, despite the link between hyperferritinemia with MAS and hemophagocytic lymphohistiocytosis clinically. We propose that ex-ferritin secretion serves as an early protective mechanism to relieve intracellular iron overload and prevent cellular injury^30^. When this response fails, such as in NCOA4 deficiency, cells may become more susceptible to ferroptotic death. Our findings align with previous reports suggesting that NCOA4 is activated by hyperoxia as a protective response and has a critical role in facilitating ferritin secretion, which we posit may be a protective repsonse^75,105^. While ferritin secretion was protective in our lung injury model, the form of the ex-ferritin secreted, specifically its FTH1/FTL composition, as well as the disease pathology, add to the modularity and complexity of this process. Prior studies have shown that ex-ferritin can trigger neutrophil extracellular trap-mediated cytokine storm in adult-onset Still’s disease^106^ and in sepsis-associated ALI and that ferritin-associated iron induces neutrophil dysfunction in patients with iron overload^107^. Others have shown that ferritin primes inflammasome activation in macrophages in vitro, and inflammasome activation results in increased ex-ferritin release^108–110^. Macrophage FTH1 also has been shown to modulate systemic iron homeostasis during infection^111^. Similarly locally organized and activated FTH1^high^ neutrophils aggravate lung injury in an IL-10-dependent manner^23^ supporting our hypothesis that FTH1 may also regulate neutrophil inflammation in ALI. The above studies do not directly link macrophages as the source of ex-ferritin, do not distinguish between FTH or FTL or the iron content of this ex-ferritin, and do not speculate what the receptors for ex-ferritin are. Our findings suggest that the balance between FTL and FTH1 ex-ferritin and the iron content of this ex-ferritin is important for the downstream signaling pathways induced as a result of ex-ferritin secretion by macrophages. While we did not measure ferritin bound iron in this study, serum FTH1 and FTL did not correlate with serum iron levels, consistent with previous studies^83^. Further studies are needed to assess whether it is the iron content of ex-ferritin or the “cytokine-like activity” of the ferritin subunits themselves that drives lung injury in ARDS.

Our finding that macrophages secrete FTL in response to a loss in FTH1 is an interesting finding that has been observed previously in *Fth1*^*ΔLysM*^ mice^82,88,112–115^ and may have important consequences for developing therapeutic agents that target ferroptosis. Ferritin does not contain a traditional signal reception particle motif and therefore is not secreted through the endoplasmic reticulum-Golgi apparatus system; instead, ferritin is secreted in nonclassical pathways via the multivesicular body-exosome system^31,42^ by macrophages, a process regulated by NCOA4^75^. Multiple receptors for FTH1 have been discovered in various cell types^116,117^, supporting the hypothesis that ferritin may not just be an intracellular iron storage complex and can be secreted intentionally. Although receptors for FTL have yet to be described, FTL is protective against ferroptosis in some disease models^80,81^ and has anti-inflammatory properties^82^, although myeloid-specific FTL depletion does not exacerbate sepsis-associated organ injury^118^. Our results suggest a similar trend, with decreased extracellular iron levels in our myeloid FTH1-deficient mice a plausible explanation for the protective role of FTL in ferroptosis by sequestering available extracellular iron, and a rationale which is consistent with the macrophage’s duty as a regulator of tissue iron homeostasis. Consistently, transplantation of FTL-enriched BALF into wildtype mice mitigated some of the effects of hyperoxia suggesting a possible therapeutic benefit that warrants further exploration. Notably, hyperferritinemia is observed in several other human diseases and is associated with worse disease pathology, but the structural composition of the extracellular ferritin polymer in COVID-19 and other hyperferritinemic diseases remains to be determined. Our study represents the first study to characterize extracellular ferritin in COVID-19-associated hyperferritinemia. Routine clinical laboratory measurements of “ferritin” are typically ELISA-based on and are more specific for FTL. We show that while serum ferritin in COVID-19 is predominantly composed of FTL, it also contained FTH1. We hypothesize that serum FTL in fatal ARDS likely reflects a compensatory response to overwhelming iron dysregulation, and may play a role in the regulation of ferroptosis of macrophages and perhaps other cells in ALI, such as type 2 alveolar epithelial cells (AT2) in which iron is known to modulate both injury and repair^119,120^. Notably, this hypothesis does not necessarily contradict the observation that higher serum ex-ferritin, and thus higher serum FTL, associating with worse outcomes in COVID-19 ARDS, as this FTL upregulation may represent a failed adaptive or rescue strategy for more severe disease.

There are some limitations to this study. Our primary COVID-19 cohort was built in a single US city, and while it includes subjects from both a quaternary academic center and a community hospital, the study population may not be representative of patients in other care settings, especially in rural and resource-limited settings. Our cohort was also built early in the pandemic (March through May 2020), when optimal care algorithms have yet to be developed for severe COVID-19 infections. We performed bulk RNA-Seq on BMDM following 3-days of M-CSF treatment. 3-days is a relatively short duration of M-CSF-driven differentiation from myeloid precursors and was chosen because we wanted to maintain the “training” effect of hyperoxia on myeloid progenitors^121^ as much as possible. Studies suggest that at as early as 3 days monocyte markers such as CD14, the human correlate to CCR2, can be identified on BMDM treated with M-CSF^122^, but whether these cells are representative of monocytes or monocyte-derived macrophages in our model is unclear. Nevertheless, we found significant differences between BMDM generated from mice in hyperoxia and those from room air mice, as well as similar transcriptional changes induced by hyperoxia between BMDM and BALF cells, demonstrating that there is retention of the exposure effect despite the ex vivo nature of the study. Whether NCOA4 expression increases, independently to its role in iron metabolism^123^ or whether *Fth1*^*ΔLysM*^ mice have an altered ability to interact with NCOA4 also remains to be examined.

In conclusion, in this study we propose that upregulation of macrophage ferritin and secretion of ex-ferritin by macrophages may serve as a crucial immunomodulatory mechanism by which macrophages not only protect themselves from ferroptosis but also ensure a regulated immune response to organ injury. These findings reposition extracellular ferritin from a passive inflammatory marker to an active immunometabolic regulator of ARDS pathogenesis. Additional work is needed to examine the manipulation of ferritin expression and secretion as a therapy for ARDS and other similar diseases.

## Methods

### New York Presbyterian COVID-19 cohort

The retrospective component of this study was approved by the institutional review board of Weill Cornell Medicine (WCM, Study Number: 20-03021689) and is in accordance with the Declaration of Helsinki. We identified patients who had confirmation of Sars-Cov-2 infection by reverse transcriptase polymerase chain reaction assays performed on nasopharyngeal swabs. Subjects included in our cohort were 18 years of age or older and had an emergency room visit or hospitalization with admission dates between March 3, 2020, and May 15, 2020, at two hospitals in New York City: New York Presbyterian-Weill Cornell Medicine, an academic quaternary care center, and New York Presbyterian-Lower Manhattan Hospital, an academic community hospital. For individuals with multiple admissions during this period, only data from the first admission were used. Subjects with “do-not-intubate” orders were excluded. The Weill Cornell Medicine Institutional Review Board approved the COVID-19 and ARDS studies (IRB; protocols 20-03021689 and 17-06018287). For the pre-COVID ARDS study, ICU visits from January 1, 2004 to February 29, 2020 were analyzed, excluding visits on or after March 1, 2020. Informed consent was waived as both studies were retrospective, involved no increased risk outside of routine care, no prospective sample collection, and analyzed clinical biomarkers obtained as part of standard care; many individuals were discharged or deceased, precluding consent. ARDS was defined using the Berlin criteria in all cases.

#### Data collection

Relevant data were manually abstracted from the electronic health record by trained research personnel using a quality-controlled protocol and structured abstraction tool^124^, comprising demographic data, clinical characteristics, vital signs, comorbidities, laboratory measurements, and other relevant patient data. Additional data were collected from the Weill Cornell-Critical Care Database for Advanced Research (WC-CEDAR) and the Weill Cornell Medicine COVID Institutional Data Repository (COVID-IDR)^41,125^.

### Single-cell RNA-seq analysis

#### Lung macrophages

Single-nucleus RNA-seq data from lung tissues of 19 COVID-19 decedents and 7 control patients were retrieved from the Gene Expression Omnibus (GEO) under accession number GSE171524^44^. Gene-UMI count matrices from individual samples were merged into a single dataset and processed using the Seurat package. Cells with fewer than 300 detected genes and genes expressed in fewer than 50 cells were excluded to remove low-quality data. Data normalization, variable gene identification, and cell-cycle phase regression were performed using the SCTransform function. To correct for batch effects, the Harmony algorithm^126^ was employed. Dimensionality reduction was achieved using principal component analysis (PCA), and the top 11 principal components (PCs) were utilized to construct a nearest-neighbors graph. Clustering was performed using the Louvain algorithm with a resolution parameter set to 0.8, allowing for the identification of distinct cell clusters.

#### Peripheral blood mononuclear cells

Processed single cell RNAseq data^43^ were downloaded from Array Express under accession number E-MTAB-10026 and subsequently loaded into Seurat version 3.2.2. Feature plots of *FTH1* and *FTL* expression across all cell types were made on UMAP embeddings using Seurat “FeaturePlot” function and subsequently the CD14+ Monocyte cell population across Status (both Healthy and Covid-19) was selected from the entire Seurat object. Violin plots of FTH1 and FTL expression across Status and Patient Status on Day of Collection were made using Seurat function VlnPlot and statistics were calculated using pairwise Wilcox tests with fdr correction applied.

### Human monocyte proteomics

Human healthy control subjects and subjects with ARDS were recruited and written informed consent obtained directly or by proxy under the ‘META-CYTE’ study (17/SS/0136/AM01) and ‘ARDS-NEUT’ study (20/SS/0002), as approved by the Scotland A Research Ethics Committee; see prior published study for recruitment detials^46^. Following red blood cell lysis, up to 10 million cells were stained for flow cytometry assessment and sorting, and sorted monocytes were processed for proteomic analysis as previously described^46^.

### Experimental animals and models

All murine experiments were approved by the WCM Institutional Animal Care and Use Committee (Protocol Number: 2023-0013) and were conducted in compliance with the 3Rs principles (Replacement, Reduction, and Refinement) to ensure ethical and responsible animal use and the ARRIVE guidelines.

#### Animals

*Fth1* floxed (*Fth1*^*fl/fl*I^) mice on a C57BL/6J background were kindly provided by the laboratory of Dr. Anupam Agarwal at the University of Alabama at Birmingham (originally generated by Dr. Lukas C. Kuhn, EPFL, Switzerland). They were crossed with *Lysz2*^*Cre*^ mice (also known as LysM-Cre, The Jackson Laboratory, Stock No. 004781) and *Itgax*^*Cre*^ (also known as *Cd11c*^*Cre*^, The Jackson Laboratory, Stock No. 008068) to generate constitutive *Fth1*^*ΔLysM*^ and *Fth1*^*ΔCd11c*^ mice. *Ncoa4* floxed mice (created and shared by Dr. Joseph Mancias, now deposited in the Jackson Laboratory Stock No. 033295) were crossed with *Cd11c*^*Cre*^ mice to generate constitutive *Ncoa4*^*ΔCd11c*^ mice. All animals were maintained in normal housing conditions unless otherwise stated, with 12 h dark/12 h light cycles and normal ambient temperature and humidity.

#### Models

In independent experiments, 7–10 week-old sex-matched wildtype C57BL/6J mice (The Jackson Laboratory, Stock No. 000664), *Fth1* knockout mice (*Fth1*^*ΔLysM*^ and *Fth1*^*ΔCd11c*^) and their respective *Fth1*^*fl/fl*^ littermates, and *Ncoa4* knockout mice and their *Ncoa4*^*fl/fl*^ littermates were placed in a custom-build plexiglass chamber, with normal access to food and water, and exposed to hyperoxia using high-flow oxygen, achieving an oxygen concentration >95% in the chamber. WT mice with and without treatment with water with 1 mg/mL deferiprone (Chiesi), with and without treatment with intraperitoneal 10 mg/kg liproxstatin-1 (Lip-1, SelleckChem) every other day for 7 days were similarly exposed to hyperoxia. Ferrostatin-1 (Fer-1, SellecChem) was administered at a dose of 1 mg/kg via intraperitoneal injection daily 1 week prior to hyperoxia exposure. *Fth1*^*ΔLysM*^ and littermate control mice were instilled intranasally with influenza (A/Puerto Rico/8/1934, H1N1) as a representative virus-induced lung injury model^127^, while *Ncoa4*^*ΔCd11c*^ mice and littermate control mice were instilled intranasally with *S. pneumoniae* (ATCC 6303) as a representative bacterial lung injury model^128^. In the BALF transplant experiments, bronchoalveolar lavage was performed was performed by slowly washing the airways of *Fth1*^*fl/fl*^ and *Fth1*^*ΔLysM*^ mice with PBS. BALF was then centrifuged to remove lavage cells, and 50 μL of the supernatant fluid was intratracheally instilled into anesthetized wildtype mice. Following an hour of observation for recovery, BALF-transplanted mice were exposed to hyperoxia. Both male and female mice were used for all experiments in balanced proportions.

#### Animal sample collection and analysis

Serum and plasma samples were collected following euthanasia via inferior vena caval access. BALF was collected as above and BALF supernatant was analyzed for total protein (Pierce BCA Protein Assay Kit, Thermo Fisher 23225), IgM (Thermo Fisher 88-50470-88), ferritin (Abcam ab157713), lactate dehydrogenase (LDH, Abcam ab102526), MCP-1 (Thermo Fisher 88-7391-22), MCP-3 (Abcam ab205571), and MCP-5 (Thermo Fisher EMCCL12), by ELISA. BALF pellet was resuspended, the total concentration of cells counted manually, and then subsequently a cytospin preparation was performed to allow differential counting of immune cells. BALF cell death was assayed using a TUNEL assay kit (Abcam ab206386) on cytospin slides. BALF cell 4-hydroxynonenal (4-HNE) immunocytochemistry was performed on cytospin slides (Abcam ab46545). Serum ferritin was similarly measured by ELISA, while serum hepcidin was assayed by ELISA (Intrinsic Lifesciences HMC-001). Plasma cytokines were assayed via a multiplex panel (Eve Technologies, Calgary, Alberta). All data points represent distinct biological samples unless otherwise specified.

For histology, lungs were slowly inflated with 4% paraformaldehyde (PFA) in PBS and maintained for 15 min, following which lungs were carefully extracted from the thoracic cavity. Fixed lungs were embedded in paraffin and sectioned in 5-µm-thick slices. Sections were dewaxed and rehydrated by incubation with xylene and descending ethanol concentrations and then stained with hematoxylin–eosin (H&E) for histological analysis. H&E-stained lung sections were evaluated and scored by a board-certified veterinary pathologist (S.E.C.) blinded to the genotype and treatment group using a semiquantitative histopathology scoring system used for mouse models of ARDS and SARS-CoV-2^129,130^. Briefly, six random fields of the lung lobe at ×200 to ×400 total magnification were chosen and scored for histopathological changes. Bronchiolar epithelial necrosis was assigned using the following tiers: 0, within expected limits; 1, uncommon, <5%; 2, detectable in 5–33%; 3, detectable in 34–66%; and 4, detectable in >66% of lung fields^130^ Lungs were graded for the presence of proteinaceous debris and fibrin, hyaline membranes, neutrophils in alveolar and interstitial spaces, alveolar epithelial necrosis, and macrophages in alveolar and perivascular/peribronchiolar spaces using histopathological scoring system for acute lung injury and diffuse alveolar damage^129^, with final scores obtained by averaging six fields per mouse. Sections from a subset of spleens were stained for iron using Perls’ Prussian blue reaction. An Olympus BX45 light microscope was used to capture images with a DP26 camera using cellSens Dimension software (v1.16).

#### Flow cytometry

For BALF cells, BALF was centrifuged at 450 × *g* for 5 min at 4 °C and cell pellet was obtained. For lung single cell suspensions, lungs were homogenized and digested with Collagenase A (2.5 mg/mL; Sigma-Aldrich) and Dispase II (1.0 mg/mL; Sigma-Aldrich) and the single cell suspension was filtered through a 40-μm filter^131^. Red blood cells (RBCs) in the cell pellet from lungs or BALF were lysed by incubating with RBC lysis buffer (1x; BioLegend #420302) for 3 min at 4 °C. Cells were washed twice with PBS (1x) at 450 × *g* for 5 min and stained with a viability dye (LIVE/DEAD™ Fixable Dead Cell Stain Kit Thermo Fisher Scientific or Zombie NIR™ Fixable Viability Kit Biolegend) for 30 min at 4 °C in the dark. After washing with PBX (1x), cells were incubated with anti-CD16 and anti-CD32 antibodies (clone 2.4G2 BioLegend) for 10 min at 4 °C to block Fc receptors. Surface staining was performed by incubating the cells with the following antibodies for 30 min at 4 °C: anti-CD45 (clone 30-F11 BD Biosciences), anti-Siglec-F (clone 1RNM44N eBioscience or clone E50-2440 BD Biosciences), anti-CD11c (clone HL3; BD Biosciences), anti-CD11b (clone M1/70; BioLegend), anti-Ly6C (clone HK1.4; BioLegend), and anti-Ly6G (clone 1A8; BioLegend). Cells were washed twice with PBS (1x) and stained with Liperfluo dye Liperfluo (Dojindo Laboratories) to detect lipid peroxides. Cells were washed again with PBS (1x) and stained with FerroOrange dye (Dojindo Laboratories) by following manufacturer’s instructions. To account for autofluorescence and other artifacts, fluorescence-minus-one (FMO) controls for both FerroOrange and Liperfluo were used to validate the quantitative differences in their signals (Supplementary Fig. 16A, B). Stained cells were acquired on a BD Symphony A5 flow cytometer (BD Biosciences). Data were analyzed using FlowJo v10.8 software (BD Biosciences).

#### RNA extraction and real-time polymerase chain reaction

Total RNA was extracted from BMDM or AM using Qiagen RNeasy Micro Kit and reverse transcribed into cDNA using a High Capacity cDNA Reverse Transcription kit (Life Technologies). Real-time PCR was performed using qPCR master mix (Life Technologies) on a ABC instrument with TaqMan gene expression primers for *Fth1* (Mm00850707_g1) and *Ftl* (Mm03030144_g1).

#### Western blots and antibodies

Cell lysates were generated from BMDM and BALF cells from room air and mice exposed to hyperoxia via sonication and RIPA Cell Lysis Buffer (ThermoFisher 89901). These lysates were then boiled at 100 °C for 5 min in loading buffer containing SDS and following resolution on 4–12% SDS-PAGE gels, transferred onto nitrocellulose membranes. Specific proteins blotted for include FTH1 (CST 3998), FTL (Abcam 109373), TFRC (Abcam 214039), SLC7A11 (CST 12691), GPX4 (Abcam 125066), and BACTIN (Sigma Aldrich A2228). Quantification analysis was performed using imagej software (https://imagej.net/ij/).

### Bone marrow-derived macrophage experiments

Bone marrow cells were isolated from the murine femur and tibia through a 25 G needle with a 12 ml syringe filled with complete DMEM attached (Life Technologies). Cell aggregates were removed using an 18 G needle and 70 µM cell strainer, after which the single cell suspension was centrifuged at 450 × *g* for 5 min. Cells were plated in complete DMEM supplemented with 10 ng/mL M-CSF for 7 days for in vitro experiments, for 3 days when followed by RNA isolation and RNA-Seq analysis.

#### In vitro experiments

BMDM were treated with 1 μM RSL3 (Selleckchem, Catalog No.S8155) for 4 h. FAC Viability was assessed using the AlamarBlue Cell Viability Reagent (Thermo Fisher DAL1025) or PrestoBlue Cell Viability Reagent (Thermo Fisher A13262) where indicated according to manufacturer protocol. In vitro hyperoxia was modeled using a Stemcell Hypoxia Chamber (Catalog# 27310) and oxygen with 5% CO_2_ mixed in, filled until achieving a chamber oxygen concentration of greater than 90%. The cells are then incubated for 24 h, with the oxygen sensor left in to ensure that oxygen levels do not fall during the incubation. BMDM were treated with 10 ng/mL LPS for 4 and 24 h, and IL-6 and TNF-α were measured in the media supernatant via ELISA (Thermo Fisher 88-7064-88 and 88-7324-88).

#### Bulk RNA-seq analysis

Sample files were checked for sequence quality using FastQC^132^. The resulting reads were mapped to the mouse reference genome GRCm38 using STAR^133^ aligner and gene-wise expression counts generated using the “-quantMode GeneCounts” parameter. After filtering for lowly expressed genes, the R package edgeR^134^ was used to perform differential gene expression comparisons and calculate FPKM values normalized by library size. Gene set enrichment analysis was performed using the Enrichr^135^ platform. Three selected libraries were queried through the Enrichr web API to identify overrepresented biological pathways in select gene lists. Sets of genes were determined as an FDR < 0.05 and a logFC > 0 for results for the condition group or logFC < 0 for results for the control group. Results were ranked by the Combined Score, which balances statistical significance (*p* value computed using the Fisher exact test) with the deviation (*z*-score) from the expected rank. The top enriched terms from each library were plotted based on the descending Combined Score, with manual removal of redundant terms.

### Ferritin isolation and characterization

For ferritin isolation, serum was buffered by adding 0.05 M sodium acetate in a 1-to-1 ratio, and then adding 1 M acetic acid to reach a target pH of 5. This buffered serum was subsequently heated at 70 °C for 10 min (9), and then centrifuged at 15,000 × *g* for 30 min. The resultant pellet of denatured protein was discarded, and protein from the supernatant was precipitated using cold acetone. Acetone at –20 °C was added to the heated serum supernatant at a 4-to-1 ratio by volume, and this mixture was incubated at −20 °C for 1 h. This was then centrifuged at 15,000 × *g* for 10 min, and the supernatant acetone was carefully removed by pipetting. The protein pellet was left to air dry for no more than 15 min to allow the residual acetone to evaporate, and then resuspended for native gel electrophoresis. Bands excised following standard Coomassie Blue staining protocol^136^ were submitted for mass spectrometry analysis.

### Total and ferrous iron quantification

Samples were prepared by adding a digestion buffer, 50% nitric acid by volume with 0.1% digitonin (Sigma-Aldrich D141) to the sample in a 1:1 ratio, then heated at 70 °C for 2 h in a heat block. The digested samples are then removed, cooled to room temperature, and centrifuged for 5 min at 6000 × *g*. The supernatant is collected and then diluted in dilute 0.2% nitric acid for measurement via the graphite furnace atomic absorption spectrometer (PerkinElmer model 900z). Iron is measured in the digestion buffer to assess iron contamination, and sample iron levels are quantified using a standard curve of known iron concentrations. For the ferrous iron studies, BMDM were seeded in a 96 well plate, and iron was added to the media via 100 µM FAC for 30 min and 4 h. The cells were then washed and incubated with FerroOrange (Dojindo F374) for 30 min and immediately read via a microplate reader.

### Mass spectrometry

Protein bands corresponding to SDS gel electrophoresis followed by Coomassie blue staining were excised from the gel. Excised gel bands were reduced with 5 mM dithiothreitol (DTT) and alkylated with 14 mM iodoacetamide, then digested in-gel with 12 ng/uL sequencing-grade trypsin (Promega) overnight at 37 °C. Digested peptides were vacuum-centrifuged to near dryness and desalted using micro-C18 columns prior to LC–MS/MS analysis.

Peptides were separated using an EASY-nLC 1000 system (Thermo Fisher Scientific) coupled online to a Fusion Lumos Orbitrap mass spectrometer (Thermo Fisher Scientific). Peptide separation was performed on 75 µm × 15 cm in-house-packed C18 columns (ReproSil-Pur C18-AQ, 3 µm, Dr. Maisch GmbH, Germany) at a flow rate of 300 nL/min. Peptides were eluted using a linear gradient of 3–30% buffer B over 50 min, followed by 30–80% buffer B over 10 min (buffer A: 0.1% formic acid in water; buffer B: 0.1% formic acid in acetonitrile).

The mass spectrometer was operated in data-dependent acquisition (DDA) mode. Full MS scans were acquired in the Orbitrap analyzer over a mass range of *m*/*z* 300–1500 at a resolution of 60,000. The top 15 most abundant precursor ions with charge states 2–5 were selected for fragmentation using a 1.4 m/z isolation window in the quadrupole and fragmented by higher-energy collisional dissociation (HCD) with a normalized collision energy of 35. MS/MS spectra were acquired in the Orbitrap analyzer at a resolution of 15,000. The automatic gain control (AGC) target values were set to 1 × 10⁶ for full MS scans and 5 × 10⁴ for MS/MS scans, with a maximum injection time of 60 ms for both.

Raw data files were processed using MaxQuant version 1.6.17.0 (Max Planck Institute, Munich, Germany). MS/MS spectra were searched against the UniProt human protein database (downloaded 09/21/2017) using the built-in Andromeda search engine. A reverse decoy database was used for false discovery rate (FDR) estimation. Trypsin was specified as the proteolytic enzyme with up to two missed cleavages allowed. Carbamidomethylation of cysteine residues was set as a fixed modification. Oxidation of methionine and protein N-terminal acetylation were specified as variable modifications. Precursor and fragment ion mass tolerances were set to 7 ppm and 20 ppm, respectively. Peptide- and protein-level identifications were filtered to a 1% FDR. Proteins were identified based on peptides uniquely mapping to the protein sequence using MaxQuant’s default protein inference rules. No additional post-identification filtering criteria were applied beyond those implemented in MaxQuant.

### Statistical analysis

#### Clinical cohort

Patient clinical characteristics were compared across strata defined by ferritin level, number of ferritin measurements, and clinical outcome. Patient strata were compared using Wilcoxon rank sum tests or Kruskal-Wallis rank sum tests for continuous variables, Pearson’s Chi-squared tests for categorical variables with all expected cell counts ≥5, and Fisher’s exact tests for categorical variables with one or more expected cell counts <5. Significance values for Fisher’s exact tests were simulated using Monte Carlo simulation when appropriate. The significance threshold for all null hypothesis tests was set at 0.05.

We compared patient- and cohort-level trajectories of ferritin measurements to progression of clinical characteristics during hospital stay. Patient ferritin trajectories were plotted on the log_10_ scale and smoothed using nonparametric penalized cubic regression splines, implemented with the R package *mgcv*^137^. When patients were stratified by clinical characteristic, smoothing was performed within defined strata. In ARDS cases prior to the COVID-19 pandemic, the closest ferritin measurement within ±3 days of ICU admission was collected for each ICU course. Ferritin values in ng/mL were log-transformed and compared across ARDS and non-ARDS patients using generalized estimating equations (GEE) models, which accounted for repeated measurements within the same patient but across multiple admissions. In visualizations, trajectories were truncated at 80 days post-admission due to sparsity of ferritin data beyond that point. We also derived three ferritin measurements specific to our cohort to facilitate comparison of ferritin levels across patients. Baseline ferritin was defined as a subject’s first ferritin measurement within 21 days of admission; maximum ferritin was defined as a subject’s highest ferritin measurement during their entire admission; and delta ferritin was defined as the difference between a subject’s first and last ferritin measurements within 21 days of admission. Distributions of derived ferritin measurements, accounting for data missingness, were compared across patient strata using violin plots, with stratum-specific medians indicated and compared using Kruskal-Wallis rank sum tests. A multivariable logistic regression model was used to estimate the adjusted association between delta ferritin and 28-day mortality, with a 95% confidence interval estimated for the adjusted odds ratio. This model was adjusted for the following potential confounders of the exposure-outcome relationship: age, sex, hypertension, coronary artery disease, diabetes, active cancer, and BMI. To aid in interpretability, model results were reported per 100 ng/mL increase in delta ferritin. All analyses were performed using R version 4.0.2^138^. Plots were created using the R package *ggplot2*^139^, and tables were constructed using the R package *gtsummary*^140^.

#### Murine and in vitro studies

Statistical analyses for murine and in vitro experimental data are detailed in the corresponding figure legends. Unless otherwise specified, data are presented as mean ± standard error of the mean (SEM). All Ns represent biological replicates unless otherwise indicates. Data distribution was assessed prior to statistical testing, and parametric tests were used where assumptions of normality and variance were met. For comparisons between two groups, unpaired two‑tailed Student’s *t* tests were used. For experiments involving multiple groups or multiple conditions, two‑way analysis of variance (ANOVA) with Šídák’s correction for multiple comparisons was applied, as appropriate. For datasets that did not meet assumptions for parametric testing, non‑parametric tests were used as indicated in the figure legends. Survival analyses were performed using the Gehan–Breslow–Wilcoxon test. No data were excluded from analysis unless pre‑specified by experimental design. Sample sizes and the number of biological replicates are described in the corresponding figure legends. Statistical significance was defined as a two‑sided *p* value < 0.05. All analyses were performed using GraphPad Prism unless otherwise stated.

### Reporting summary

Further information on research design is available in the Nature Portfolio Reporting Summary linked to this article.

## Supplementary information

Supplementary Information

Reporting Summary

Transparent Peer Review file

## Source data

Source data

## Data Availability

Lung single-cell data are available via the Columbia University/NYP COVID-19 Lung Atlas using single-cell portal: https://singlecell.broadinstitute.org/single_cell/study/SCP1219; the data were previously also deposited in GEO with accession number GSE171524. The peripheral blood single cell data can be explored through the portal https://covid19cellatlas.org/; processed data can be downloaded from Array Express under accession number E-MTAB-10026. For the Weill Cornell Medicine cohort, all raw sequence files (FASTQs) and metadata for specimens, including per-run metrics and QC data, have been submitted to the database of Genotypes and Phenotypes dbGAP (accession #38851 and ID phs002258.v1.p1). The mass spectrometry proteomics data from murine experiments have been deposited to the ProteomeXchange Consortium via the PRIDE partner repository with the dataset identifier PXD079195^141^. Other supporting data for all values underlying data presented in graphs, as well as western blot data, are provided in the Supplementary Information or in the source data. Source data are provided with this paper.
