# Supplementary material for: Targeting macrophage ferritin heavy chain mitigates ferroptosis and lung injury in experimental acute respiratory distress syndrome
Source: Nat Commun. 2026 Jun 27;17:8057. doi: 10.1038/s41467-026-74828-w (PMC13454589; doi:10.1038/s41467-026-74828-w)
Supplement: Supplementary file 1 [file 41467_2026_74828_MOESM1_ESM.pdf]

Supplementary Table 1. Baseline Characteristics of NYP COVID-19 Cohort by Outcome.

Parameter	Intubation/Death (n=157)	Intubation/Discharge (n=249)	No Intubation/Discharge (n=965)	p-value
Sex n (%)				<0.001
Female	42 (27%)	83 (33%)	413 (43%)	
Male	115 (73%)	166 (67%)	552 (57%)	
Age [IQI]	71 [63, 78]	61 [50, 70]	61 [49, 71]	<0.001
Race n (%)				<0.001
Asian	41 (26%)	45 (18%)	144 (15%)	
Black	14 (8.9%)	20 (8.0%)	157 (16%)	
Hispanic	37 (24%)	72 (29%)	230 (24%)	
White	44 (28%)	61 (24%)	257 (27%)	
BMI [IQI]	27 [23, 31]	29 [26,33]	27 [24,31]	<0.001
Hypertension n (%)	100 (64%)	135 (54%)	490 (51%)	0.010
Diabetes n (%)	59 (38%)	83 (33%)	279 (29%)	0.056
Coronary Artery Disease n (%)	42 (27%)	29 (12%)	110 (11%)	<0.001
COPD n (%)	15 (9.6%)	13 (5.2 %)	25 (2.6%)	<0.001
Active Cancer n (%)	14 (8.9%)	8 (3.2%)	55 (5.7%)	0.051
Supplemental Oxygen in 1st 3 Hours n (%)	135 (86%)	195 (78%)	390 (40%)	<0.001

Data are presented as n (%) or median (interquartile range). P-values represent global comparisons across all three groups. Continuous variables were compared using Kruskal-Wallis rank-sum tests and categorical variables using Pearson’s chi-squared tests. All tests were two-sided. P-values were not adjusted for multiple comparisons.

SUPPLEMENTARY FIGURE LEGENDS

Supplementary Figure 1. Ferritin parameters in patients with COVID-19 ARDS. (**A**) Flow diagram of the NYP COVID-19 Cohort. (**B**) Schematic illustrating the calculation of delta ferritin in subjects in the NYP COVID-19 cohort (n=343), with red circles representing first serum ferritin measurement available during hospital admission and green circles last serum ferritin measurement prior to 21 days admission, death, or hospital discharge, whichever is later. (**C**) Serum ferritin within 3 days of ICU admission in ICU patients with (n=477) and without ARDS (n=3317), with p-value by Kruskal-Wallis test.

Supplementary Figure 2. Monocyte *FTL* expression across COVID-19 severity. (**A**) Violin plots of CD14+ Monocytes of *FTL* expression by donor cells across increasing covid severity. P-values calculated by Kruskal-Wallis test. ****$p<0.0001$. (**B**) Peripheral blood mononuclear cell (PBMC) expression of *FTH1* in the NYP COVID-19 cohort, displayed as violin plots with solid line indicating the median expression and dashed lines indicating the upper and lower quartiles, with p-value by unpaired Student's t-test. Classical monocyte FTH1 (**C**) and FTL (**D**) quantified via proteomics in subjects without (n=3) and with (n=4) ARDS.

Supplementary Figure 3. ARDS alters lung macrophage and extracellular ferritin expression. (**A**) BALF ferritin levels in control mice instilled with PBS and 72 hours following S. pneumoniae instillation. Real-time polymerase chain reaction (RT-PCR) of unsorted BALF cell *Fth1* (**B**, p=0.027) and *Ftl* (**C**, p=0.0041). (**D**) *Fth1* Transcripts per Million (TPM) from bulk RNA-Seq of BAL cells following intratracheal LPS instillation. Data presented as mean ± SEM with p-value by unpaired Student's t-test, *$p<0.05$, **$p<0.01$, ****$P<0.0001$.

Supplementary Figure 4. Hyperoxia exposure results in BMDM differential gene expression. (**A**) Schematic of BMDM RNA-Seq experiment. Created in BioRender. Cloonan, S. (2026)

https://BioRender.com/7e3d0fh (**B**) Gene ontology (biological process and molecular function) and Kyoto Encyclopedia of Genes and Genomes (KEGG) analysis of upregulated genes meeting the FDR cut-off in BMDM from mice exposed to hyperoxia relative to those from RA mice, illustrated using Enrichr.

Supplementary Figure 5. Murine LysM-Cre targeted FTH1 deletion. (A) Breeding scheme for myeloid FTH1-deficient (*Fth1*$^{\Delta LysM}$) mice. Created in BioRender. Cloonan, S. (2026) https://BioRender.com/zmynjmh. Real-time polymerase chain reaction (RT-PCR) of BMDM Fth1 (**B**, n=3 for *Fth1*$^{fl/fl}$, n=3 for *Fth1*$^{\Delta LysM}$) and AM Fth1 (**C**, n=2 for *Fth1*$^{fl/fl}$, n=3 for *Fth1*$^{\Delta LysM}$, p=0.0013). Data (B-C) presented as mean ± SEM with p-value by unpaired Student's t-test, *p<0.05, **p<0.01, ****P<0.0001. Representative immunoblot of FTH1 protein expression in AMs (**D**, n=2 for each group) BMDMs from *Fth1*$^{fl/fl}$ and *Fth1*$^{\Delta LysM}$ (**E**, n=3 for each group) mice. Dashed lines in D indicate where the image was cut and stitched from lanes from a single membrane. Repeated twice with similar results. (**F**) Representative immunoblot of FTH1 protein expression in lung immune cells ($CD45^+$) and epithelial cells ($CD45^-EpCAM^+$) from *Fth1*$^{fl/fl}$ (n=2) and *Fth1*$^{\Delta LysM}$ (n=2) mice, experiment performed one time. Levels of IL-6 (**G**, p=0.0010 between *Fth1*$^{fl/fl}$ and *Fth1*$^{\Delta LysM}$ at 24hr) and TNF-α (**H**, p=0.0002 between *Fth1*$^{fl/fl}$ and *Fth1*$^{\Delta LysM}$ at 4hr, p=0.034 at 24hr) at 4 hours and 24 hours in the supernatant of *Fth1*$^{fl/fl}$ (n=3) and *Fth1*$^{\Delta LysM}$ (n=3) BMDMs following 10 ng/mL LPS treatment in vitro. Data (G-H) presented as mean ± SEM with p-values 2-way analysis of variance (ANOVA) with Šídák's correction for multiple comparisons, *p<0.05, ***p<0.001.

Supplementary Figure 6. *Fth1*$^{\Delta LysM}$ mice are protected from influenza-induced lung injury. (**A**) Percent weight loss on day 7 relative to day 0 weight in *Fth1*$^{fl/fl}$ (n=14) and *Fth1*$^{\Delta LysM}$ (n=12) following intranasal influenza instillation. (**B**) BALF protein in PBS-instilled mice (n=5 for *Fth1*$^{fl/fl}$, n=2 for *Fth1*$^{\Delta LysM}$) and mice 7 days following influenza instillation (n=14 for *Fth1*$^{fl/fl}$, n=13 for

Fth1$^{\Delta LysM}$). Data presented as mean ± SEM with p-values by unpaired Student's t-test (A) and 2-way analysis of variance (ANOVA) with Šídák's correction for multiple comparisons (B).

Supplementary Figure 7. Cd11c-Cre driven FTH1 deletion protects against hyperoxia. (**A**) BALF total cell count and (**B**) macrophages and monocytes in *Fth1*$^{fl/fl}$ and *Fth1*$^{\Delta Cd11c}$ mice in room air and hyperoxia conditions. p=0.0017 in *Fth1*$^{\Delta Cd11c}$ mice RAv72hr, p=0.0003 between *Fth1*$^{fl/fl}$ and *Fth1*$^{\Delta Cd11c}$ at 96hr. p-values by 2-way analysis of variance (ANOVA) with Šídák's correction for multiple comparisons. **p<0.01, ***p<0.001, ****p<0.0001.

Supplementary Figure 8. Decreased BAL immune cells in *Fth1*$^{fl/fl}$ mice are not due to decreased chemokine signaling. BALF MCP-3 (**A**, n=3/7/6 for *Fth1*$^{fl/fl}$ mice and n=3/7/7 for *Fth1*$^{\Delta LysM}$ mice at the room air (RA)/72hr/96hr time points, p=0.0008 between *Fth1*$^{fl/fl}$ and *Fth1*$^{\Delta LysM}$ at 96hr) and MCP-5 (**B**, n=4/8/10 for *Fth1*$^{fl/fl}$ mice and n=5/6/7 for *Fth1*$^{\Delta LysM}$ mice at the RA/72hr/96hr time points, p=0.012 between *Fth1*$^{fl/fl}$ and *Fth1*$^{\Delta LysM}$ at 96hr), presented as mean ± SEM with p-values by 2-way analysis of variance (ANOVA) with Šídák's correction for multiple comparisons. *p<0.05, ***p<0.001.

Supplementary Figure 9. In vivo hyperoxia increases AM cell death and neutrophil recruitment. (**A**) Gating strategy for evaluation of cell death via flow cytometry. Proportions (**B**) and counts (**C**) of Zombie NIR- live neutrophils via gating strategy shown in (A). p-values by 2-way analysis of variance (ANOVA) with Šídák's correction for multiple comparisons.

Supplementary Figure 10. Liproxstatin-1 and ferrostatin-1 do not protect against HALI. Percent survival of mice (**A**) and percent weight loss at 96 hours (**B**) in mice treated with intraperitoneal DMSO (n=10) or liproxstatin-1 (Lip-1, n=10) following hyperoxia exposure. Percent survival of mice (**C**) and percent weight loss at 96 hours (**D**) in mice treated with intraperitoneal

DMSO (n=3) or ferrostatin-1 (Fer-1, n=4) following hyperoxia exposure. Survival analysis p-values (A, C) by Gehan-Breslow-Wilcoxon test and group comparison p-values (B, D) by unpaired Student's t-test.

Supplementary Figure 11. Serum hepcidin is elevated in hyperoxia. Serum (**A**, n=4/4/2 for *Fth1*$^{fl/fl}$ and n=4/6/4 for *Fth1*$^{\Delta LysM}$ mice at RA/72hr/96hr hyperoxia conditions) and BALF (**B**, n=3/4 for *Fth1*$^{fl/fl}$ and *Fth1*$^{\Delta LysM}$ mice in RA/72hr hyperoxia conditions) hepcidin in *Fth1*$^{fl/fl}$ and *Fth1*$^{\Delta LysM}$ mice in room and following 72 and 96 hours of hyperoxia exposure. Data (A-B) presented mean ± SEM.

Supplementary Figure 12. Deferiprone lowers macrophage total iron levels but does not cause systemic anemia. (A) Total iron as measured by graphite furnace atomic absorption spectrometry (GFAAS) in fetal liver-derived macrophages (FLAMs) treated with 24 hours of 100 μM of deferiprone (DFP) relative to control macrophages. (**B**) Plasma hemoglobin levels in mice treated with 7 days of control water or water with 1 mg/mL deferiprone. Data (A-B) presented mean ± SEM with p-value by unpaired Student's t test, ***p<0.001.

Supplementary Figure 13. Alveolar macrophage (AM) NCOA4 depletion alters AM iron metabolism and cellular response to hyperoxia. Real-time polymerase chain reaction (RT-PCR) of AM *Ncoa4* (**A**, n=10 for *Ncoa4*$^{fl/fl}$, n=7 for *Ncoa4*$^{\Delta Cd11c}$), *Fth1* (**B**, n=8 for *Ncoa4*$^{fl/fl}$, n=5 for *Ncoa4*$^{\Delta Cd11c}$) and *Tfrc* (**C**, n=8 for *Ncoa4*$^{fl/fl}$, n=8 for *Ncoa4*$^{\Delta Cd11c}$, p=0.0019) mRNA expression. (**D**) Total iron content in AMs from *Ncoa4*$^{fl/fl}$ and *Ncoa4*$^{\Delta Cd11c}$ mice, normalized to total protein. BALF total cell count (**E**, n=3/6 for both *Ncoa4*$^{fl/fl}$ and *Ncoa4*$^{\Delta Cd11c}$ in RA/hyperoxia conditions), macrophages/monocytes (**F,** n=3/5 for both *Ncoa4*$^{fl/fl}$ and *Ncoa4*$^{\Delta Cd11c}$ in RA/hyperoxia conditions), and total iron by GFAAS in (**G,** n=3/7 for both *Ncoa4*$^{fl/fl}$ and *Ncoa4*$^{\Delta Cd11c}$ in RA/hyperoxia conditions) in RA and hyperoxia-exposed *Ncoa4*$^{fl/fl}$ and *Ncoa4*$^{\Delta Cd11c}$ mice. (**H**) Representative immunoblot of

NCOA4 protein expression in bone marrow-derived macrophages (BMDMs) from $Fth1^{fl/fl}$ (n=3) and $Fth1^{\Delta LysM}$ (n=3) mice; same membrane re-blotted as **Figure 3B**. Experiment repeated two times with similar results. Data (A-G) presented mean ± SEM with p-values by unpaired Student's t-test (A-D) or 2-way analysis of variance (ANOVA) with Šídák's correction (E-G) for multiple comparisons. **p<0.01, ****P<0.0001.

Supplementary Figure 14. Hyperferritinemia in $Fth1^{\Delta LysM}$ mice and following hyperoxia exposure is not associated with a macrophage activation syndrome. Plasma leukocyte (**A**) and hemoglobin (**B**) in $Fth1^{fl/fl}$ and $Fth1^{\Delta LysM}$ mice in RA and hyperoxia conditions (n=5/8/6 for $Fth1^{fl/fl}$ mice and n=7/10/8 for $Fth1^{\Delta LysM}$ mice in RA/72hr/96hr). (**C**) Representative images of spleen from $Fth1^{fl/fl}$ and $Fth1^{\Delta LysM}$ mice following 72 hours of hyperoxia exposure. (**D**) Heatmap of plasma cytokines from $Fth1^{fl/fl}$ and $Fth1^{\Delta LysM}$ mice following 72 hours of hyperoxia exposure.

Supplementary Figure 15. Serum ferritin in human ARDS is enriched for FTL. (**A**) Representative native electrophoresis gel image from native serum ferritin isolation, with quantification of FTL and FTH1 by mass spectrometry. Experiment repeated three times with similar results. (**B**) Relative percentage of FTL or FTH1 subunits. (**C**) Log total plasma iron displayed with log serum ferritin in matching subject.

Supplementary Figure 16. Fluorescence-minus-one (FMO) control validation of FerroOrange and Liperfluo. FerroOrange (**A**) and Liperfluo (**B**) dye signal in lung CD11c+ Siglec-Ft AMs, in FMO and full-stained samples.

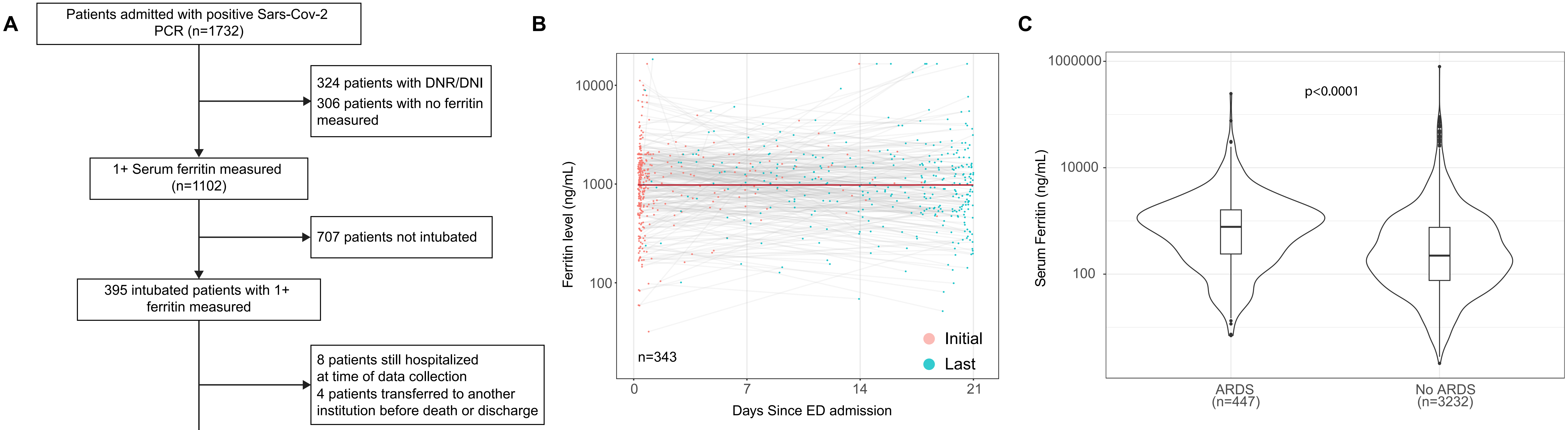

Supplementary Figure 1

Supplementary Figure 1. Ferritin parameters in patients with COVID-19 ARDS. (**A**) Flow diagram of the NYP COVID-19 Cohort. (**B**) Schematic illustrating the calculation of delta ferritin in subjects in the NYP COVID-19 cohort (n=343), with red circles representing first serum ferritin measurement available during hospital admission and green circles last serum ferritin measurement prior to 21 days admission, death, or hospital discharge, whichever is later. (**C**) Serum ferritin within 3 days of ICU admission in ICU patients with (n=477) and without ARDS (n=3317), with p-value by Kruskal-Wallis test.

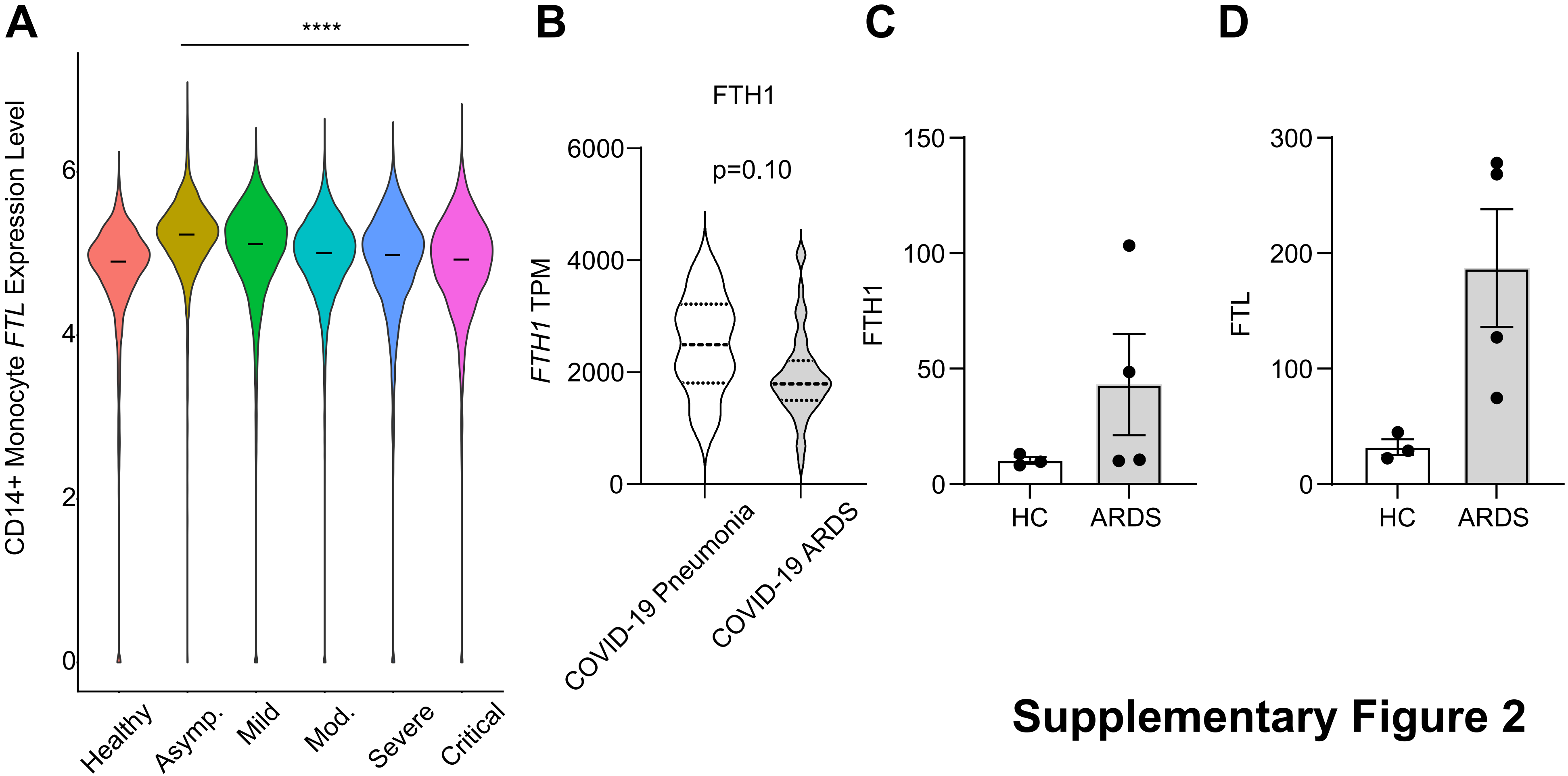

Supplementary Figure 2

Supplementary Figure 2. Monocyte FTL expression across COVID-19 severity. (**A**) Violin plots of CD14⁺ Monocytes of FTL expression by donor cells across increasing covid severity. P-values calculated by Kruskal-Wallis test. ****p<0.0001. (**B**) Peripheral blood mononuclear cell (PBMC) expression of *FTH1* in the NYP COVID-19 cohort, displayed as violin plots with solid line indicating the median expression and dashed lines indicating the upper and lower quartiles, with p-value by unpaired Student's t-test. Classical monocyte FTH1 (**C**) and FTL (**D**) quantified via proteomics in subjects without (n=3) and with (n=4) ARDS.

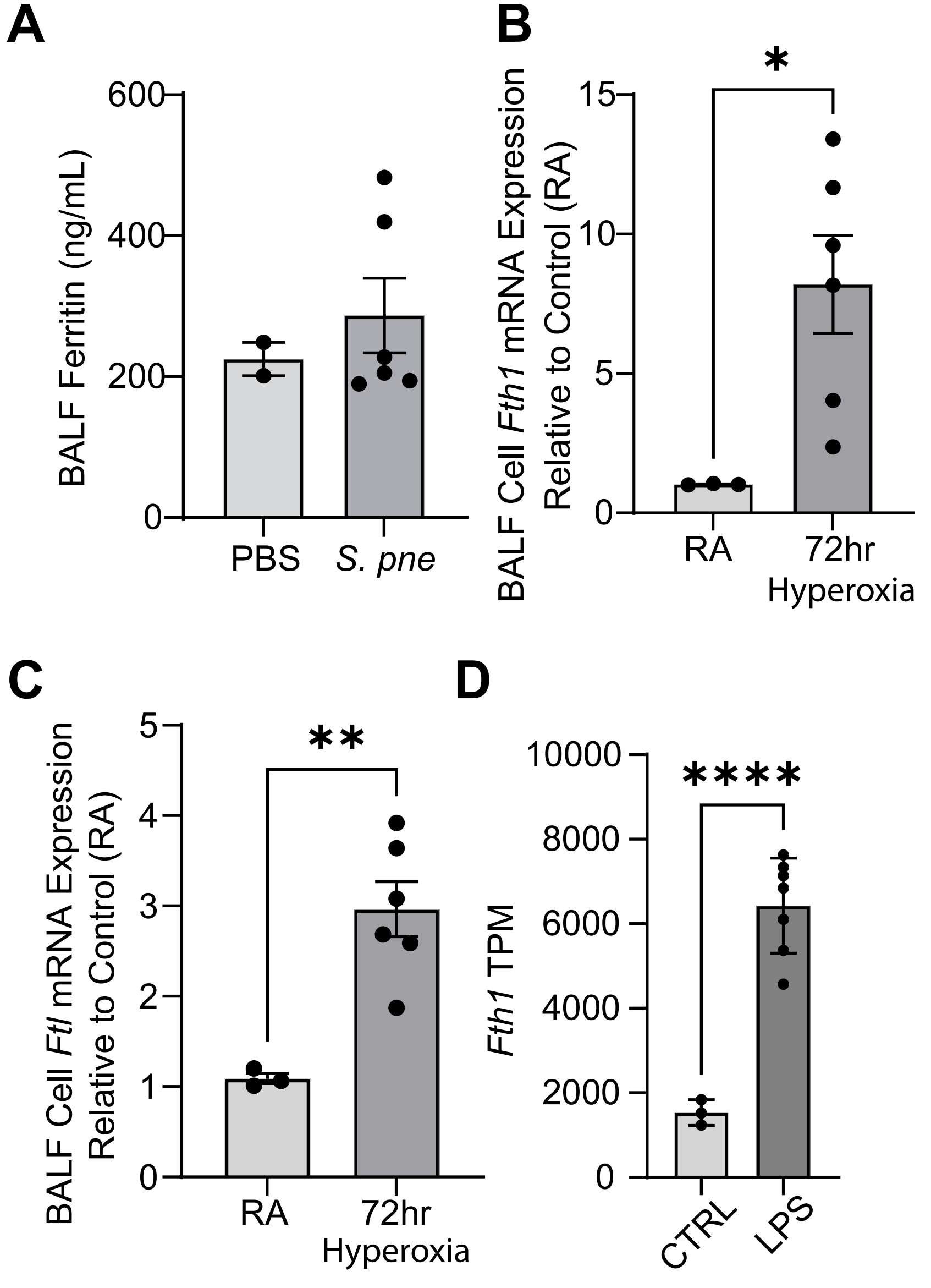

Supplementary Figure 3

Supplementary Figure 3. ARDS alters lung macrophage and extracellular ferritin expression. (**A**) BALF ferritin levels in control mice instilled with PBS and 72 hours following *S.* pneumoniae instillation. Real-time polymerase chain reaction (RT-PCR) of unsorted BALF cell *Fth1* (**B**, p=0.027) and *Ftl* (**C**, p=0.0041). (**D**) *Fth1* Transcripts per Million (TPM) from bulk RNA-Seq of BAL cells following intratracheal LPS instillation. Data presented as mean ± SEM with p-value by unpaired Student’s t-test, *p<0.05, **p<0.01, ****P<0.0001.

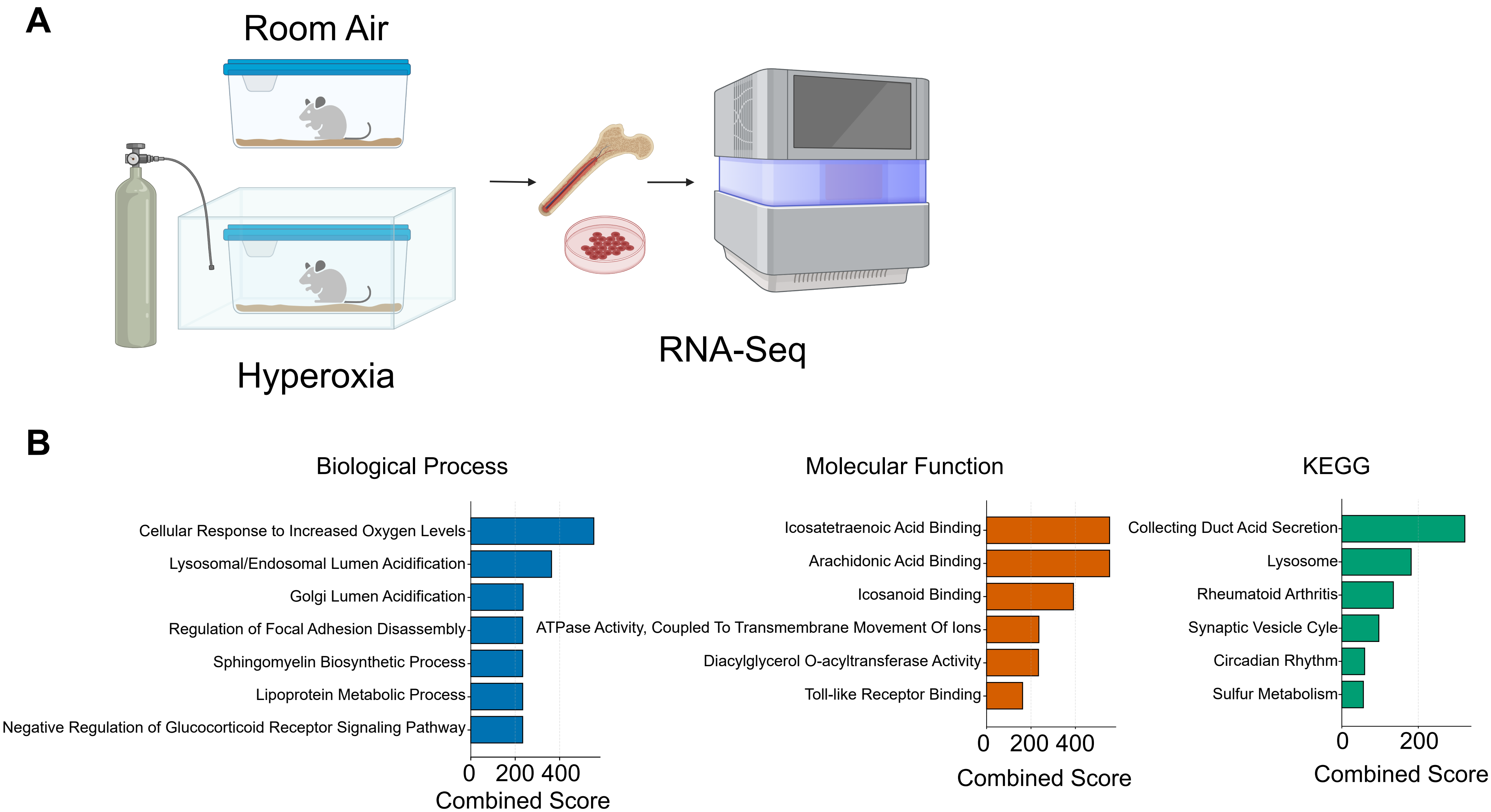

Supplementary Figure 4

Supplementary Figure 4. Hyperoxia exposure results in BMDM differential gene expression. (**A**) Schematic of BMDM RNA-Seq experiment. Created in BioRender. Cloonan, S. (2026) https://BioRender.com/7e3d0fh. (**B**) Gene ontology (biological process and molecular function) and Kyoto Encyclopedia of Genes and Genomes (KEGG) analysis of upregulated genes meeting the FDR cut-off in BMDM from mice exposed to hyperoxia relative to those from RA mice, illustrated using Enrichr.

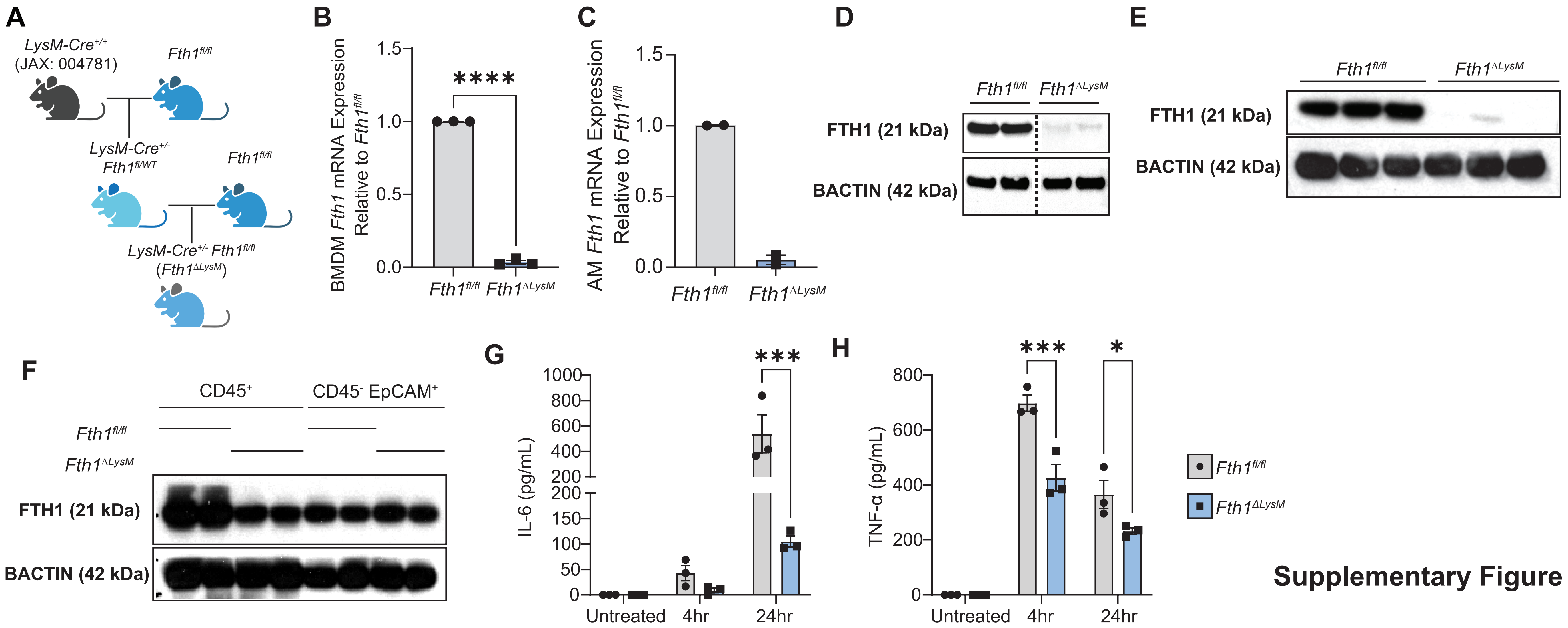

Supplementary Figure 5

Supplementary Figure 5. Murine LysM-Cre targeted FTH1 deletion. (A) Breeding scheme for myeloid FTH1-deficient (*$Fth1^{\Delta LysM}$*) mice. Created in BioRender. Cloonan, S. (2026) https://BioRender.com/zmynjmh. Real-time polymerase chain reaction (RT-PCR) of BMDM Fth1 (B, n=3 for *$Fth1^{fl/fl}$*, n=3 for *$Fth1^{\Delta LysM}$*) and AM Fth1 (C, n=2 for *$Fth1^{fl/fl}$*, n=3 for *$Fth1^{\Delta LysM}$*). Data (B-C) presented as mean ± SEM with p-value by unpaired Student's t-test, ****$p<0.0001$. Representative immunoblot of FTH1 protein expression in AMs (D, n=2 for each group) BMDMs from *$Fth1^{fl/fl}$* and *$Fth1^{\Delta LysM}$* (E, n=3 for each group) mice. Dashed lines in D indicate where the image was cut and stitched from lanes from a single membrane. Repeated twice with similar results. (F) Representative immunoblot of FTH1 protein expression in lung immune cells (CD45+) and epithelial cells (CD45-EpCAM+) from *$Fth1^{fl/fl}$* (n=2) and *$Fth1^{\Delta LysM}$* (n=2) mice, experiment performed one time. Levels of IL-6 (G, p=0.0010 between *$Fth1^{fl/fl}$* and *$Fth1^{\Delta LysM}$* at 24hr) and TNF-α (H, p=0.0002 between *$Fth1^{fl/fl}$* and *$Fth1^{\Delta LysM}$*, p=0.034 at 24hr) at 4 hours and 24 hours in the supernatant of *$Fth1^{fl/fl}$* (n=3) and *$Fth1^{\Delta LysM}$* (n=3) BMDMs following 10 ng/mL LPS treatment in vitro. Data (G-H) presented as mean ± SEM with p-values by 2-way analysis of variance (ANOVA) with Šídák's correction for multiple compari-

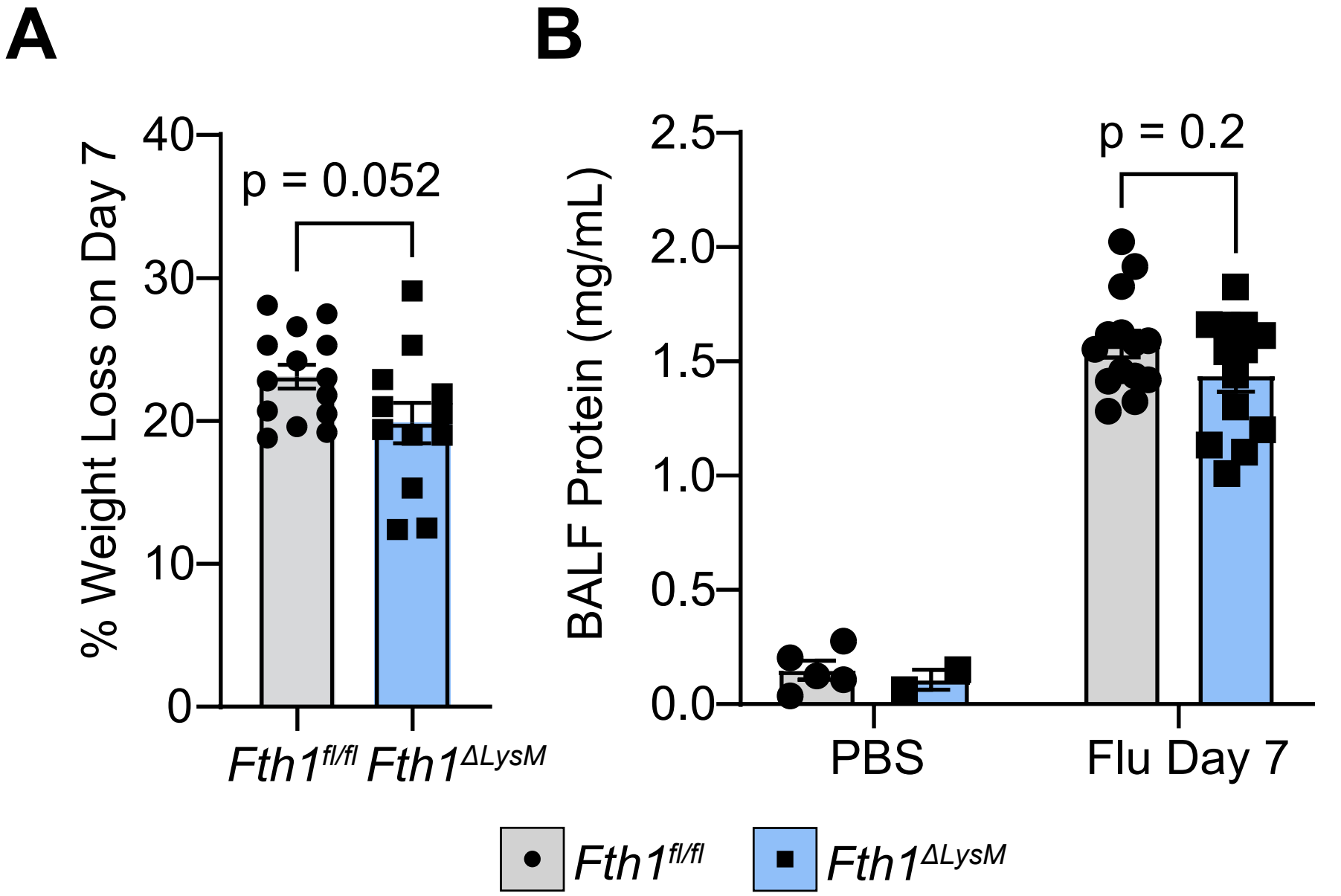

Supplementary Figure 6

Supplementary Figure 6. *Fth1$^{\Delta LysM}$* mice are protected from influenza-induced lung injury. (**A**) Percent weight loss on day 7 relative to day 0 weight in *Fth1$^{fl/fl}$* (n=14) and *Fth1$^{\Delta LysM}$* (n=12) following intranasal influenza instillation. (**B**) BALF protein in PBS-instilled mice (n=5 for *Fth1$^{fl/fl}$*, n=2 for *Fth1$^{\Delta LysM}$*) and mice 7 days following influenza instillation (n=14 for *Fth1$^{fl/fl}$*, n=13 for *Fth1$^{\Delta LysM}$*). Data presented as mean ± SEM with p-values by unpaired Student's t-test (A) and 2-way analysis of variance (ANOVA) with Šídák's correction for multiple comparisons (B).

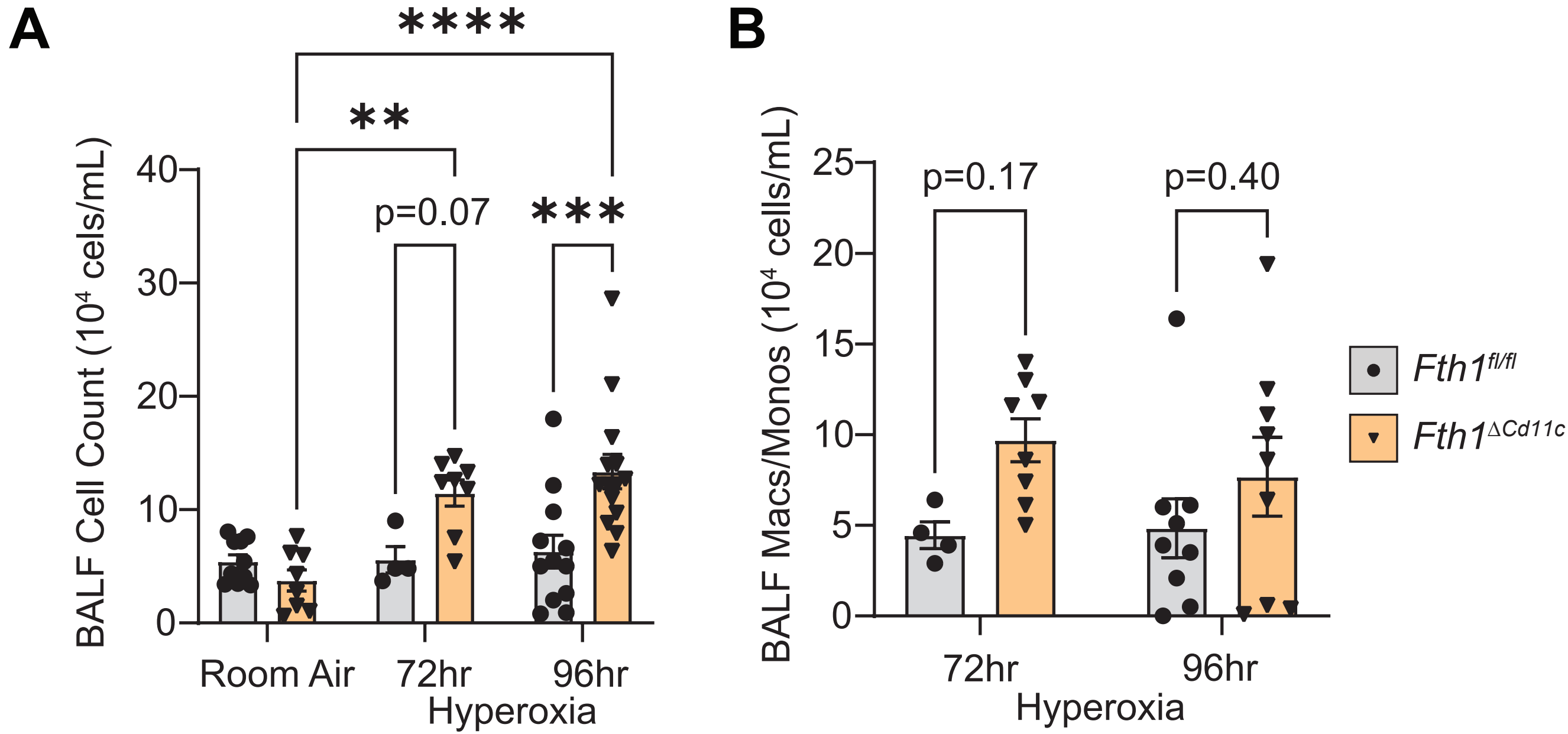

Supplementary Figure 7

Supplementary Figure 7. Cd11c-Cre driven FTH1 deletion protects against hyperoxia. (**A**) BALF total cell count and (**B**) macrophages and monocytes in *Fth1$^{fl/fl}$* and *Fth1$^{\Delta Cd11c}$* mice in room air and hyperoxia conditions. p=0.0017 in *Fth1$^{\Delta Cd11c}$* mice RAv72hr, p=0.0003 between *Fth1$^{fl/fl}$* and *Fth1$^{\Delta Cd11c}$* at 96hr. P-values by 2-way analysis of variance (ANOVA) with Šídák's correction for multiple comparisons. **p<0.01, ***p<0.001, ****p<0.0001.

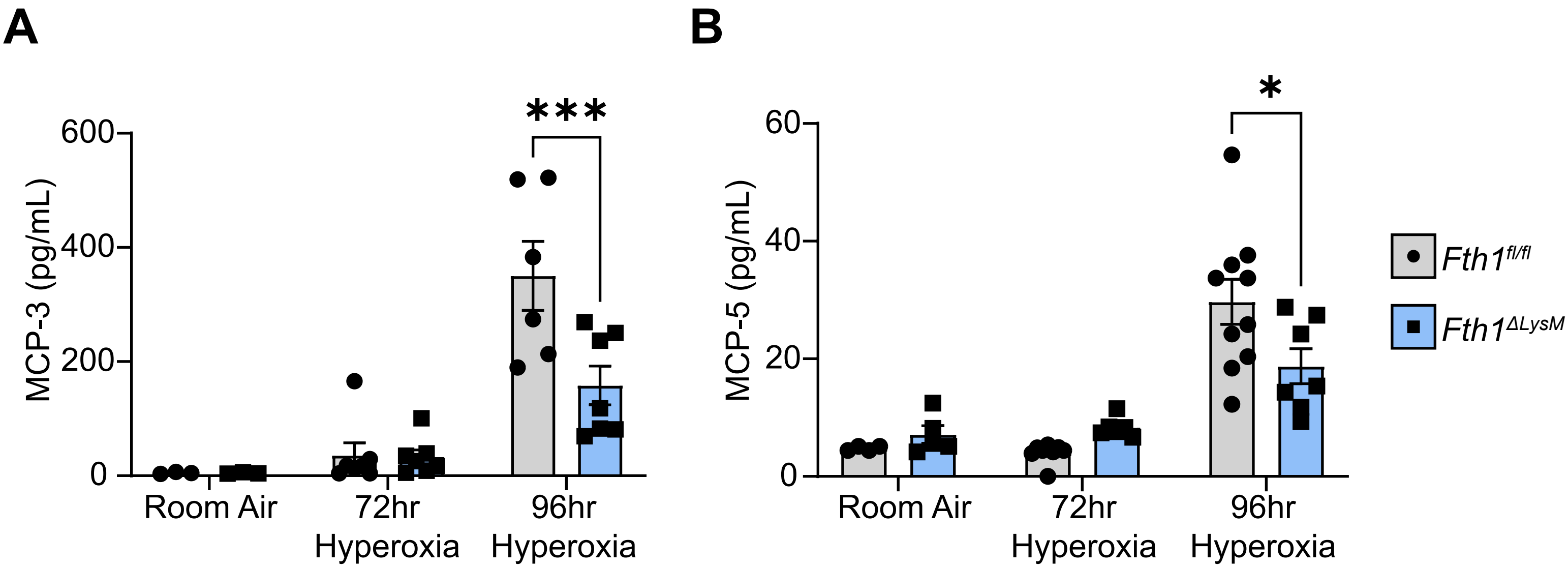

Supplementary Figure 8

Supplementary Figure 8. Decreased BAL immune cells in *Fth1^{fl/fl}* mice are not due to decreased chemokine signaling. BALF MCP-3 (**A**, n=3/7/6 for *Fth1^{fl/fl}* mice and n=3/7/7 for *Fth1^{ΔLysM}* mice at the room air (RA)/72hr/96hr time points, p=0.0008 between *Fth1^{fl/fl}* and *Fth1^{ΔLysM}* mice at 96hr) and MCP-5 (**B**, n=4/8/10 for *Fth1^{fl/fl}* mice atnd n=5/6/7 for *Fth1^{ΔLysM}* mice at the RA/72hr/96hr time points, p=0.012 between *Fth1^{fl/fl}* and *Fth1^{ΔLysM}* mice at 96hr), presented as mean ± SEM with p-values by 2-way analysis of variance (ANOVA) with Šídák's correction for multiple comparisons. *p<0.05, ***, p<0.001.

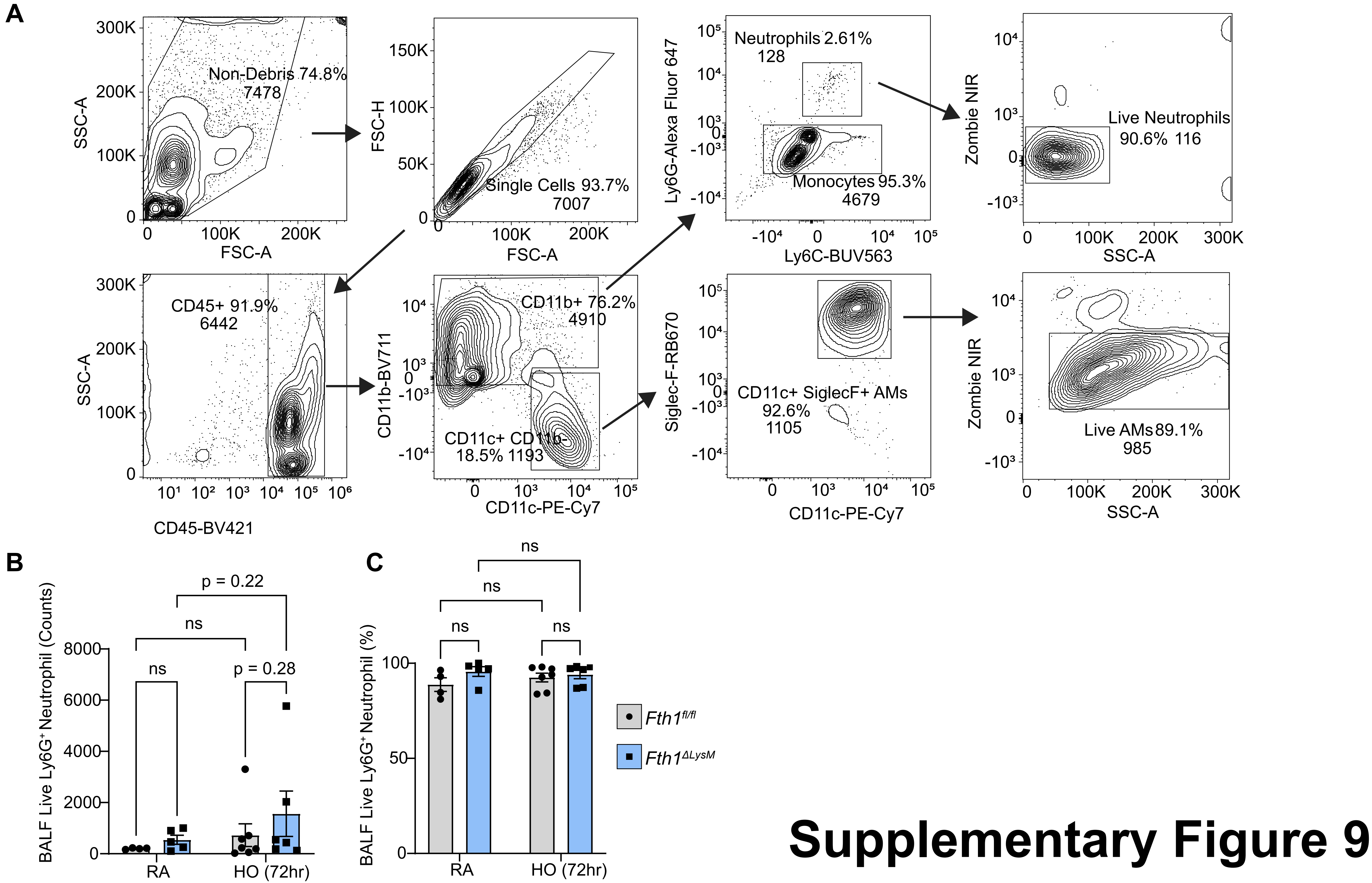

Supplementary Figure 9

Supplementary Figure 9. In vivo hyperoxia increases AM cell death and neutrophil recruitment. (**A**) Gating strategy for evaluation of cell death via flow cytometry. Proportions (**B**) and counts (**C**) of Zombie NIR- live neutrophils via gating strategy shown in (A). p-values by 2-way analysis of variance (ANOVA) with Šídák's correction for multiple comparisons.

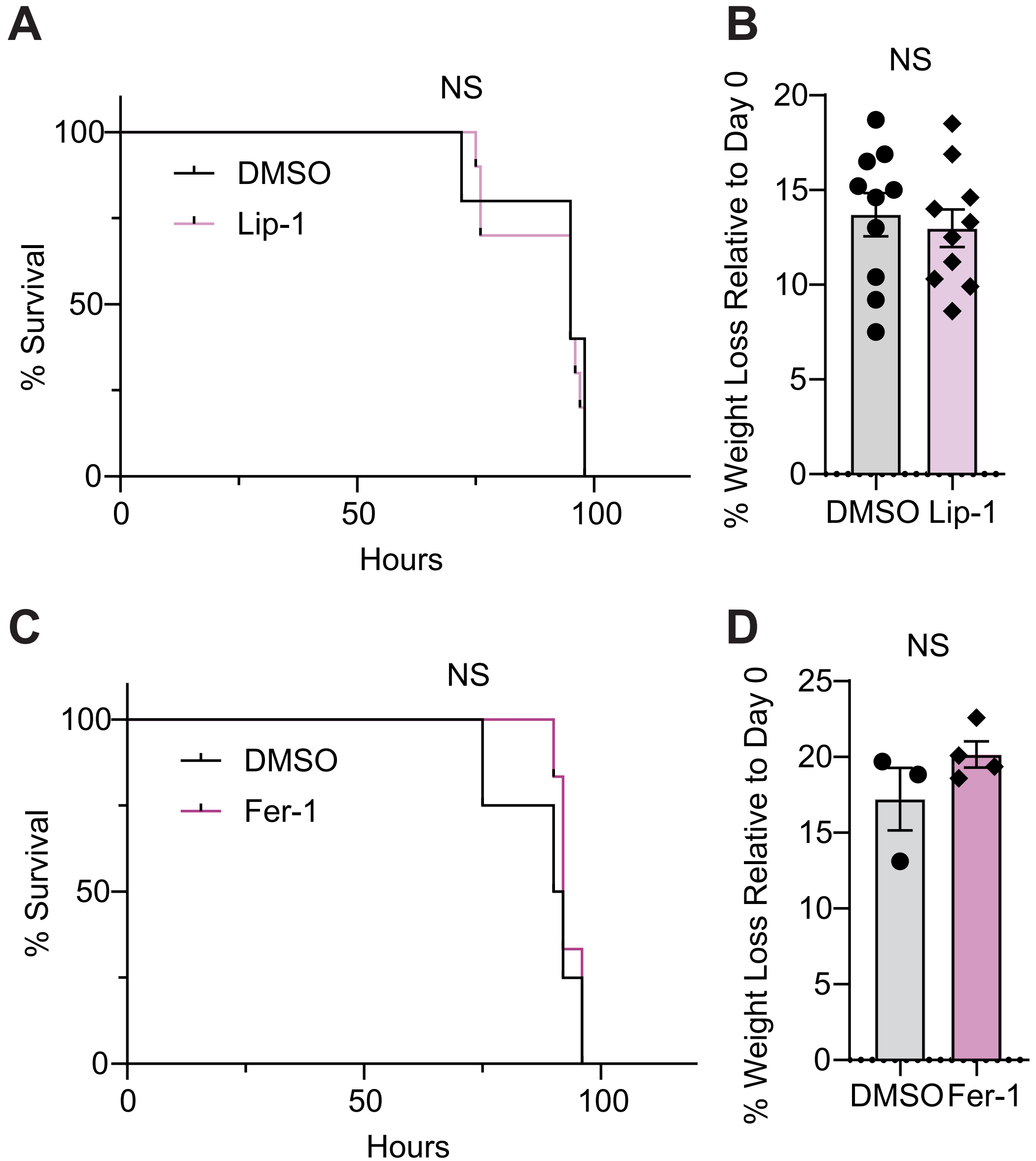

Supplementary Figure 10

Supplementary Figure 10. Liproxstatin-1 and Ferrostatin-1 do not protect against HALI. Percent survival of mice (**A**) and percent weight loss at 96 hours (**B**) in mice treated with intraperitoneal DMSO (n=10) or liproxstatin-1 (Lip-1, n=10) following hyperoxia exposure. Percent survival of mice (**C**) and percent weight loss at 96 hours (**D**) in mice treated with intraperitoneal DMSO (n=3) or ferrostatin-1 (Fer-1, n=4) following hyperoxia exposure. Survival analysis p-values (A, C) by Gehan-Breslow-Wilcoxon test and group comparison p-values (B, D) by unpaired Student's t-test.

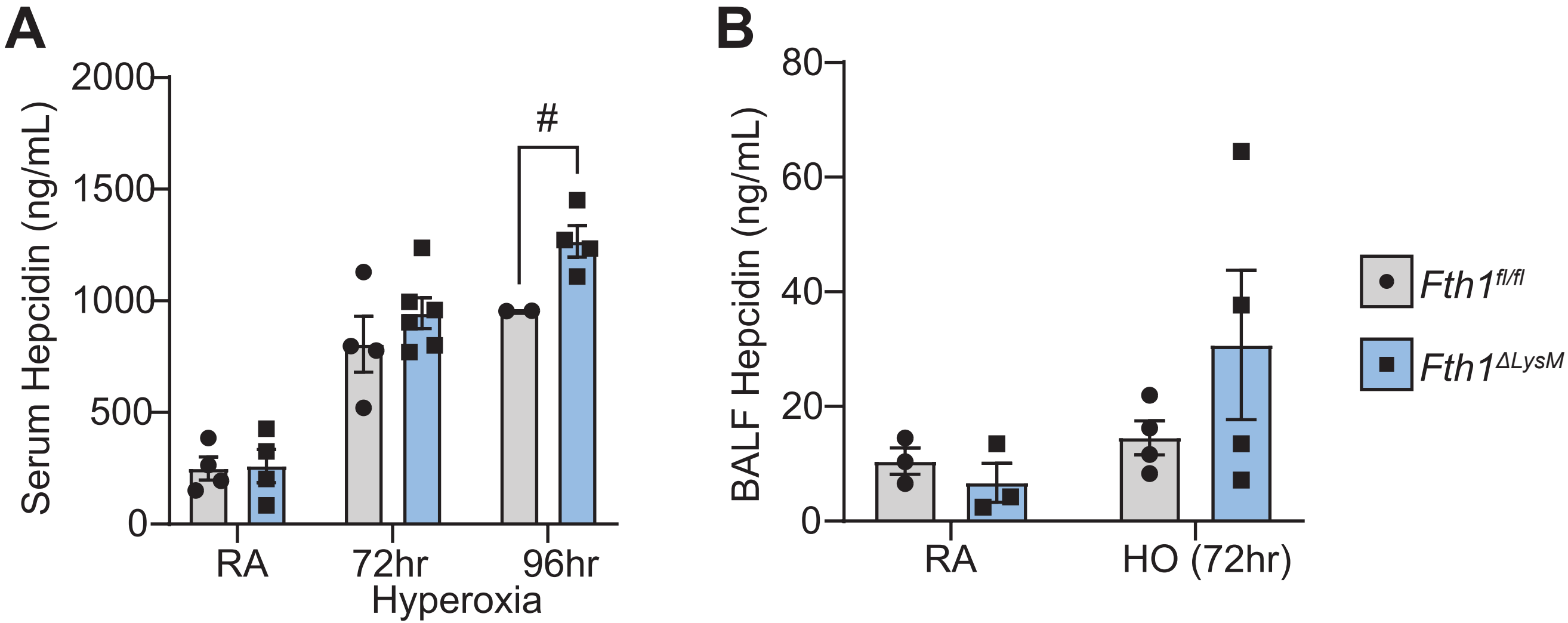

Supplementary Figure 11

Supplementary Figure 11. Serum hepcidin is elevated in hyperoxia. Serum (**A**, n=4/4/2 for *Fth1$^{fl/fl}$* and n=4/6/4 for *Fth1$^{\Delta LysM}$* mice at RA/72hr/96hr hyperoxia conditions) and BALF (**B**, n=3/4 for *Fth1$^{fl/fl}$* and *Fth1$^{\Delta LysM}$* mice in RA/72hr hyperoxia conditions) hepcidin in *Fth1$^{fl/fl}$* and *Fth1$^{\Delta LysM}$* mice in room and following 72 and 96 hours of hyperoxia exposure. Data (A-B) presented mean ± SEM.

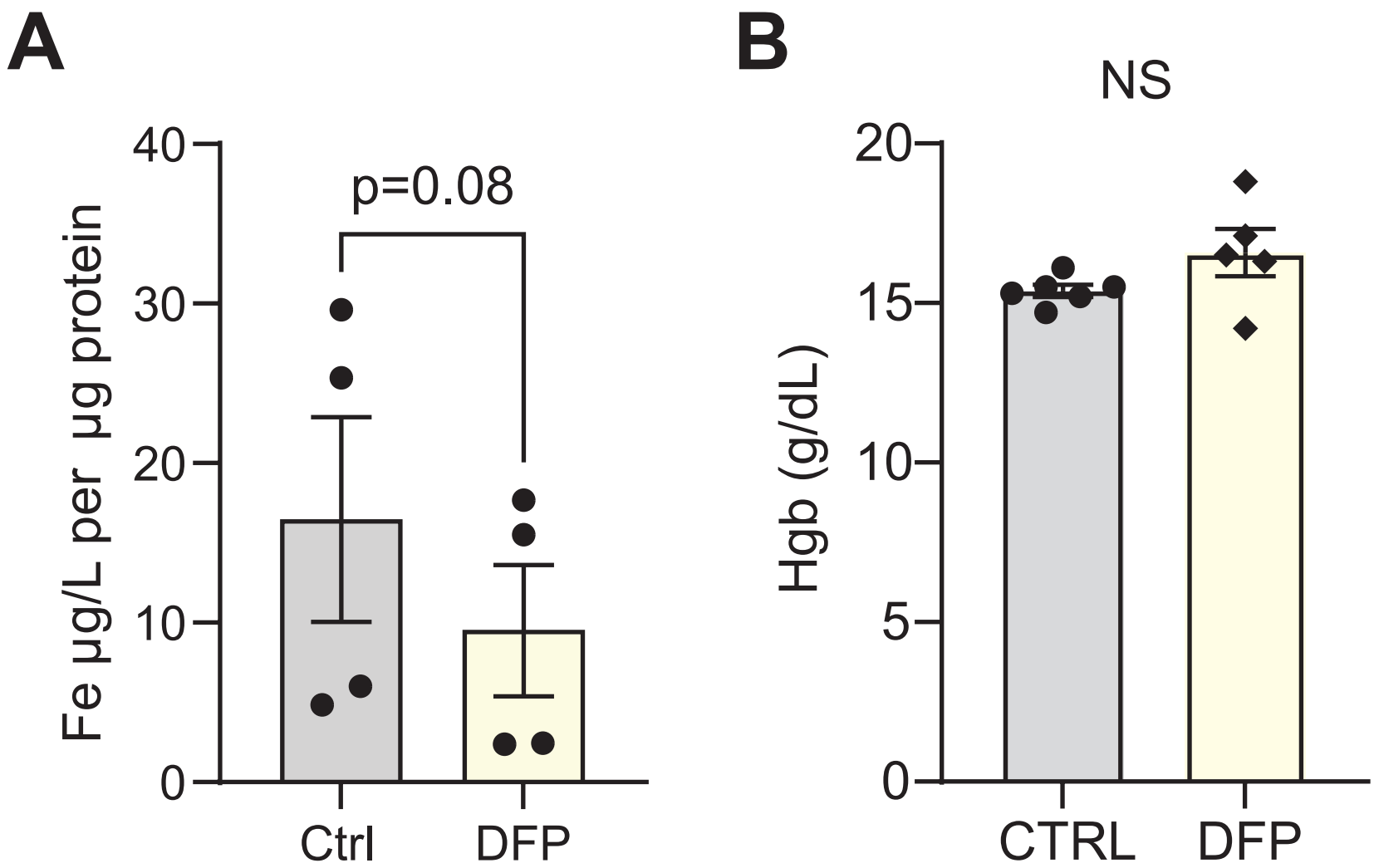

Supplementary Figure 12

Supplementary Figure 12. Deferiprone lowers macrophage total iron levels but does not cause systemic anemia. (**A**) Total iron as measured by graphite furnace atomic absorption spectrometry (GFAAS) in fetal liver-derived macrophages (FLAMs) treated with 24 hours of 100 µM of deferiprone (DFP) relative to control macrophages. (**B**) Plasma hemoglobin levels in mice treated with 7 days of control water or water with 1 mg/mL deferiprone. Data (A-B) presented mean ± SEM with p-value by unpaired Student's t test.

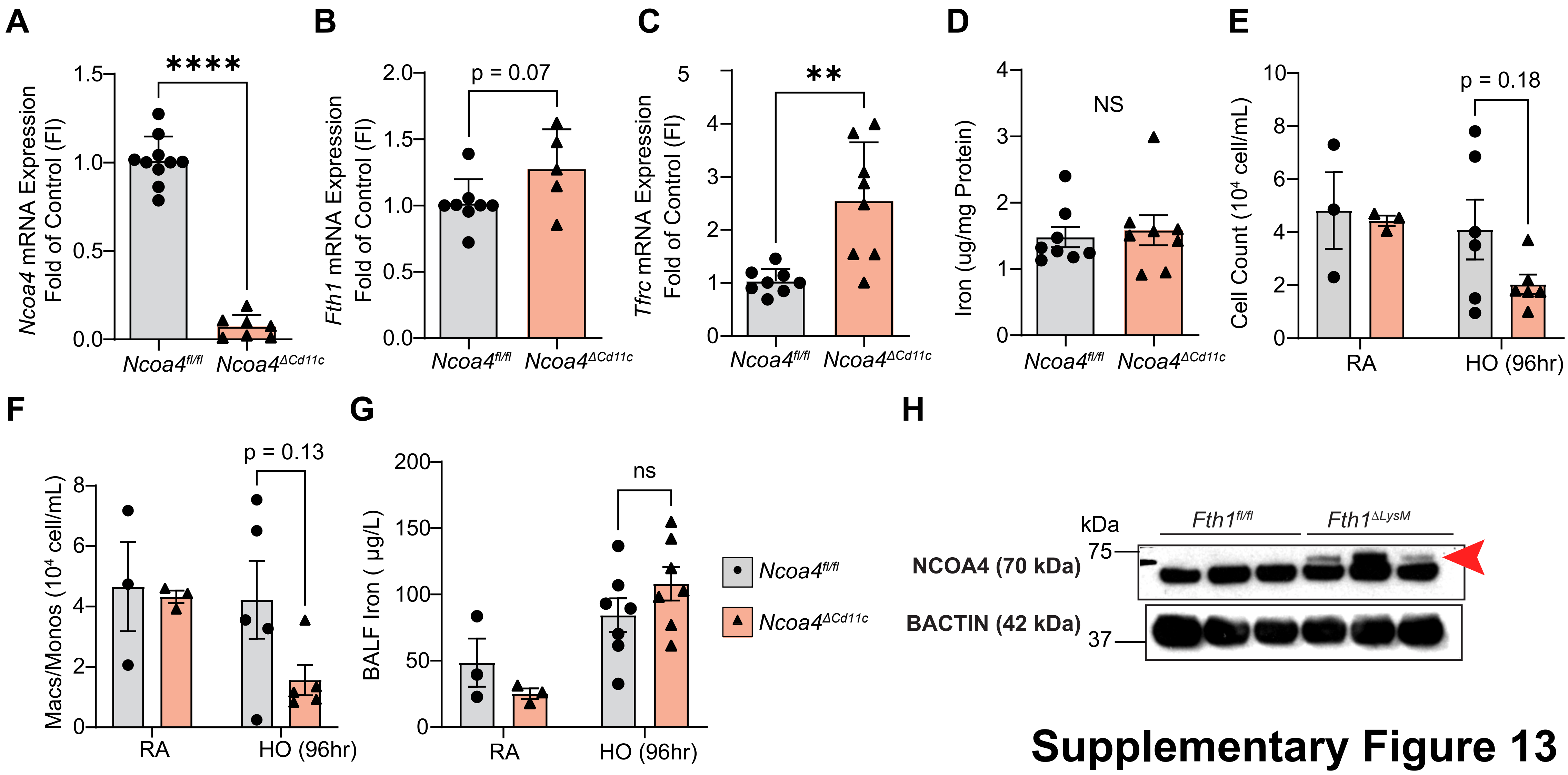

Supplementary Figure 13

Supplementary Figure 13. Alveolar macrophage (AM) NCOA4 depletion alters AM iron metabolism and cellular response to hyperoxia. Real-time polymerase chain reaction (RT-PCR) of AM *Ncoa4* (**A**, n=10 for *Ncoa4*$^{fl/fl}$, n=7 for *Ncoa4*$^{\Delta Cd11c}$), *Fth1* (**B**, n=8 for *Ncoa4*$^{fl/fl}$, n=5 for *Ncoa4*$^{\Delta Cd11c}$) and *Tfrc* (**C**, n=8 for *Ncoa4*$^{fl/fl}$, n=8 for *Ncoa4*$^{\Delta Cd11c}$, p=0.0019) mRNA expression. (**D**) Total iron content in AMs from *Ncoa4*$^{fl/fl}$ and *Ncoa4*$^{\Delta Cd11c}$ mice, normalized to total protein. BALF total cell count (**E**, n=3/6 for both *Ncoa4*$^{fl/fl}$ and *Ncoa4*$^{\Delta Cd11c}$ in RA/hyperoxia conditions), macrophages/monocytes (**F**, n=3/5 for both *Ncoa4*$^{fl/fl}$ and *Ncoa4*$^{\Delta Cd11c}$ in RA/hyperoxia conditions), and total iron by GFAAS in (**G**, n=3/7 for both *Ncoa4*$^{fl/fl}$ and *Ncoa4*$^{\Delta Cd11c}$ in RA/hyperoxia conditions) in RA and hyperoxia-exposed *Ncoa4*$^{fl/fl}$ and *Ncoa4*$^{\Delta Cd11c}$ mice. (**H**) Representative immunoblot of NCOA4 protein expression in bone marrow-derived macrophages (BMDMs) from *Fth1*$^{fl/fl}$ (n=3) and *Fth1*$^{\Delta LysM}$ (n=3) mice; same membrane re-blotted as Figure 3B. Experiment repeated two times with similar results. Data (A-G) presented mean ± SEM with p-values by unpaired Student's t-test (A-D) or 2-way analysis of variance (ANOVA) with Šídák's correction (E-G) for multiple comparisons. **p<0.01, ****P<0.0001.

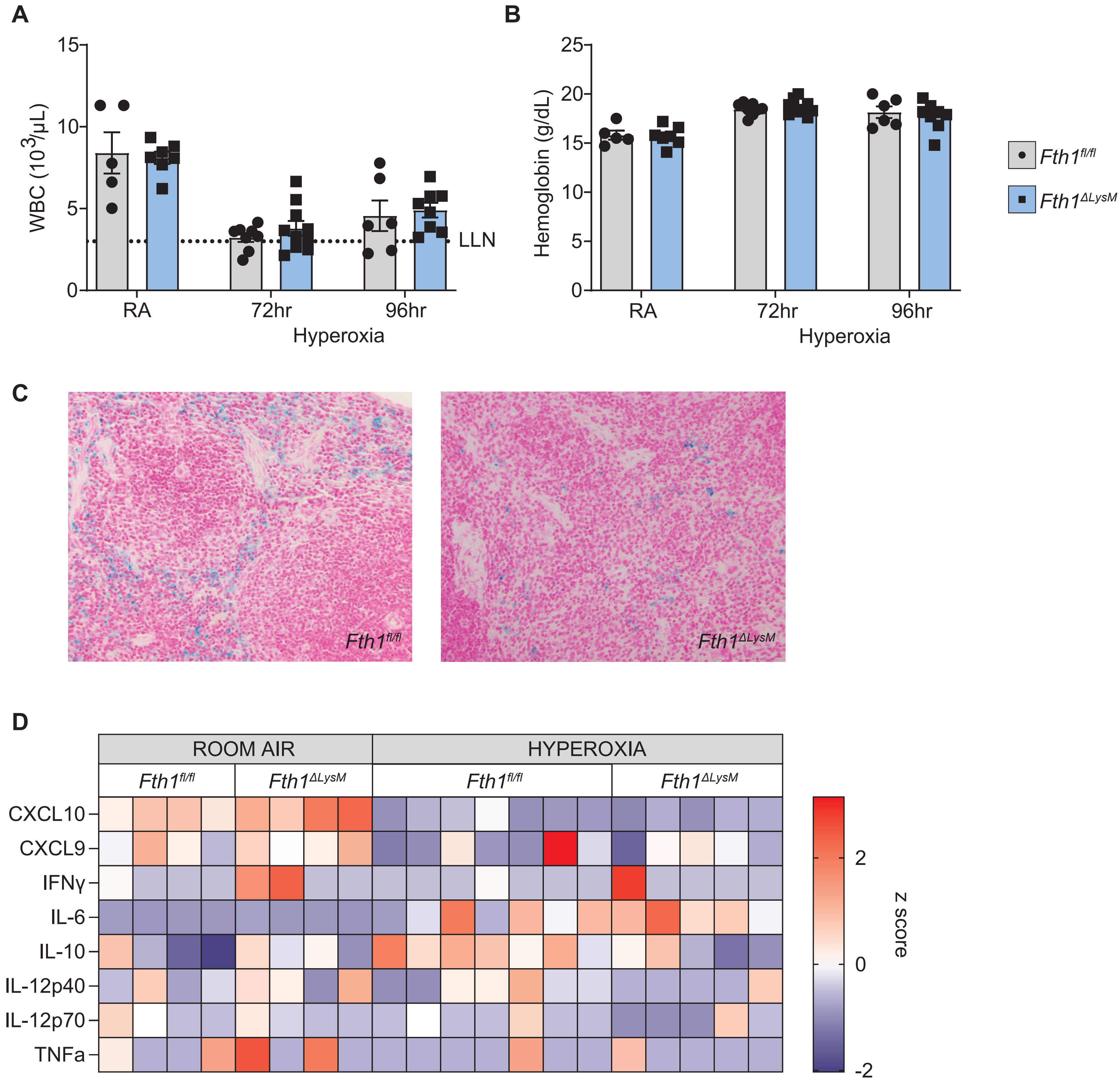

Supplementary Figure 14

Supplementary Figure 14. Hyperferritinemia in *Fth1*$^{\Delta LysM}$ mice and following hyperoxia exposure is not associated with a macrophage activation syndrome. Plasma leukocyte (**A**) and hemoglobin (**B**) in *Fth1*$^{fl/fl}$ and *Fth1*$^{\Delta LysM}$ mice in RA and hyperoxia conditions (n=5/8/6 for *Fth1*$^{fl/fl}$ mice and n=7/10/8 for *Fth1*$^{\Delta LysM}$ mice in RA/72hr/96hr). (**C**) Representative Perls' Prussian blue staining of the splenic sections from *Fth1*$^{fl/fl}$ and *Fth1*$^{\Delta LysM}$ mice following 72 hours of hyperoxia exposure. Iron deposits are observed in the splenic red pulp, indicated by blue staining. (**D**) Heatmap of plasma cytokines from *Fth1*$^{fl/fl}$ and *Fth1*$^{\Delta LysM}$ mice following 72 hours of hyperoxia exposure.

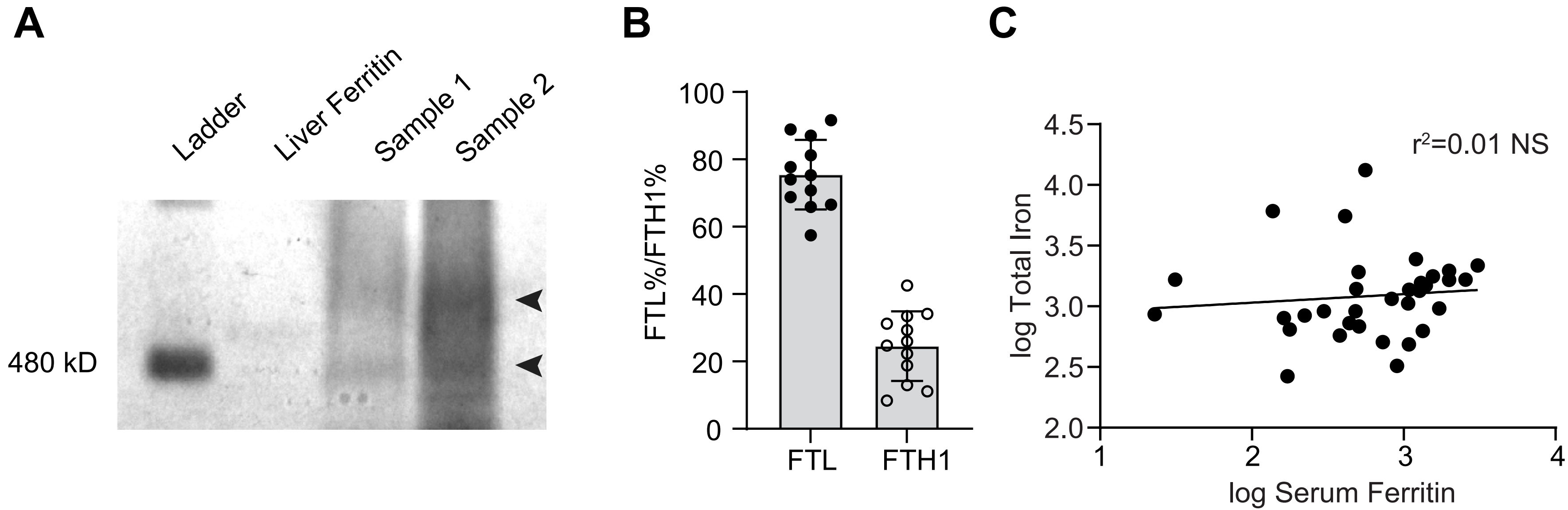

Supplementary Figure 15

Supplementary Figure 15. Serum ferritin in human ARDS is enriched for FTL. (**A**) Representative native electrophoresis gel image from native serum ferritin isolation, with quantification of FTL and FTH1 by mass spectrometry. (**B**) Relative percentage of FTL or FTH1 subunits. (**C**) Log total plasma iron displayed with log serum ferritin in matching subject.

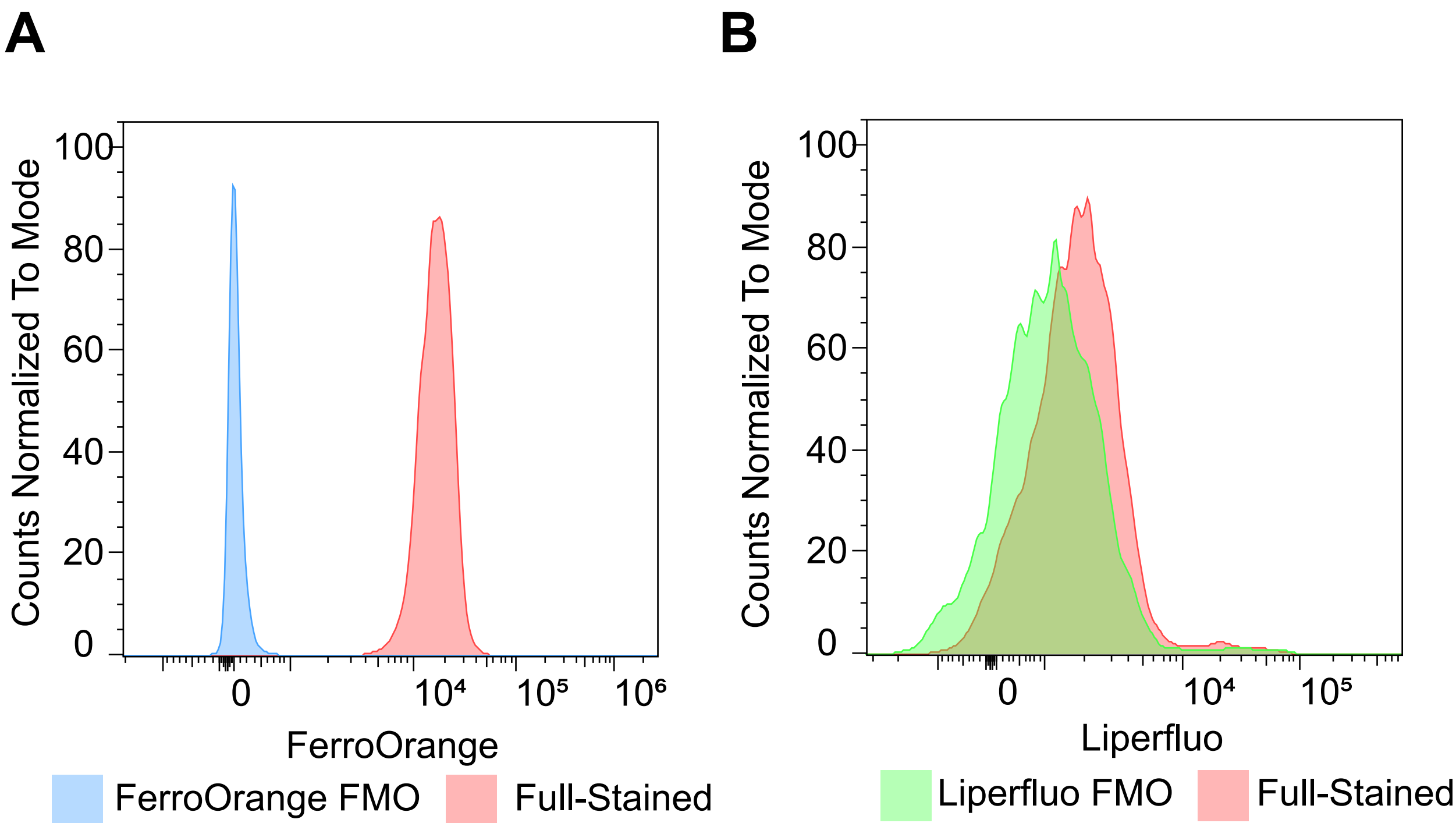

Supplementary Figure 16

Supplementary Figure 16. Fluorescence-minus-one (FMO) control validation of FerroOrange and Liperfluo. FerroOrange (**A**) and Liperfluo (**B**) dye signal in lung $CD11c^{+}$ Siglec-F^{t} AMs, in FMO and full-stained samples.
