# Supplementary material for: Targeting macrophage ferritin heavy chain mitigates ferroptosis and lung injury in experimental acute respiratory distress syndrome
Source: Nat Commun. 2026 Jun 27;17:8057. doi: 10.1038/s41467-026-74828-w (PMC13454589; doi:10.1038/s41467-026-74828-w)
Supplement: Supplementary file 2 [file 41467_2026_74828_MOESM2_ESM.pdf]

nature portfolio

Corresponding author(s): Suzanne Cloonan
William Zhang

Last updated by author(s): May 12, 2026

Reporting Summary

Nature Portfolio wishes to improve the reproducibility of the work that we publish. This form provides structure for consistency and transparency in reporting. For further information on Nature Portfolio policies, see our Editorial Policies and the Editorial Policy Checklist.

Statistics

For all statistical analyses, confirm that the following items are present in the figure legend, table legend, main text, or Methods section.

n/a	Confirmed	
☐	☒	The exact sample size (*n*) for each experimental group/condition, given as a discrete number and unit of measurement
☐	☒	A statement on whether measurements were taken from distinct samples or whether the same sample was measured repeatedly
☐	☒	The statistical test(s) used AND whether they are one- or two-sided<br>*Only common tests should be described solely by name; describe more complex techniques in the Methods section.*
☐	☒	A description of all covariates tested
☐	☒	A description of any assumptions or corrections, such as tests of normality and adjustment for multiple comparisons
☐	☒	A full description of the statistical parameters including central tendency (e.g. means) or other basic estimates (e.g. regression coefficient) AND variation (e.g. standard deviation) or associated estimates of uncertainty (e.g. confidence intervals)
☐	☒	For null hypothesis testing, the test statistic (e.g. *F*, *t*, *r*) with confidence intervals, effect sizes, degrees of freedom and *P* value noted<br>*Give P values as exact values whenever suitable.*
☒	☐	For Bayesian analysis, information on the choice of priors and Markov chain Monte Carlo settings
☒	☐	For hierarchical and complex designs, identification of the appropriate level for tests and full reporting of outcomes
☒	☐	Estimates of effect sizes (e.g. Cohen's *d*, Pearson's *r*), indicating how they were calculated

Our web collection on statistics for biologists contains articles on many of the points above.

Software and code

Policy information about availability of computer code

Data collection	Data collection details was described in the Methods section.
Data analysis	All analyses of clinical data were performed using R version 4.0.2118. Plots were created using the R package ggplot2119, and tables were constructed using the R package gtsummary120. Experimental data were analyzed used GraphPad Prism v10.5.0.

For manuscripts utilizing custom algorithms or software that are central to the research but not yet described in published literature, software must be made available to editors and reviewers. We strongly encourage code deposition in a community repository (e.g. GitHub). See the Nature Portfolio guidelines for submitting code & software for further information.

Data

Policy information about availability of data

All manuscripts must include a data availability statement. This statement should provide the following information, where applicable:

- Accession codes, unique identifiers, or web links for publicly available datasets
- A description of any restrictions on data availability
- For clinical datasets or third party data, please ensure that the statement adheres to our policy

Data availability
Supporting data for all values underlying data are included in the supplementary data summary. Single-nucleus RNA-seq data from lung tissues of 19 COVID-19 decedents and 7 control patients were retrieved from GEO under accession number GSE171524.

April 2023

Research involving human participants, their data, or biological material

Policy information about studies with human participants or human data. See also policy information about sex, gender (identity/presentation), and sexual orientation and race, ethnicity and racism.

Reporting on sex and gender	The demographic information on sex and gender for all human subjects are included in Supplementary Table 1. Gender was not considered in study design.
Reporting on race, ethnicity, or other socially relevant groupings	The demographic information on sex and gender for all human subjects are included in Supplementary Table 1. Race and ethnicity were not considered in study design and did not impact recruitment.
Population characteristics	Other relevant population characteristics are included in Supplementary Table 1.
Recruitment	Subjects were consented prospectively on admission to ICU to participate in the CEDAR registry study.
Ethics oversight	The Institutional Review Board of Weill Cornell Medical College approved this study.

Note that full information on the approval of the study protocol must also be provided in the manuscript.

Field-specific reporting

Please select the one below that is the best fit for your research. If you are not sure, read the appropriate sections before making your selection.

☒ Life sciences ☐ Behavioural & social sciences ☐ Ecological, evolutionary & environmental sciences

For a reference copy of the document with all sections, see nature.com/documents/nr-reporting-summary-flat.pdf

Life sciences study design

All studies must disclose on these points even when the disclosure is negative.

Sample size	Sample size was determined by our prior experience working with the hyperoxia model, with power calculations made using GPower 3.1.
Data exclusions	There was no specific exclusion criteria in this study.
Replication	At least 3 biological replicates were attempted for all experiments, with all experiments attempted at least 2 times; all data were included unless there was a technical reason to exclude an experiment, regardless of the outcome.
Randomization	Animals were randomly allocated, matched by age and sex and ensuring relatively equal numbers of genetically modified and control animals.
Blinding	Readers were blinded for all studies involving subjective interpretation of data (i.e. histology and other microscopy studies).

Reporting for specific materials, systems and methods

We require information from authors about some types of materials, experimental systems and methods used in many studies. Here, indicate whether each material, system or method listed is relevant to your study. If you are not sure if a list item applies to your research, read the appropriate section before selecting a response.

Materials & experimental systems

n/a	Involved in the study	
☐	☒	Antibodies
☒	☐	Eukaryotic cell lines
☒	☐	Palaeontology and archaeology
☐	☒	Animals and other organisms
☐	☒	Clinical data
☒	☐	Dual use research of concern
☒	☐	Plants

Methods

n/a	Involved in the study	
☒	☐	ChIP-seq
☐	☒	Flow cytometry
☒	☐	MRI-based neuroimaging

Antibodies

Antibodies used	Flow Cytometry:<br>Anti-CD16 and anti-CD32 antibodies (clone 2.4G2; BD Biosciences lot#9248075, 1:1000); anti-CD45 (clone 30-F11; BD Biosciences lot#4276899, 1:400), anti-Siglec-F (clone 1RNM44N, eBioscience lot#2883796, 1:200 or clone E50-2440 BD Biosciences), anti-CD11c

	(clone HL3; BD Biosciences lot#9345738, 1:400), anti-CD11b (clone M1/70; BioLegend lot#B293045, 1:400), anti-Ly6C (clone HK1.4; BioLegend lot#5059620, 1:200), and anti-Ly6G (clone 1A8; BioLegend lot#B353758, 1:200).<br><br>Western Blot:<br>FTH1 (CST D1D4 Lot#4, 1:1000); xCT (Abcam ab175186 lot#GR3337924-1, 1:1000); GPX4 (Abcam ab125066 lot#GR3369574-5, 1:1000); xCT (Abcam ab307601, 1:1000), FTL (Abcam ab109373 lot#1001599-14, 1:1000); NCOA4 (Bethyl A302-272A, 1:1000); TFRC (Abcam ab214039 lot#1015342-43, 1:1000); BACTIN (Sigma A2228 lot#000130966, 1:10,000)
Validation	FTH1 monoclonal antibody is produced by immunizing animals with a synthetic peptide corresponding to residues near the carboxy terminus of human FTH1 protein and is validated in our KO animals; https://www.cellsignal.com/products/primary-antibodies/fth1-d1d4-rabbit-monoclonal-antibody/4393.<br><br>NCOA4 polyclonal antibody was affinity purified using an epitope specific to ARA70 immobilized on solid support and was validated in our KO animals; https://www.fortislife.com/products/primary-antibodies/rabbit-anti-ara70-antibody/BETHYL-A302-271.<br><br>GPX4: https://www.abcam.com/en-us/products/primary-antibodies/glutathione-peroxidase-4-antibody-epncir144-ab125066<br>xCT: https://www.abcam.com/en-us/products/primary-antibodies/xct-antibody-epr27115-64-ab307601<br>FTL: https://www.abcam.com/en-us/products/primary-antibodies/ferritin-light-chain-antibody-epr5260-ab109373<br>TFRC: https://www.abcam.com/en-us/products/primary-antibodies/transferrin-receptor-antibody-epr20584-ab214039<br>BACTIN: https://www.sigmaaldrich.com/US/en/product/sigma/a2228?srsltid=AfmBOopn5sgrgFHcDYKhxy-zKczEzTL9iQykH4olQ93F3YYG-nxV7ZRc

Animals and other research organisms

Policy information about studies involving animals; ARRIVE guidelines recommended for reporting animal research, and Sex and Gender in Research

Laboratory animals	Laboratory animals are described in the Methods section. Fth1 floxed (Fth1fl/fll) mice on a C57BL/6J background were kindly provided by the laboratory of Dr. Anupam Agarwal at the University of Alabama at Birmingham (originally generated by Dr. Lukas C. Kuhn, EPFL, Switzerland). They were crossed with Lysz2Cre mice (also known as LysM-Cre, The Jackson Laboratory, Stock No. 004781) and ItgaxCre (also known as Cd11cCre, The Jackson Laboratory, Stock No. 008068) to generate constitutive Fth1ΔLysM and Fth1ΔCd11c mice. Ncoa4 floxed mice (created and shared by Dr. Joseph Mancias, now deposited in the Jackson Laboratory Stock No. 033295) were crossed with Cd11cCre mice to generate constitutive Ncoa4ΔCd11c mice.
Wild animals	The study did not involve wild animals.
Reporting on sex	Animals of both sex were used in experimental models.
Field-collected samples	The study did not involve field-collected samples.
Ethics oversight	All murine experiments were approved by the Weill Cornell Medicine Institutional Animal Care and Use Committee.

Note that full information on the approval of the study protocol must also be provided in the manuscript.

Clinical data

Policy information about clinical studies

All manuscripts should comply with the ICMJE guidelines for publication of clinical research and a completed CONSORT checklist must be included with all submissions.

Clinical trial registration	*Provide the trial registration number from ClinicalTrials.gov or an equivalent agency.*
Study protocol	*Note where the full trial protocol can be accessed OR if not available, explain why.*
Data collection	*Describe the settings and locales of data collection, noting the time periods of recruitment and data collection.*
Outcomes	*Describe how you pre-defined primary and secondary outcome measures and how you assessed these measures.*

Plants

Seed stocks	*Report on the source of all seed stocks or other plant material used. If applicable, state the seed stock centre and catalogue number. If plant specimens were collected from the field, describe the collection location, date and sampling procedures.*
Novel plant genotypes	*Describe the methods by which all novel plant genotypes were produced. This includes those generated by transgenic approaches, gene editing, chemical/radiation-based mutagenesis and hybridization. For transgenic lines, describe the transformation method, the number of independent lines analyzed and the generation upon which experiments were performed. For gene-edited lines, describe the editor used, the endogenous sequence targeted for editing, the targeting guide RNA sequence (if applicable) and how the editor was applied.*
Authentication	*Describe any authentication procedures for each seed stock used or novel genotype generated. Describe any experiments used to assess the effect of a mutation and, where applicable, how potential secondary effects (e.g. second site T-DNA insertions, mosiacism, off-target gene editing) were examined.*

Flow Cytometry

Plots

Confirm that:

☒ The axis labels state the marker and fluorochrome used (e.g. CD4-FITC).

☒ The axis scales are clearly visible. Include numbers along axes only for bottom left plot of group (a 'group' is an analysis of identical markers).

☒ All plots are contour plots with outliers or pseudocolor plots.

☒ A numerical value for number of cells or percentage (with statistics) is provided.

Methodology

Sample preparation	Detailed methods are described in the Method section.
Instrument	BD Symphony A5 flow cytometer (BD Biosciences)
Software	FlowJo v10.8 software (BD Biosciences)
Cell population abundance	At least 10000 events were acquired for each sample.
Gating strategy	Gating strategy is shown in Figure 3M/Supplementary Figure 9A. Positive and negative populations were determined by comparing single color-stained cells with unstained cells and/or beads.

☒ Tick this box to confirm that a figure exemplifying the gating strategy is provided in the Supplementary Information.
