# Supplementary material for: Targeting macrophage ferritin heavy chain mitigates ferroptosis and lung injury in experimental acute respiratory distress syndrome
Source: Nat Commun. 2026 Jun 27;17:8057. doi: 10.1038/s41467-026-74828-w (PMC13454589; doi:10.1038/s41467-026-74828-w)
Supplement: Supplementary file 3 [file 41467_2026_74828_MOESM3_ESM.pdf]

nature portfolio

Peer Review File

Targeting macrophage ferritin heavy chain mitigates ferroptosis and lung injury in experimental acute respiratory distress syndrome

Corresponding Author: Professor Suzanne Cloonan

This file contains all reviewer reports in order by version, followed by all author rebuttals in order by version.

Version 0:

Reviewer comments:

Reviewer #1

(Remarks to the Author)
Acute respiratory distress syndrome (ARDS) is a type of respiratory failure, which could be induced by many direct or indirect causes. Previous studies have found some relations between ARDS and iron status or ferritin level. However, the role of extracellular ferritin is remains unclear. In this study, the authors demonstrated a correlation between the changes of ex-ferritin and the severity of ARDS. Furthermore, using Fth1-LysM and Fth1-Cd11c conditional knockout mice, the authors found Fth1 rich ex-ferritin promoted hyperoxia induced ARDS through neutrophil activation. This study has interesting. Some key issues that need to be addressed.

1. The authors have observed that macrophage Fth1 deficiency has protective effect on hyperoxia-induced lung injury in mice and supposed that knockout Fth1 in macrophage reduced the Fth1 rich ex-ferritin level, and then inhibited neutrophil activation. Although this hypothesis is reasonable, how to exclude the direct effect of Fth1 knockout on macrophage function and viability.

2. Why only study macrophage Fth1? How the authors exclude other cell type ferritin function in models?

3. For Figure 4D, if the Fth1 is deficient, the intracellular labile iron level will be elevated. Actually, the elevated labile iron will promote ferroptosis. And the elevated Gpx4 and Slc7a11, and reduced Tfrc could be explained by compensate protective effect. But overall, the ferroptosis should be activated. How could it happen, as shown in Figure 4F, Fth1 deficiency inhibited RSL3 induced ferroptosis in mice BMDM?

4. Figure 5J, Ncoa4 deficiency inhibited ferritinophagy, thus elevated ferritin level, and then protected cell viability against RSL3 induced ferroptosis, which is consistent with previous studies, but contrary to the hypothesis in this study?

5. Figure 6N, the injury effect was mainly mediated by neutrophil activation. Actually, neutrophilic infiltration has multiple effects on inflammation and injury, including NETosis. How could we say ferroptosis is most important in neutrophil mediated lung injury?

6. All of the mice studies suggested higher Ftl1/Fth1 ratio have protective effect, but why the died individual in the COVID-19 study have higher FTL/FTH1 ratio compared with the survivers as shown in Figure 6M?

7. The authors have found alveolar injury and alveolar epithelial cell death as shown in Figure 2A and Figure 3G. But only macrophage was studied in vitro, whereas no epithelial cell was studied. Similarly, no epithelial cell information is showed in the overview schematic of the study in Figure 6N. Actually, alveolar epithelial cell injury is more important than macrophage injury in ARDS. Macrophage could be a causer or mediator, but not the target cell.

8. Specific ferroptosis inhibitors in vivo will help support the conclusion.

9. Lack of genetic models to support ferroptosis working model

Reviewer #2

(Remarks to the Author)
Summary: This study by Zhang et al investigating the role of ferritin in macrophages and monocytes and induction of ARDS in mice. Authors start the paper with data from COVID-19 patients and then move to the HALI model of ARDS. This Reviewer questions the HALI model of ARDS and relevance to COVID-19 patients (see below). The manuscript reads well. Authors developed cutting edge mouse models including multiple conditional deletions in myeloid cells and macrophages, which is relevant to the study. This Reviewer however questions the lack of cell sorting in some experiments and in general the lack of Immunology readouts such as flow cytometry, for instance for all experiments investigating cell numbers. Some of the RNAseq pathway analysis is weak, with only one gene in some highlighted pathways. Please see below for more detailed comments.

Major comments.

1) Not all COVID-19 patients develop ARDS. Therefore, what are the markers used by the authors, if any, to conclude that the data set is only focusing on COVID-19 ARDS? If no data is available, the text should read that the data set is on COVID-19 patients, not COVID-19 ARDS. Lines 97, 116, 120 etc are examples. This issue is throughout the manuscript.

2) Related to these comments: Line 165: is COVID-19 and “human ARDS” interchangeable?

3) Figure 1C. This shows baseline Ferritin, but are these really baseline? The levels of “baseline” ferritin are not available (pre-infection by COVID-19) and since inflammation affects ferritin and iron metabolism in general (inflammation-anemia), these values are most probably not baseline. They reflect ferritin upon entry in ICU, which is probably very variable between patients. The authors should make this clear to the readers.

4) Figure D. Not clear why only macrophages and monocytes are labeled. All cells should be labelled.

5) Figure 1J. Could this downregulation be seen in the lungs? (ie panel E)? Since panel J is blood, it is hard to compare with lung monocytes. What are the dynamics of FTH1 expression in macrophages with disease severity?

6) What is the rationale for choosing HALI over other models. Since authors emphasize the relationship between COVID-19 and ARDS. There are other models that replicate better viral-induced ARDS than HALI.

7) Line 168. Please show BAL cellularity by flow cytometry. It is true that macrophages are present in excess, but monocytes and neutrophils should also be well represented, as per the author’s definition of clinical readouts of ARDS. This is a requirement to interpret Figure 2E-G as it is hard to believe these BALs are only macrophages.

8) Related to the previous point. Authors should sort macrophages from the BAL prior qPCR. Since different results were seen using in LPS model of ARDS.

9) Figure 2L. Please explain why “response to bacterium” is upregulated in HALI, which does not include bacterial infection. What genes does this pathway take into account? Also, the size of the “ No of genes” legend does not match the actual plots. Overall, there seems to be a very limited number of genes in these pathways, especially panel M. Are these enough to claim they are enriched?

10) Line 197. Related to point above, as long as there is no proof that the BAL cells are ALL AM, it is not possible to conclude that macrophages upregulated ferritin at the transcriptomic level.

11) Figure 3B. How do the authors explain the increased protein concentration in the cre group at 72h, and a decrease 24h after? Is this physiological?

12) Figure 3I-K. It would be relevant to include flow cytometry analysis of these myeloid cells. Flow cytometry would also allow to quantify the cellular iron in each population.

13) About BMDMs in Figure 1 and Figure 3. Authors derive BMDM from the bone marrow of mice that went through induction of ALI. How physiologically relevant are these BMDMs compared to macrophages that are actually present at the site of inflammation in the lungs? It would be more relevant to perform RNAseq on AMs isolated from the lungs of mice with ARDS…

14) Figure 4B. How many genes are in the “Response to L-ascorbic acid” pathway. Is this enough to claim that the pathway is “enriched”? Same question for the ferroptosis pathway in Figure 4C. Based on the legend it corresponds to one gene – not enough to claim the pathway is enriched. Most of the pathways in Figure 4C are representing 1-3 genes in each pathway, this is a very poor analysis.

Minor comments.

Line 203. “robustly” may be an overstatement.

Lines 414-415. The word “that” is repeated

Reviewer #3

(Remarks to the Author)
Zhang et al. investigated the role of macrophage ferritin (particularly FTH1) as a potential factor modulating immune response and disease severity in different models of acute respiratory distress syndrome (ARDS). The study offers intriguing insights into how iron metabolism interacts with the immune system. It supports previous suggestions in the literature about the potential immunomodulatory role of ferritin, a topic that has not been thoroughly explored until now.
To study their hypothesis, the Authors performed a series of extensive and well-designed experiments, starting from cohorts of COVID-19 patients and subsequently moving toward hyperoxia-induced acute lung injury (HALI) mouse models.
The study is valuable and characterized by experiments carried out with validated and reproducible techniques.
Nevertheless, this complex paper includes some inconsistencies and contradictions (also regarding certain methodological choices) that require clarification and more robust arguments before being considered for publication.
Below, we provide a list of general and study-specific comments to improve the paper and its readability.

General Comments
1. Text needs English editing, as there are repetitions, typos, etc.
e.g. Line 82-83: “in the setting of ARDS” and “in individuals with ARDS” appear a repetition in the same sentence
e.g. Line 387: “in” is repeated
e.g. Line 446: “that” should be deleted
2. The manuscript should be ameliorated in terms of readability and clarity. There are some redundant parts and some inconsistencies throughout the text.
e.g. please uniform PBMC/PBMCs; BAL/BALF; BMDM/BMDMs; HALI/hyperoxia-induced ALI across the whole manuscript
3. Results section: this section should be amended, as it is full of “discussion-like comments.” The results section should contain just the description of the results without hypothesis on possible explanatory mechanisms or speculation on causes/effects.
4. The conclusion section should be renamed as “discussion”.
5. Some of the speculations/hypotheses made by the authors are too assertive and should be mitigated.
e.g. Line 87-88: the sentence “we hypothesized that the main source for ex-ferritin in critical illness is the macrophage” refers to a piece of data that is at least partially already demonstrated, so the authors could reformulate it accordingly.

6.The paper focuses on the role of the ferritin H-subunit. Did the authors assess the levels of ferritin-bound iron? This data could provide valuable insights into whether the observed effects were due to ferritin itself or are mediated by the iron it carries.

7. A key player in iron metabolism, that is, hepcidin, has not been considered in the experimental design. Hepcidin is strongly regulated by pro-inflammatory cytokines and plays a pivotal role in orchestrating iron distribution between intracellular and extracellular (plasma) iron levels during acute inflammation. Beyond the systemic effects of liver-derived hepcidin, this hormone is also produced locally by a variety of cells, including alveolar macrophages. Why did the authors not measure, for example, hepcidin levels in the bronchoalveolar lavage fluid (BALF) and in the serum of both patients and mouse models?
This limitation should be acknowledged and discussed.

Study Specific Comments
Results:

Paragraph “1” Serum ferritin trajectory associates with increased mortality in COVID-19 ARDS.
Line 101: the authors talk about 383 subjects, while in Lines 108 and 110: the IOT/deceased patients are 119, the IOT/survivors are 213 (total 332); in Suppl Tab 1: the IOT/deceased patients are 157, the IOT/survivors 249 (total 406). Could the authors clarify these discrepancies?
Suppl Tab 1: there are three columns; could the authors clarify what the p refers to? only to the comparison between the first two columns or between all of them?
Lines 116-117: the Authors state that “ex-ferritin levels rise with increasing ARDS disease severity in COVID-ARDS and associate with worse clinical outcomes”; however, data in Suppl Tab 1 seems to highlight that IOT/deceased patients were older and with a higher prevalence of some comorbidities; could the authors comment on this aspect? Additionally, was ferritin delta an independent predictor of mortality after adjusting for age and comorbidities?

Paragraph “2” FTH1 and FTL expression are enriched in monocytes and lung macrophages in individuals with ARDS

Lines 146-147: the authors affirm that “ferritin transcripts are highly enriched in myeloid cells within the lungs and peripheral blood of critically ill COVID-19 patients”. However, this seems to contradict the observation that the expression of FTH1 and FTL in monocytes of subjects with severe/lethal COVID19 is reduced. Could the authors please clarify this point? Please consider modifying the title of the paragraph accordingly.

Paragraph “3” Hyperoxia-induced ARDS modulates macrophage ferritin expression and extracellular ferritin
Lines 171-173: In these lines, the authors mention a different mouse model with acute lung injury, which is not mentioned again in the text. I wonder if the authors intended to emphasize a difference in ferritin expression, specifically in an inflammatory lung injury model rather than in a hyperoxia model. However, this intention is not clear in the current text. I suggest the authors provide justification or a commentary on this or consider omitting this data.
Lines 196-197: the authors state that “These findings indicate that hyperoxia significantly elevates FTH1 expression and to a lesser extent FTL, in AMs (alveolar macrophages) and in monocyte-derived macrophages”. However, in the text, it appears that FTL expression is increased only in BALF macrophages, while it remains unchanged in bone marrow-derived cells. Can the authors clarify this point? When the authors mention monocyte-derived macrophages, are they referring to BMDMs?

Paragraph “5” Macrophage FTH1 depletion alters the cellular inflammatory response to hyperoxia
Lines 239-241: the authors state that “This early onset of neutrophil infiltration in the Fth1ΔLysM mice at 72 hours mirrored higher BALF protein (p=0.04) and a trend for higher BALF IgM at 72 hours compared to Fth1fl/fl mice”. This sentence refers to observations made at 72 hours and seems to contradict the information presented in the previous paragraph (Lines 214-216), where proteins and IgM concentrations in the BALF were measured at 96 hours. The authors should strive for consistency in reporting time points, as discrepancies between 72 and 96 hours may confuse the readers. In this case, omitting the data collected at 72 hours may be advisable.
Lines 252-254: the authors state that “the lower airway macrophage and monocyte numbers in control Fth1fl/fl relative to that of Fth1ΔLysM mice may not be due to the absence of chemotactic signals”. Could the authors clarify why they also mention a smaller number of monocytes?

Paragraph “6” FTH1 depletion protects macrophages from ferroptosis.
Lines 267-275: It is unclear whether the hyperoxia exposure lasted for 72 or 96 hours and what the difference is between mice evaluated at baseline versus those in room air. Could the authors clarify these aspects in more detail?
Line 279: Ferritin expression should decrease when cellular iron content declines. Could the authors comment on the increase of Ftl expression in FTH1-depleted (iron deficient) BMDMs?

Paragraph “7”, The intracellular iron chelator deferiprone exacerbates hyperoxia-induced lung injury
Lines 322-324: these lines seem to report the same data already mentioned in lines 279-280 about the decrease in Tfrc and the increase in ferroportin expression
Lines 324-327: the authors state that “This hypothesis is further supported by our observation that Fth1ΔLysM BMDMs are sensitive to exogenous iron as iron added in the form of ferric ammonium citrate (FAC) rapidly decreases their viability relative to Fth1fl/fl cells over 24 hours”. This observation seems to contradict the fact that Fth1ΔLysMBMDMs cells acquire less iron and release more iron than the wild-type model. Could the author explain why Fth1ΔLysMBMDMs, despite acquiring less iron and releasing more, experience a greater viability reduction after FAC exposure than the normal Fth1fl/fl model?
Lines 339-337: To effectively test the study hypothesis, the authors should have established selective iron depletion in macrophages. The use of systemic deferiprone (DFP) leads to a state of global iron deficiency, impacting other organs and tissues as well. Previous studies have indicated that iron deficiency and iron deficiency anemia can exacerbate certain lung diseases. Did the authors assess levels of hemoglobin (Hb) and ferritin in the murine model treated with DFP?

Paragraph “8” Impaired macrophage ferritin recycling exacerbates hyperoxia-induced ALI
Lines 364-366: I have some concerns regarding the authors' statement and the mouse model used to test their hypothesis. The authors claim that FTH1-depleted mice are protected from hyperoxia damage (paragraph 1) and that FTH1 depletion protects macrophages from ferroptosis (paragraph 2). However, they also report an increase in NCOA4 levels, while the literature suggests that the loss of NCOA4 is protective against ferroptosis. Did the authors consider whether the observed protective effect against ferroptosis in the FTH1-depleted mouse model be attributed not to the loss of NCOA4, but to a lack of proper interaction between NCOA4 and FTH1?

Paragraph “9” Loss of macrophage FTH1 results in lower extracellular iron and increased FTL ex-ferritin which is protective against hyperoxia
General: The ferritin levels observed by the authors are extremely high and approach those typically seen in certain macrophage activation syndromes. Did the authors also investigate what happens to other indicators of macrophage activation at a systemic level (cytopaenias, transaminase elevation, coagulopathy, increased soluble IL-2 receptor (CD25), soluble CD163, IL-18, etc.)? This would be essential to improve the quality of the manuscript. As mentioned earlier, the increased expression of Ftl in the bone marrow-derived macrophages (BMDMs) of FTH1-depleted mice appears to be controversial, considering these macrophages are iron-depleted. Does the increased expression of Ftl sufficiently account for the significant rise in ferritin levels? Was hemophagocytosis present? Is there evidence of liver cytolysis or necrosis?
Line 378: data concerning serum and BALF iron are reported at different time points (72 and 96 hours, respectively); could the author why they used different time points?
Lines 397-398: the authors affirm that “The above data suggests that upon loss of FTH1 in macrophages, FTL is increased and secreted into the extracellular space”. However, while the authors hypothesize that it is ferritin L, they have not quantified the presence of ferritin H or L in the extracellular space. I suggest that the authors revise the statement accordingly.

Lines 400-403: Authors hypothesize that in the context of elevated serum ex-ferritin in human ARDS, ex-ferritin, specifically FTL, not only represents a marker of cellular damage but a way to curb extracellular iron levels to protect from further ferroptosis. Unfortunately, this hypothesis appears to contradict existing literature, which has indicated that higher ferritin levels are an independent predictor of adverse outcomes in some ARDS models, including COVID-related ARDS? Could the authors comment on this aspect?

Paragraph "10" Serum ex-ferritin consists predominantly of FTL in human ARDS.
General: The observation that patients with deceased ARDS had higher L-ferritin levels than survivors contradicts the prior paragraph's hypothesis and requires the authors' clarification. Could the authors please clarify at what time point the dosage of the FTL and FTH1 was performed?

Version 1:

Reviewer comments:

Reviewer #2

(Remarks to the Author)
The authors conducted numerous additional experiments and adequately addressed all the criticisms.

Reviewer #4

(Remarks to the Author)
Zhang et al. explored the protective role of macrophage derived ferritin in ARDS. They start with presenting the enrichment of ferritin in individuals with ARDS and HALI mice model, compared to controls. This finding aligns with the strong association between ferritin level and severity of covid-19 induced ARDS reported by others. Using several genetic modified mice models, they demonstrated that FTH in macrophages is detrimental while secreted FTL is protective in HALI. The study brings new insights into the iron immunity field. However, some parts of their work seemed contradictory to current understanding of iron induced ferroptosis, and conclusions are not firmly supported by their data. I will focus on the concerns previously raised by the reviewer 3.
General comments:
The authors have properly addressed C1-C5, C7; although they didn't provide the data of ferritin bound iron for C6, the current version is sufficient to support that ferritin itself is more relevant in their context.
Study Specific Comments
The authors have properly addressed C8-C12, C14-C28.
For C13, the authors provided data from BMDM to further support that hyperoxia induces FTH and FTL in macrophages. However, the blot in Figure 2L requires quantification due to uneven sample loading.
One extra comment:
The methodology for assessing ferroptosis in FTH KO BMDM and FTH LysMKO mice is deemed too simplistic. If FTHKO macrophages are more sensitive to ferroptosis, the protective phenotype they observed in HO FTHKO mice could be attributed to a loss of pathogenic macrophages. A more integrative experimental design could help clarify these inconsistencies and improve the power of their findings. Therefore, it is strongly recommended that:
1. Flox and FTHKO BMDM can be exposed to FAC, RSL-3 in HO/RA conditions for 4, 12 and 24hours. Cell death should be determined using ANNEXIN V/Sytox staining in a flow cytometer;
2. Lipid peroxidation in BMDM treated as above could be determined using C11-BODIPY staining, and determined in a flow cytometer;
3. The labile iron pool in the macrophages from FTH LysMKO mice and control mice exposed to HO/RA should be illuminated using ferroorange staining in BALF as well as single-cell suspension from digested lung, and then determined in a flow cytometer;
4. Lipid peroxidation in the macrophages from FTH LysMKO mice exposed to HO/RA could be determined using C11-BODIPY staining in BALF as well as single-cell suspension from digested lung, and then determined in a flow cytometer.

The authors cannot make their conclusion that FTHKO protects ferroptosis in macrophages unless they are supported by the data generated from the above experiments.

Reviewer #5

(Remarks to the Author)
In the revised manuscript, the authors have appropriately addressed and responded to the comments raised by Reviewer #1. However, the reviewer has one remaining request.
Regarding C8: "Specific ferroptosis inhibitors in vivo will help support the conclusion", the authors tested both Lip-1 and Fer-1 in the HALI animal model. Unfortunately, only the data for Lip-1 have been included in the manuscript, while the results for

Fer-1 are mentioned only as “not shown.” Given that there are very few reports directly comparing the efficacy of Lip-1 and Fer-1 in the same animal model, even if the Fer-1 data were negative, presenting these results (at least as supplemental data) would be of value. It is well known that Fer-1 has much lower in vivo-stability compared with Lip-1, so limited efficacy of Fer-1 would be considered a reasonable outcome.

Version 2:

Reviewer comments:

Reviewer #4

(Remarks to the Author)
The authors have put some effort into responding to the raised concerns. However, they seemed to avoid the main question: whether there is more cell death in macrophages from FTH LysMKO mice. Meanwhile, the quality of the provided data needs improvement.
Study Specific Comments
1. The new Figure 2M indicates that only two wells of each group were included in the experiment. Moreover, the statistics did not reach significance, which compromises the conclusion.
2. No measurement of cell death in the newly provided data was performed, which contradicts the author’s claim that little death was induced and their observation in Figure 4M. It is important to have the cell death analysis via Sytox staining to support their hypothesis.
Minor Comments
The gating strategy in the panel Supplementary did not fully indicate the gating of CD11c+SiglecF+ AMs and SiglecFHi macrophages.
Strangely, the authors use LysM-Cre to study AM, which is not an ideal Cre for gene deletion in AM (https://doi.org/10.1038/s41423-022-00936-4). The KO efficiency in AM from FTH LysMKO mice is unexpectedly high (Supplementary Figure 5C). Please explain.

Version 3:

Reviewer comments:

Reviewer #4

(Remarks to the Author)
The new data added by the authors did not relieve my concern. Instead, they raised my attention to the data quality of flow cytometry. I suggest that they take this carefully, as these data are pivotal to support their core conclusion.
Study Specific Comments
1. The new Figure 4N should show the percentage of NIR+ AM and other myeloid cells (such as neutrophils and monocytes) in the gate of CD45 (the gating strategy in Supplementary Figure 9 for NIR should include CD45). The absolute number should also be plotted.
2 . Please provide the FMO control (cells stained with all the antibodies/dyes but NOT FerroOrange) for the FerroOrange staining (make a histogram plot merged with FMO and samples). This is because CD11c-PE-CY7 may interfere with the FerroOrange Channel.

Version 4:

Reviewer comments:

Reviewer #4

(Remarks to the Author)
The authors relieved my concerns about the flow cytometry.
They should explain the inconsistency between the description of Figure 4O in the result and the statistical indication in the plot.
One last thing, they should carefully discuss the main conclusion that Fth KO protects AM from HO, which seems to contrast with most FTH studies.

Open Access This Peer Review File is licensed under a Creative Commons Attribution 4.0 International License, which permits use, sharing, adaptation, distribution and reproduction in any medium or format, as long as you give appropriate credit to the original author(s) and the source, provide a link to the Creative Commons license, and indicate if changes were made.
In cases where reviewers are anonymous, credit should be given to 'Anonymous Referee' and the source.
The images or other third party material in this Peer Review File are included in the article's Creative Commons license, unless indicated otherwise in a credit line to the material. If material is not included in the article's Creative Commons license and your intended use is not permitted by statutory regulation or exceeds the permitted use, you will need to obtain permission directly from the copyright holder.
To view a copy of this license, visit https://creativecommons.org/licenses/by/4.0/

<u>Point-By-Point Responses to the Reviewers of Manuscript NCOMMS-24-66679</u>

We appreciate the constructive and insightful comments from the three Reviewers and the editors. We have performed substantial additional experiments leading to the generation of 35 new figure panels. We also provide further in-depth discussion based on the suggestions from the reviewers. We believe that these substantial revisions (denoted by red font in the revised manuscript) have improved the manuscript. A point-to-point response to each of the comments is enclosed below:

<u>Reviewer 1</u>

Acute respiratory distress syndrome (ARDS) is a type of respiratory failure, which could be induced by many direct or indirect causes. Previous studies have found some relations between ARDS and iron status or ferritin level. However, the role of extracellular ferritin is remains unclear. In this study, the authors demonstrated a correlation between the changes of ex-ferritin and the severity of ARDS. Furthermore, using Fth1-LysM and Fth1-Cd11c conditional knockout mice, the authors found Fth1 rich ex-ferritin promoted hyperoxia induced ARDS through neutrophil activation. This study has interesting. Some key issues that need to be addressed.

C1: The authors have observed that macrophage Fth1 deficiency has protective effect on hyperoxia-induced lung injury in mice and supposed that knockout Fth1 in macrophage reduced the Fth1 rich ex-ferritin level, and then inhibited neutrophil activation. Although this hypothesis is reasonable, how to exclude the direct effect of Fth1 knockout on macrophage function and viability.

We agree with Reviewer 1 that the direct effects of *Fth1* deletion on macrophage function and viability are very important. In our Manuscript we show that a loss of ferritin has no effect on cell viability at baseline but has a significant detrimental effect on macrophage viability in the setting of exogenous iron administration (**Figure 4E**). This is because FTH1-deficient macrophages store and oxidize free iron poorly (*See* Response to C3 below). To address the Reviewer's query regarding the effect of FTH1 loss on macrophage function we have performed new experiments to assess macrophage functional responses to bacteria. We show that in response to lipopolysaccharide (LPS), FTH1-deficient bone marrow-derived macrophages (BMDM) release lower amounts of IL-6 and TNF-α (**new panels Supplementary Figure 5F-G)**, suggesting that decreased iron storage in these macrophages may affect macrophage anti-bactericidal responses. This is in line with the findings of others who have shown that BMDMs isolated from *Fth1*$^{\Delta LysM}$ mice treated with interferon-gamma and lipopolysaccharide (LPS) have a lower activation of Nitric Oxide Synthase 2 (*Sci Rep. 2020 Feb 20;10(1):3061*). While others have shown that peritoneal macrophages from *Fth1*$^{\Delta LysM}$ mice have higher baseline production of IL-1β, IL-6, and TNF-α (*JCI Insight. 2021 Jul 8;6(13):e141760*), this does not translate to differences *in vivo* as *Fth1*^{LysM} mice respond similarly to i.p. infection with *Salmonella typhimurium* and *Fth1*$^{\Delta LysM}$ BMDM have normal phagocytic capabilities. We conclude that a loss in FTH1 in macrophages alters the baseline activity of these cells without altering baseline viability. However, how these cells respond to exogenous challenges will depend on the stimuli being used. We have added this new data to the Revised MS (See Page 11).

C2: Why only study macrophage Fth1? How the authors exclude other cell type ferritin function in models?

We appreciate Reviewer 1's concerns that other cell types may contribute to the extracellular ferritin pool observed in human acute respiratory distress syndrome (ARDS) and murine acute lung injury models. We chose to study macrophage ferritin as macrophages and monocytes were

the dominant cell types expressing ferritin heavy and light chain transcripts in the two human datasets we accessed (**Figure 1D, H, I**). To make this clearer, we have added additional labelling onto Figure 1D to highlight other cell types like epithelial cells and endothelial cells of the lung. In this Figure, alveolar type II epithelial cells (AT2 cells) as well as fibroblasts also show an abundance in expression of ferritin transcripts, albeit lower than the monocyte and macrophage populations. This implies that AT2 cells may also contribute to the extracellular ferritin pool in the lung in severe lung injury settings. To assess the contribution of AT2 FTH1 expression to the phenotype of *Fth1*$^{\Delta LysM}$ mice in our model, we isolated AT2 cells from these mice and found that *LysM*^{Cre} -driven FTH1 depletion did not change FTH1 protein expression in AT2 cells (**new panel Supplementary Figure 5E**). These findings suggest that while AT2 cell ferritin levels may likely be important, they are not contributing to our observed phenotypical or biological observations in our models using the *Fth1*$^{\Delta LysM}$ mice. We have added this new data and extra discussion points to the Revised MS (See Page 11).

C3: For Figure 4D, if the Fth1 is deficient, the intracellular labile iron level will be elevated. Actually, the elevated labile iron will promote ferroptosis. And the elevated Gpx4 and Slc7a11, and reduced Tfrc could be explained by compensate protective effect. But overall, the ferroptosis should be activated. How could it happen, as shown in Figure 4F, Fth1 deficiency inhibited RSL3 induced ferroptosis in mice BMDM?

Reviewer 1 is correct, and our original hypothesis was that a loss of FTH1 should lead to an increase in the labile iron pool and thus provide the free iron needed for the peroxidation of lipids associated with ferroptosis. However, to our surprise we observed the opposite effect whereby FTH1 deficient macrophages have lower total iron levels (**Figure 4C**) and were resistant to ferroptosis. Our conclusion is that the free iron generated by a loss of FTH1 has already been exported efficiently by the macrophages as these macrophages have higher levels of ferroportin, as well as decreased transferrin receptor to prevent additional free iron accumulation. This has been shown by others (*JCI Insight. 2021 Jul 8;6(13):e141760, Sci Rep. 2020 Feb 20;10(1):3061*) but to further support this hypothesis, we measured Fe^{2+} in control and *Fth1*$^{\Delta LysM}$ BMDMs following ferric ammonium citrate (FAC) treatment. We found that as expected, *Fth1*$^{\Delta LysM}$ BMDM have increased intracellular Fe^{2+} levels at baseline relative to *Fth1*$^{fl/fl}$ BMDM, which is exacerbated following iron loading (**new panel Figure 4D**). This helps to explain why *Fth1*$^{\Delta LysM}$ BMDM die with higher iron loads (**Figure 4E**). We elaborate on the relevance of this enhanced iron efflux in our Response to Reviewer 3 C20 below and have added this new data and extra discussion points to the Revised MS (See Page 14).

C4: Figure 5J, Ncoa4 deficiency inhibited ferritinophagy, thus elevated ferritin level, and then protected cell viability against RSL3 induced ferroptosis, which is consistent with previous studies, but contrary to the hypothesis in this study?

Reviewer 1 is correct in that others have shown that NCOA4 deficiency leads to a lack of bioavailable free iron and has been shown to be protective against ferroptosis. However, *ex vivo,* we observed that macrophages with NCOA4 deficiency display similar levels of Perls' staining (*not shown*) and similar total iron levels (**Supplementary Figure 12D**). To our surprise, mice with a loss of *Ncoa4* in alveolar macrophages developed more severe lung injury. We propose a hypothesis that the NCOA4 deficient macrophages fail to secrete extracellular ferritin in response to hyperoxia and may hence have less of a buffer system to bind excess extracellular iron as an explanation for this observation. Consistently we show that these mice have less extracellular ferritin in room air and hyperoxia conditions (**new panel Figure 6E**). To further test this theory, we have measured iron in the lavage fluid of the *Ncoa4*$^{\Delta Cd11c}$ mice in room air and hyperoxia conditions, finding that BALF iron levels in the *Ncoa4*$^{\Delta Cd11c}$ mice exposed to hyperoxia trended to be higher when compared to *Ncoa4*$^{fl/fl}$ mice exposed to hyperoxia (**new panel Supplementary**

Figure 12G). Although this is consistent with our hypothesis, there may also be additional reasons for why *Ncoa4*$^{\Delta Cd11c}$ mice are not protected against hyperoxia despite the macrophages themselves being resistant to ferroptosis *in vitro (***Fig 5L***).* An alternative hypothesis is that NCOA4 expression may also increase, independent of its role in iron metabolism. NCOA4 is also a coactivator that modulates the transcriptional activity of many nuclear receptors and exhibits non-ferritinophagic and iron-independent features such as promoting tumorigenesis and erythropoiesis, immunomodulation, regulating autophagy, and participating in DNA replication and mitosis (*Cell Death Discov. 2024 Jul 3;10(1):312*). We have added this new data and extra discussion points to the Revised MS (See Page 18).

C5: Figure 6N, the injury effect was mainly mediated by neutrophil activation. Actually, neutrophilic infiltration has multiple effects on inflammation and injury, including NETosis. How could we say ferroptosis is most important in neutrophil mediated lung injury?

Our schematic in **Figure 6N** is a working hypothesis model where we posit that neutrophils likely play an important role as a downstream target of extracellular ferritin secretion by macrophages. We agree with the Reviewer's comments that it may not be as simple as our model and that the role of neutrophils in lung injury is complex. We are currently in the process of developing models to specifically alter iron metabolism in neutrophils; however, this will require further extensive investigations out of the scope of the current study.

C6: All of the mice studies suggested higher Ftl1/Fth1 ratio have protective effect, but why the died individual in the COVID-19 study have higher FTL/FTH1 ratio compared with the survivors as shown in Figure 6M?

We were also surprised by these findings. However, like other stress responses, we believe that the increased FTL/FTH1 ratio represents an adaptive response, and that the clinical associations we observed are not causative. This is difficult to prove as we do not have the means to target FTH1 directly in human subjects. While there have been numerous studies which associate high serum ferritin levels with inflammatory diseases, much of the studies involving the pathogenic mechanisms of FTH1 and FTL subunits have been *in vitro*. The high FTL/FTH1 in COVID-19 here should be interpreted as an association that will require further extensive investigation out of the scope of the current manuscript. We have added a new discussion point to our revised MS discussing this limitation (See page 24).

C7: The authors have found alveolar injury and alveolar epithelial cell death as shown in Figure 2A and Figure 3G. But only macrophage was studied in vitro, whereas no epithelial cell was studied. Similarly, no epithelial cell information is showed in the overview schematic of the study in Figure 6N. Actually, alveolar epithelial cell injury is more important than macrophage injury in ARDS. Macrophage could be a causer or mediator, but not the target cell.

We agree with Reviewer 1 that acute lung epithelial injury is fundamental to ARDS pathobiology. However, we chose to study macrophages in this study as macrophages and monocytes had the highest abundance of ferritin transcripts in the human scRNA-seq data sets. There was also significant evidence in the literature that macrophages were the source of ferritin in hyperferritinemic syndromes like macrophage activation syndrome and COVID-19 (*Autoimmun Rev. 2020 Jul;19(7):102573.*). While AT2 cells also appear to modulate their ferritin in ARDS (See Response to Reviewer 1 C2), we focused on the macrophages for this study. However, we believe that iron- and ferritin-mediated crosstalk between these two cell types is understudied in ARDS and other respiratory diseases, and this question is therefore currently under active investigation

in our laboratory. We have now added a new discussion point to our revised manuscript regarding the important role of AT2 cells in ARDS and macrophage crosstalk (See Page 25).

C8: Specific ferroptosis inhibitors in vivo will help support the conclusion.

We agree with Reviewer 1 and have performed substantial new experiments using two ferroptosis inhibitors, ferrostatin-1 (Fer-1) and liproxstatin-1 (Lip-1) *in vivo*. In our revised MS we show that prophylactic intraperitoneal administration of Lip-1 (10 mg/kg) does not protect wildtype C57BL/6 mice from hyperoxia-induced lung injury in terms of survival (**new panel Supplementary Figure 9A**), weight loss (**new panel Supplementary Figure 9B**), or indirect lung injury markers (**new panel Figure 5A-C**). However, there is a trend for decreased BAL cell counts in mice receiving Lip-1 (**new panel Figure 5D**). We repeated these experiments twice and performed two experiments using Fer-1 (*not shown*) to confirm these findings. It is not yet clear to us why these ferroptosis inhibitors failed to alter survival of wildtype mice to hyperoxia. Both Lip-1 and Fer-1 serve as radical-trapping antioxidants and protect against ferroptotic cell death in both *in vitro* and *in vivo* models (*ACS Cent Sci. 2017 Mar 22;3(3):232-243)*. Most of these models work best when these agents are delivered directly into the target tissue, and it is unknown whether intraperitoneal delivery adequately reaches distant cells, such as airway immune cells in our hyperoxia model. Additionally, these drugs could only be delivered prophylactically up to 48 hours of hyperoxia, as from our experience taking the animals out of the hyperoxia chamber after this timepoint exacerbates organ injury due to the drastic change in ambient oxygen concentration. It is possible that continued administration of these drugs throughout the exposure may have beneficial effects, but due to 3R animal practices we are unable to carry out these types of experiments. Aside from possible dosing and pharmacokinetic problems, neither Lip-1 and Fer-1 target kept anti-ferroptotic proteins such as GPX4 or SLC7A11, and they do not modulate intracellular iron handling, to our knowledge. We have added the Lip-1 data to the revised MS (See Page 16).

C9: Lack of genetic models to support ferroptosis working model.

Given the important role of ferritin and NCOA4 in the regulation of ferroptosis, we had used these two genetic models to target ferroptosis indirectly by altering available iron levels. Obtaining other non-iron related genetic models deficient in classic ferroptosis regulators like GPX4 (*Cell. 2018 Jan 25;172(3):409-422.e21; J Exp Med. 2022 Nov 7;219(11):e20220504*) has been challenging with the strain only available for cryo-recovery in the Jackson Laboratory, which entails 12 weeks to get the mice and another 4-6 months to cross and back-cross to generate a stable cell-specific lineage. Given the ample *in vitro* evidence connecting ferritin and NCOA4 and ferroptosis (*Autophagy. 2023 Jul;19(7):2062-2077; Adv Exp Med Biol. 2021;1301:41-5*7), we believe that genetically targeting ferritin and NCOA4 will suffice for this study, but we agree with Reviewer 1's comment and have added a new discussion point proposing to utilize other ferroptosis mice for future studies (See page 23).

<u>Reviewer 2</u>

Summary: This study by Zhang et al investigating the role of ferritin in macrophages and monocytes and induction of ARDS in mice. Authors start the paper with data from COVID-19 patients and then move to the HALI model of ARDS. This Reviewer questions the HALI model of ARDS and relevance to COVID-19 patients (see below). The manuscript reads well. Authors developed cutting edge mouse models including multiple conditional deletions in myeloid cells and macrophages, which is relevant to the study. This Reviewer however questions the lack of cell sorting in some experiments and in general the lack of Immunology readouts such as flow cytometry, for instance for all experiments

investigating cell numbers. Some of the RNAseq pathway analysis is weak, with only one gene in some highlighted pathways. Please see below for more detailed comments.

C1: Not all COVID-19 patients develop ARDS. Therefore, what are the markers used by the authors, if any, to conclude that the data set is only focusing on COVID-19 ARDS? If no data is available, the text should read that the data set is on COVID-19 patients, not COVID-19 ARDS. Lines 97, 116, 120 etc are examples. This issue is throughout the manuscript.

Thank you for highlighting this important caveat to the human data sets used in this study (**Figure 1**). This is particularly relevant for the scRNA-seq data sets used in this study. Figure 1D-G contains single cell data from patients who died from COVID-19, whereas Figure 1H-J includes data from individuals with less severe COVID-19. However, the New York Presbyterian Data set that contained serum ferritin measurements for over 383 subjects who were intubated for severe hypoxemic respiratory failure with bilateral lung infiltrates, meeting the Berlin criteria for ARDS diagnosis. Therefore, the data in Figure 1A-C are from subjects with COVID-19 ARDS who were all intubated and on mechanical ventilation and met the Berlin criteria for ARDS diagnosis. We have clarified this in the text.

C2: Related to these comments: Line 165: is COVID-19 and "human ARDS" interchangeable?

We apologize for the lack of clarity in our wording and have corrected this throughout the MS. See Response to Reviewer 2 C1 above. As the Reviewer correctly states, not all subjects with COVID-19 develop ARDS, and therefore COVID-19 and human ARDS are not interchangeable.

C3: Figure 1C. This shows baseline Ferritin, but are these really baseline? The levels of "baseline" ferritin are not available (pre-infection by COVID-19) and since inflammation affects ferritin and iron metabolism in general (inflammation-anemia), these values are most probably not baseline. They reflect ferritin upon entry in ICU, which is probably very variable between patients. The authors should make this clear to the readers.

We thank the Reviewer for pointing out this distinction and have revised the wording in the manuscript to "initial ferritin levels upon ICU admission".

C4: Figure D. Not clear why only macrophages and monocytes are labeled. All cells should be labelled.

We apologize for this oversight. We have added a new fully annotated UMAP in the revised MS (*See* **Figure 1D**).

C5: Figure 1J. Could this downregulation be seen in the lungs? (ie panel E)? Since panel J is blood, it is hard to compare with lung monocytes. What are the dynamics of FTH1 expression in macrophages with disease severity?

This is an interesting question from Reviewer 2 but one that is difficult to answer in human data sets, since most -omic studies of lung tissue are from lung ex-plants or deceased subjects, the scRNA-Seq data from Figure 1D-G in this study included. Regarding the kinetics of ferritin expression over time, we show in our model system that extracellular ferritin levels rise in the serum and BALF in a time dependent manner (72h, 96h) with hyperoxia exposure with wildtype mice starting to die at approximately 100 hours. We also show that expression of *Fth1* and *Ftl* transcripts increase at 72h in BAL cells. At the 96h timepoint, the influx of neutrophils into the lavage fluid made it hard to assess the expression of these transcripts. However, in the Revised Manuscript we have performed new experiments to show that *Fth1* and *Ftl* expression are also

significantly higher in flow cytometry-sorted alveolar macrophages (Cd11c$^+$Siglec-F$^+$) at the 96h timepoint (**new panels Figure 2G-H**). These findings are consistent with the rise in extracellular ferritin levels and collectively demonstrate that macrophage ferritin expression and extracellular ferritin levels rise in a time dependent manner in response to hyperoxia. We have added this new data and extra discussion points to the Revised MS (See Page 9).

C6: What is the rationale for choosing HALI over other models. Since authors emphasize the relationship between COVID-19 and ARDS. There are other models that replicate better viral-induced ARDS than HALI.

As a respiratory biology laboratory, we wanted to focus on the "sterile" ARDS/acute lung injury component of the story, and utilized COVID-19 as an illustrative case since it was an ARDS epidemic. In our Revised Manuscript we have also performed new *in vivo* murine experiments using an additional "sterile" ARDS model, namely a ventilator-induced lung injury (VILI) model to show that our findings apply more broadly to ARDS in general rather than a specific etiology of high-oxygen associated ARDS. Specifically, we now show that BALF ferritin is also increased following lung injury induced by large tidal volumes (**new panel Figure 2E**).

To increase the generalizability of our clinical observations, we also further queried our NYP database and compared serum ferritin levels within 3 days of ICU admission between patients with and without ARDS and found that subjects with ARDS had higher levels than those without ARDS (**new panel Supplementary Figure 1C**). Additionally, we have now added a new clinical cohort (*Nat Immunol*. 2022 Jun;23(6):927-939) in which we assessed the protein expression of FTH1 and FTL in blood monocytes of individuals meeting the Berlin definition for ARDS (n= 4) compared to those of healthy controls (n=3) via proteomic profiling. We show that FTL protein levels are significantly higher in the monocytes of subjects with clinically diagnosed ARDS when compared to controls, with a trend for an increase in FTH1 as well (**new panel Supplementary Figure 2C-D**).

To address and relate these findings to bacterial or viral associated ARDS, we had included data using an LPS ALI model showing that lung macrophages in this model have higher *Fth1* expression (**Supplementary Figure 3D**). In the Revised Manuscript, we have also performed new experiments showing that BALF ferritin is higher in an influenza-induced and bacterial (*Streptococcus pneumoniae*)-induced lung injury model (**new panels Figure 2F**, **Supplementary Figure 3A**). To relate these findings to a viral-ARDS model, as suggested by the Reviewer, in the Revised Manuscript, we have also performed new experiments showing that BALF ferritin is increased in a viral (influenza)-induced lung injury model. Furthermore, myeloid FTH1 depletion is protective against influenza-induced lung injury (**new panels Supplementary Figure 6A-B**), suggesting that the phenotype we observed is not limited to sterile ARDS models. Taken together, these additional pieces of data indicate that ferritin upregulation is consistently seen in ARDS of various etiologies, supporting the rationale for our mechanistic murine studies. We have added this new data and extra discussion points to the Revised MS (See page 10 and page 12).

C7: Line 168. Please show BAL cellularity by flow cytometry. It is true that macrophages are present in excess, but monocytes and neutrophils should also be well represented, as per the author's definition of clinical readouts of ARDS. This is a requirement to interpret Figure 2E-G as it is hard to believe these BALs are only macrophages.

In the revised manuscript we have performed new *in vivo* experiments to analyze the BALF from mice exposed to hyperoxia by flow cytometry. Supporting our BALF cell differentials by histology, there are decreased AM counts in *Fth1*$^{fl/fl}$ mice relative to *Fth1*$^{\Delta LysM}$ mice (**new panels Figure 3M-N**) which is consistent with a prior published study which decreased proliferation of lung

monocytes and AM at 48 hours, prior to the recruitment of neutrophils, in a murine hyperoxia model (*Sci Rep. 2020 Mar 13;10(1):4677*).

C12: Figure 3I-K. It would be relevant to include flow cytometry analysis of these myeloid cells. Flow cytometry would also allow to quantify the cellular iron in each population.

As part of our flow cytometry study (see response to C11 above), we also evaluated AM TFRC expression as a surrogate marker of cellular iron concentration. Consistent with our BMDM RNA-Seq data, AM TFRC expression is induced with hyperoxia in $Fth1^{fl/fl}$ mice, an effect which is blunted in $Fth1^{\Delta LysM}$ mice. This suggests that there is increased AM iron uptake in hyperoxia, which has been shown to be detrimental to AM health in prior studies (*Am J Respir Crit Care Med. 1999 Jan;159(1):100-6*) and that does not occur in the $Fth1^{\Delta LysM}$ mice.

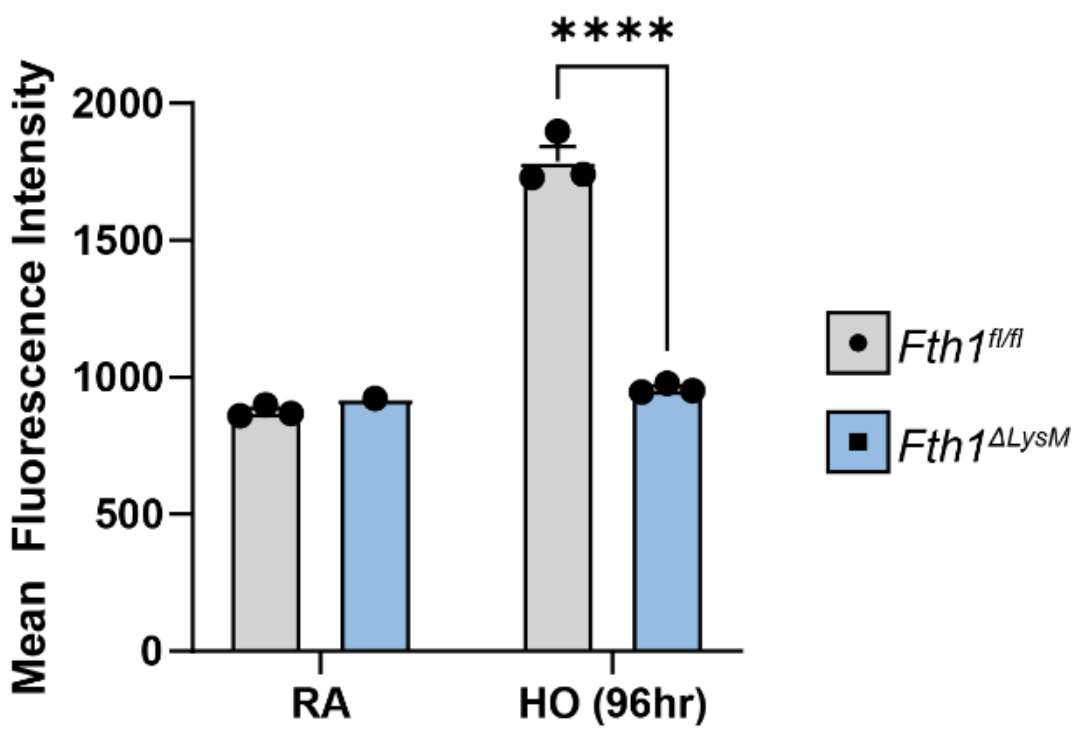

Figure 1. AM FTH1 depletion decreases AM TFRC expression in hyperoxia. CD11c$^+$SiglecF$^+$ AM TFRC geometric mean fluorescence in $Fth1^{fl/fl}$ and $Fth1^{\Delta LysM}$ mice. P-values by analysis of variance (ANOVA) with Šídák's correction for multiple comparisons, **** $p<0.0001$.

C8: Related to the previous point. Authors should sort macrophages from the BAL prior qPCR. Since different results were seen using in LPS model of ARDS.

We have performed new experiments by sorting for Cd11c$^+$Siglec-F$^+$ alveolar macrophages from room air mice and mice exposed to 96 hours of hyperoxia and then isolating RNA from these cells and performing qPCR for *Fth1* and *Ftl* mRNA expression. Sorted AMs from hyperoxia mice have increased *Fth1* and *Ftl1* expression compared to those from RA mice (**new panels Figure 2G-H**), data which is now complimentary to our initial findings using unsorted BALF cells (now in **Supplementary Figure 2B-C**). We have added this new data to the Revised MS (See Page 10).

C9: Line 197. Related to point above, as long as there is no proof that the BAL cells are ALL AM, it is not possible to conclude that macrophages upregulated ferritin at the transcriptomic level.

See Response to Reviewer 2 C8 above.

C10: Figure 2L. Please explain why "response to bacterium" is upregulated in HALI, which does not include bacterial infection. What genes does this pathway take into account? Also, the size of the " No of genes" legend does not match the actual plots. Overall, there seems to be a very limited number of genes in these pathways, especially panel M. Are these enough to claim they are enriched?

We apologize for the confusion related to our RNA-Seq analysis. We have re-performed the analysis using Enrichr and have now included **new panels Figure 2I, Figure 4B,** and **Supplementary Figure 4B.** We have added this new data to the Revised MS (See Page 10 and 14).

C11: Figure 3B. How do the authors explain the increased protein concentration in the cre group at 72h, and a decrease 24h after? Is this physiological?

We wanted to clarify the Reviewer's comment, which we interpreted to mean that $Fth1^{\Delta LysM}$ mice had higher BALF protein at 72h and then lower at 96h; this observation is also made by Reviewer

3 (C15). We have not uncovered the reason behind this change in phenotype between the 72h and 96h timepoints, but it may have to do with the recruitment of neutrophils into the airspace, which also occurs earlier in *Fth1*$^{\Delta LysM}$ mice in hyperoxia. Regardless of the biological reason, for the purposes of communicating our findings we have made the decision to just show the 96h timepoint, as requested by Reviewer 3 (C15), while we continue to explore the immune cell dynamics that may explain these findings.

C13: About BMDMs in Figure 1 and Figure 3. Authors derive BMDM from the bone marrow of mice that went through induction of ALI. How physiologically relevant are these BMDMs compared to macrophages that are actually present at the site of inflammation in the lungs? It would be more relevant to perform RNAseq on AMs isolated from the lungs of mice with ARDS.

We agree with Reviewer 2 that carrying out bulk-RNA-Seq on isolated AMs would have been more relevant. However we did not obtain good enough RNA yields from isolated AMs for this approach and we lacked resources for scRNA-Seq approaches. We had performed bulk- RNA-Seq on BMDMs which we hypothesised would be relevant to capture the infiltrating pathogenic monocytes that may be recruited during hyperoxia exposure (*J Exp Med (2017) 214, 2387-2404, J Exp Med (2020) 217 (3): e20191236, Development (2016) 143, 1318-1327*). As has been shown in prior studies that inflammatory responses often shape bone marrow progenitors (*Nat Rev Immunol. 2020 Jun;20(6):375-388*) we were confident that we would discover differences between the experimental groups, and that these differences would be representative of similar changes in the lung. However, to address Reviewer 2's concerns we have performed new experiments (See Response to C8 Reviewer 2 above) to show that sorted AMs from hyperoxia mice have increased *Fth1* and *Ftl1* expression compared to those from RA mice (**new panels Figure 2G-H**). We have added this new data to the Revised MS (See Page 10).

C14: Figure 4B. How many genes are in the "Response to L-ascorbic acid" pathway. Is this enough to claim that the pathway is "enriched"? Same question for the ferroptosis pathway in Figure 4C. Based on the legend it corresponds to one gene – not enough to claim the pathway is enriched. Most of the pathways in Figure 4C are representing 1-3 genes in each pathway, this is a very poor analysis.

See Response to Reviewer 2 C9 above.

C15: Minor comments.
Line 203. "robustly" may be an overstatement.
Lines 414-415. The word "that" is repeated

We have made these changes to the manuscript.

Reviewer 3

Zhang et al. investigated the role of macrophage ferritin (particularly FTH1) as a potential factor modulating immune response and disease severity in different models of acute respiratory distress syndrome (ARDS). The study offers intriguing insights into how iron metabolism interacts with the immune system. It supports previous suggestions in the literature about the potential immunomodulatory role of ferritin, a topic that has not been thoroughly explored until now.

To study their hypothesis, the Authors performed a series of extensive and well-designed experiments, starting from cohorts of COVID-19 patients and subsequently moving toward hyperoxia-induced acute lung injury (HALI) mouse models.

The study is valuable and characterized by experiments carried out with validated and reproducible techniques. Nevertheless, this complex paper includes some inconsistencies and contradictions (also regarding certain methodological choices) that require clarification and more robust arguments before being considered for publication.

Below, we provide a list of general and study-specific comments to improve the paper and its readability.

General Comments
C1: Text needs English editing, as there are repetitions, typos, etc.
e.g. Line 82-83: "in the setting of ARDS" and "in individuals with ARDS" appear a repetition in the same sentence
e.g. Line 387: "in" is repeated
e.g. Line 446: "that" should be deleted

We apologise for these oversights; we have corrected grammatical errors throughout.

C2: The manuscript should be ameliorated in terms of readability and clarity. There are some redundant parts and some inconsistencies throughout the text.
e.g. please uniform PBMC/PBMCs; BAL/BALF; BMDM/BMDMs; HALI/hyperoxia-induced ALI across the whole manuscript

We have corrected these inconsistencies throughout.

C3: Results section: this section should be amended, as it is full of "discussion-like comments." The results section should contain just the description of the results without hypothesis on possible explanatory mechanisms or speculation on causes/effects.

We have refined the results sections to limit interpretation where possible.

C4: The conclusion section should be renamed as "discussion".

Corrected.

C5: Some of the speculations/hypotheses made by the authors are too assertive and should be mitigated.
e.g. Line 87-88: the sentence "we hypothesized that the main source for ex-ferritin in critical illness is the macrophage" refers to a piece of data that is at least partially already demonstrated, so the authors could reformulate it accordingly.

This has been rewritten.

C6.The paper focuses on the role of the ferritin H-subunit. Did the authors assess the levels of ferritin-bound iron? This data could provide valuable insights into whether the observed effects were due to ferritin itself or are mediated by the iron it carries.

Ferritin bound iron measurements are technically difficult and to the best of our knowledge can only be achieved by carrying out non-denaturing polyacrylamide gel electrophoresis coupled with Prussian blue staining in-gel (*Methods Mol Biol. 2023:2671:121-134*). We attempted to carry out this technique in our human samples, but we could not obtain any Perls' staining on these gels to quantify anything. As these were human samples the availability of starting material was limited

and hence, we were unable to repeat this approach. Furthermore, spectrophotometric quantification of the total amount of iron accumulated inside ferritin (via acid/heat-based denaturation trailed by ferrozine assay or similar) can only be carried out on isolated mineralized ferritin which is technically challenging in human samples and in cell types like macrophages of relatively low abundance. We hope that our parallel measurements of ferritin transcripts, protein and total iron levels in the same samples (humans and mice) will suffice and have provided new data to the revised manuscript to show that FTL:FTH ratios do not correlate with iron measurements in the same samples in our human cohort (**new panel Supplementary Figure 14C**). We have added this new data and extra discussion points to the Revised MS.

C7. A key player in iron metabolism, that is, hepcidin, has not been considered in the experimental design. Hepcidin is strongly regulated by pro-inflammatory cytokines and plays a pivotal role in orchestrating iron distribution between intracellular and extracellular (plasma) iron levels during acute inflammation. Beyond the systemic effects of liver-derived hepcidin, this hormone is also produced locally by a variety of cells, including alveolar macrophages. Why did the authors not measure, for example, hepcidin levels in the bronchoalveolar lavage fluid (BALF) and in the serum of both patients and mouse models? This limitation should be acknowledged and discussed.

We agree with Reviewer 3 that assessing hepcidin levels is important for the regulation of iron during acute inflammation. We therefore performed new experiments measuring hepcidin levels in the serum and BALF of mice exposed to hyperoxia. 72h of hyperoxia exposure resulted in increased serum hepcidin, with *Fth1*$^{\Delta LysM}$ mice having even higher levels compared to *Fth1*$^{fl/fl}$ mice (new panel (**new panel Supplementary Figure 10A**). Interestingly, the same phenomenon is observed with BALF hepcidin (**new panel Supplementary Figure 10B**). These findings are consistent with work carried out by the lab of Lukas Kuhn who previously deleted ferritin H expression in adult mice using a polyIC inducible Mx gene promoter (*Hepatology. 2009 Sep;50(3):852-60*) and showed that these mice had significantly higher hepcidin expression in the liver. These data suggest that myeloid-ferritin depletion alters systemic iron handling, potentially via the hepcidin-ferroportin axis, and thus this data has been added to the manuscript (See page 15/16).

Study Specific Comments

Results:

C8. Paragraph “1” Serum ferritin trajectory associates with increased mortality in COVID-19 ARDS.

Line 101: the authors talk about 383 subjects, while in Lines 108 and 110: the IOT/deceased patients are 119, the IOT/survivors are 213 (total 332); in Suppl Tab 1: the IOT/deceased patients are 157, the IOT/survivors 249 (total 406). Could the authors clarify these discrepancies?

These differences in numbers reflect the changes in the cohort over time and data missingness. We carefully define the ferritin variables we use, and subjects who do not have ferritin data within the specified period were excluded from the analyses. This is particularly relevant with delta ferritin, because some subjects may not have a second ferritin value measured to calculate the difference. We have made sure that this is clear in the methods and figure legends of the MS.

C9. Suppl Tab 1: there are three columns; could the authors clarify what the p refers to? only to the comparison between the first two columns or between all of them?

Our apologies for the lack of clarity regarding the p values; the p-values represent global p-values (i.e., a comparison of all three groups). Pearson’s Chi-squared test was used to compare

categorical variables, and Kruskal-Wallis rank sum test was used to compare continuous variables. We have made sure that this is clear in the caption to Supplementary Table 1.

C10. Lines 116-117: the Authors state that "ex-ferritin levels rise with increasing ARDS disease severity in COVID-ARDS and associate with worse clinical outcomes"; however, data in Suppl Tab 1 seems to highlight that IOT/deceased patients were older and with a higher prevalence of some comorbidities; could the authors comment on this aspect? Additionally, was ferritin delta an independent predictor of mortality after adjusting for age and comorbidities?

In our model, on average, each 100 ng/mL increase in delta ferritin was associated with a 1.12% increase in the odds of 28-day mortality, adjusting for age and sex (aOR=1.012, 95% CI 1.002, 1.022, p=0.0163) or adjusting for age, sex, hypertension, diabetes, active cancer, CAD, and BMI (aOR=1.012, 95% CI 1.003, 1.022, p=0.0139). We have added this new data to the Revised MS (See Page 7).

C11. Paragraph "2" FTH1 and FTL expression are enriched in monocytes and lung macrophages in individuals with ARDS
Lines 146-147: the authors affirm that "ferritin transcripts are highly enriched in myeloid cells within the lungs and peripheral blood of critically ill COVID-19 patients". However, this seems to contradict the observation that the expression of FTH1 and FTL in monocytes of subjects with severe/lethal COVID19 is reduced. Could the authors please clarify this point? Please consider modifying the title of the paragraph accordingly.

We apologize for the lack of clarity on this point. When we use the word "enrichment" we meant that the transcript levels of *FTH1* and *FTL* are much higher in certain cell types, when compared to other cell types. e.g. high in macrophages /monocytes but low in endothelial cells. Reviewer 3 is correct in that the directionality in change of these transcripts is different between the lung macrophages and the monocytes. To resolve this issue we have replaced the word "enrichment" with "abundance" or "abundant" so that the directionality of change is not implied.

C12. Paragraph "3" Hyperoxia-induced ARDS modulates macrophage ferritin expression and extracellular ferritin
Lines 171-173: In these lines, the authors mention a different mouse model with acute lung injury, which is not mentioned again in the text. I wonder if the authors intended to emphasize a difference in ferritin expression, specifically in an inflammatory lung injury model rather than in a hyperoxia model. However, this intention is not clear in the current text. I suggest the authors provide justification or a commentary on this or consider omitting this data.

Please see Response to C6 Reviewer 2.

C13. Lines 196-197: the authors state that "These findings indicate that hyperoxia significantly elevates FTH1 expression and to a lesser extent FTL, in AMs (alveolar macrophages) and in monocyte-derived macrophages". However, in the text, it appears that FTL expression is increased only in BALF macrophages, while it remains unchanged in bone marrow-derived cells. Can the authors clarify this point?

We apologize for the lack of clarity regarding this point. Reviewer 3 is correct that *Fth1* and *Ftl* expression are higher in human and murine lung AMs in ARDS, when compared to controls, whereas in human blood monocytes and murine BMDMs in ARDS there is a trend towards lower expression of these transcripts when compared to controls. We had hypothesized that in response to severe lung injury, such as that induced by hyperoxia, resident lung macrophage populations

decline and secrete chemokines and cytokines that recruit and activate monocytes and neutrophils from the peripheral circulation as is observed in other lung injury models (*J Exp Med (2017) 214, 2387-2404, J Exp Med (2020) 217 (3): e20191236, Development (2016) 143, 1318-1327*). These infiltrating monocytes differentiate into macrophages helping to sustain the resident macrophage pool. We had hypothesized that ferritin expression is low in the circulating monocytes and upon entry into the lung microenvironment ferritin expression is induced. This hypothesis is supported by recent observations that *Fth1* expression has been used as a reporter of the differentiation of recruited monocytes into macrophages in the lung during LPS-induced lung inflammation (*J Immunol (2024) 213 (10): 1508–1515*). However, we have now included new data to show that ferritin protein expression may also be higher in blood monocytes of subjects with ARDS. Using an ARDS clinical cohort (*Nat Immunol*. 2022 Jun;23(6):927-939) we assessed the protein expression of FTH1 and FTL in blood monocytes of individuals with ARDS (n= 4) compared to those of healthy controls (n=3) via proteomic profiling. We show that FTL protein levels are significantly higher in the monocytes of subjects with clinically diagnosed ARDS when compared to controls, with a trend for an increase in FTH1 as well (**new panel Supplementary Figure 2C-D**). In iron biology, measuring ferritin protein levels is essential because they directly indicate cellular iron storage and regulation, while transcript levels which may not accurately reflect the functional protein due to post-transcriptional modifications. Therefore, we also assayed for FTH1 protein levels in BMDMs differentiated from bone marrow cells from room air mice and mice exposed to hyperoxia and found that similarly BMDM FTH1 protein is increased in hyperoxia (**new panel Figure 2L**). While this suggests that monocytes as well as macrophages may upregulate ferritin in response to hyperoxia/ARDS, to prove that infiltrating monocytes are the source of this high ferritin would require extensive lineage tracing of ferritin in monocytes as well as parabiosis models, which are unfortunately outside the scope of this study. To ensure we have addressed Reviewer 3's comment we have added this new data to the Revised MS (See Page 11).

C14. When the authors mention monocyte-derived macrophages, are they referring to BMDMs?

We have amended the text when feasible to clarify that we are referring to BMDMs when we mention monocyte-derived macrophages.

C15. Paragraph "5" Macrophage FTH1 depletion alters the cellular inflammatory response to hyperoxia
Lines 239-241: the authors state that "This early onset of neutrophil infiltration in the Fth1ΔLysM mice at 72 hours mirrored higher BALF protein (p=0.04) and a trend for higher BALF IgM at 72 hours compared to Fth1fl/fl mice". This sentence refers to observations made at 72 hours and seems to contradict the information presented in the previous paragraph (Lines 214-216), where proteins and IgM concentrations in the BALF were measured at 96 hours. The authors should strive for consistency in reporting time points, as discrepancies between 72 and 96 hours may confuse the readers. In this case, omitting the data collected at 72 hours may be advisable.

Thank you for this important observation. We have removed most of the 72h timepoints to improve clarity and focus the MS on the more severe endpoints in these models. However, we chose to keep the time-kinetics of cellular immune cell infiltrates in the BALF fluid to highlight the time course of monocyte/macrophage and neutrophil recruitment into the lung.

C16. Lines 252-254: the authors state that "the lower airway macrophage and monocyte numbers in control Fth1fl/fl relative to that of Fth1ΔLysM mice may not be due to the

absence of chemotactic signals". Could the authors clarify why they also mention a smaller number of monocytes?

We have corrected this error and removed the word "monocytes".

C17. Paragraph "6" FTH1 depletion protects macrophages from ferroptosis.
Lines 267-275: It is unclear whether the hyperoxia exposure lasted for 72 or 96 hours and what the difference is between mice evaluated at baseline versus those in room air. Could the authors clarify these aspects in more detail?

We have clarified this paragraph, and all the data presented are comparisons between the macrophages from the respective mice at the 96 hour hyperoxia timepoint.

C18. Line 279: Ferritin expression should decrease when cellular iron content declines. Could the authors comment on the increase of Ftl expression in FTH1-depleted (iron deficient) BMDMs?

Reviewer 3 is correct we had expected that if cellular iron content declines, then both FTH1 and FTL expression should decrease. However we did not observe this and observed a striking increase in *Ftl* transcripts upon *Fth1* loss. However, it is not surprising that there would be a compensatory rise in *Ftl* and this has been observed by other groups: The Kuhn lab observed that livers deficient in FTH1 showed increased immunohistochemical staining for FTL, which they speculated was likely due to IRP1 and IRP2 inactivation by iron liberated upon ferritin H degradation (*Hepatology. 2009 Sep;50(3):852-60*). Loss of FTH1 in macrophages results in higher FTL protein levels, as well as lower iron levels (*Diabetologia (2020*) *63, 1588–1602, JCI Insight. 2021 Jul 8;6(13):e141760, Front Immunol. 2019 Feb 4:10:131.*). The Goldfarb lab have also recently published a conference abstract with observations that when they target *Fth1* in erythroid cells and erythroid-associated macrophages with *EpoR-Cre,* red blood cells showed a marked increase in FTL on immunoblot (> 10-fold) along with a ~2-fold increase in antioxidant factors, which they suggest may be a compensatory mechanism to mitigate ferroptosis (https://doi.org/10.1182/blood-2024-205194). We therefore hypothesise that FTL increases upon loss of FTH1 as a compensatory mechanism to protect the cells from free iron and in turn ferroptotic cell death. We have made sure to add a discussion point to the Revised Manuscript highlighting these papers.

C19. Paragraph "7", The intracellular iron chelator deferiprone exacerbates hyperoxia-induced lung injury
Lines 322-324: these lines seem to report the same data already mentioned in lines 279-280 about the decrease in Tfrc and the increase in ferroportin expression

We apologise for this duplication and have removed this superfluous text.

C20. Lines 324-327: the authors state that "This hypothesis is further supported by our observation that Fth1ΔLysM BMDMs are sensitive to exogenous iron as iron added in the form of ferric ammonium citrate (FAC) rapidly decreases their viability relative to Fth1fl/fl cells over 24 hours". This observation seems to contradict the fact that Fth1ΔLysMBMDMs cells acquire less iron and release more iron than the wild-type model. Could the author explain why Fth1ΔLysMBMDMs, despite acquiring less iron and releasing more, experience a greater viability reduction after FAC exposure than the normal Fth1fl/fl model?

We understand Reviewer 3's concerns. We hypothesized that these cells acquire less iron and export more iron as they do not have the capacity to store iron in an inert non-toxic state. Without

ferritin, free iron is toxic to the cell. We posit that FTH1 deficient cells have adapted to remove any iron to avoid this toxicity. However, if exogenous iron is provided in abundance such as is the case with FAC administration, these cells are overwhelmed and the excess iron becomes toxic. This is consistent with the findings of others, who have shown that H-ferritin-deficient macrophages have a higher sensitivity to iron-induced toxicity with a lower intracellular iron accumulation but a higher production of reactive oxygen species (*Sci Rep. 2020 Feb 20;10(1):3061, JCI Insight. 2021 Jul 8;6(13):e141760*). Similarly, embryonic fibroblasts from mice with a global loss of FTH1 exposed to ferric ammonium citrate are 30-fold more sensitive to iron associated toxicity, again linked to reactive oxygen species development and mitochondrial damage. These mice when fed a high iron diet also develop severe liver damage (*Hepatology. 2009 Sep;50(3):852-60*). Please also see our Response to C3 Reviewer 1 where we provide new data to show that FTH1-deficient BMDMs have increased intracellular Fe^{2+} levels following iron loading immediately (**new panel Figure 4D**), but while this free Fe^{2+} decreases rapidly in *Fth1$^{fl/fl}$* BMDMs as it is converted to Fe3+ and stored in the ferritin nanocage, it sustains in *Fth1$^{\Delta LysM}$* BMDMs as it cannot be stored. We have added this new data and extra discussion points to the Revised Manuscript highlighting these papers.

C21. Lines 339-337: To effectively test the study hypothesis, the authors should have established selective iron depletion in macrophages. The use of systemic deferiprone (DFP) leads to a state of global iron deficiency, impacting other organs and tissues as well. Previous studies have indicated that iron deficiency and iron deficiency anemia can exacerbate certain lung diseases. Did the authors assess levels of hemoglobin (Hb) and ferritin in the murine model treated with DFP?

We agree with Reviewer 3 and thus chose to test our hypothesis by selectively depleting ferritin heavy chain and NCOA4 in macrophages using two sperate Cre systems. Intratracheal administration of DFP to the lungs would have been a more direct approach, however given the extent of epithelial cell damage from hyperoxia we were concerned the animals would not survive a second insult directly to the lung. In our model, we utilized only 1 week of deferiprone treatment which we now show does not result in iron deficiency anemia. Specifically, we have performed new experiments to show that 1 week of deferiprone administration does not alter Hb *in vivo* in the room air mice (**new panel Supplementary Figure 11B**); a prior study showed that a short course (7 day) of oral deferiprone does not alter serum ferritin levels (*J Ayub Med Coll Abbottabad. 2024 Jan-Mar;36(1):35-39*). We have added this new data and extra discussion points to the Revised MS (See Page 16).

C22. Paragraph "8" Impaired macrophage ferritin recycling exacerbates hyperoxia-induced ALI
Lines 364-366: I have some concerns regarding the authors' statement and the mouse model used to test their hypothesis. The authors claim that FTH1-depleted mice are protected from hyperoxia damage (paragraph 1) and that FTH1 depletion protects macrophages from ferroptosis (paragraph 2). However, they also report an increase in NCOA4 levels, while the literature suggests that the loss of NCOA4 is protective against ferroptosis. Did the authors consider whether the observed protective effect against ferroptosis in the FTH1-depleted mouse model be attributed not to the loss of NCOA4, but to a lack of proper interaction between NCOA4 and FTH1?

Please see Response C4 to Reviewer 1 for a possible hypothesis surrounding our observations. We did not explore whether there may be stereochemical effects by the loss of FTH1 on NCOA4-FTH1 interactions. However as demonstrated in **Supplementary Figure 12A** the appearance of the doublet band at the same MW for NCOA4 which only present in the *Fth1$^{\Delta LysM}$* samples is intriguing and may be suggestive of post-translational modifications, dimerization or cleavage

suggesting that NCOA4 may be localized to another membrane or has been altered which may affect its interaction with FTH1 or be ubiquitinated for degradation. An alternative hypothesis is that NCOA4 may also increase, independent to its role in iron metabolism; please see response C4 to Reviewer 1. We have added extra discussion points to the Revised MS (See Page 25).

C23. Paragraph "9" Loss of macrophage FTH1 results in lower extracellular iron and increased FTL ex-ferritin which is protective against hyperoxia
General: The ferritin levels observed by the authors are extremely high and approach those typically seen in certain macrophage activation syndromes. Did the authors also investigate what happens to other indicators of macrophage activation at a systemic level (cytopaenias, transaminase elevation, coagulopathy, increased soluble IL-2 receptor (CD25), soluble CD163, IL-18, etc.)? This would be essential to improve the quality of the manuscript.

While mice with a loss of FTH1 in macrophages/ monocytes have a compensatory rise in FTL, there is no evidence indicating that the loss of FTH1 leads to macrophage activation syndrome. There is no evidence of cytopenia/pancytopenia at baseline, and others have shown that there is no liver dysfunction or cytopenia in mice with global loss of FTH1 at baseline (*Hepatology. 2009 Sep;50(3):852-60*) with leukocyte composition in the liver and the spleen unaltered in *Fth1$^{\Delta LysM}$* mice (*JCI Insight. 2021 Jul 8;6(13):e141760*). In our study, there is a decrease in plasma leukocyte count in our hyperoxia model (**new panel Supplementary Figure 13A**), however this is not accompanied by anemia or histological evidence of hemophagocytosis in *Fth1$^{fl/fl}$* or *Fth1$^{\Delta LysM}$* (**new panel Supplementary Figure 13B-C**). Additionally, we have now included plasma cytokine data, showing that plasma cytokines linked to hemophagocytic lymphohistiocytosis (*Cytokine. 2016 Sep;85:14-7*) including IL-6, IL-10 and IFN-γ were also not different between the *Fth1$^{fl/fl}$* and *Fth1$^{\Delta LysM}$* mice at baseline or in response to hyperoxia (**new panel Supplementary Figure 13D**). We have added this new data and extra discussion points to the Revised MS (See Pages 18 and 23).

C24. As mentioned earlier, the increased expression of Ftl in the bone marrow-derived macrophages (BMDMs) of FTH1-depleted mice appears to be controversial, considering these macrophages are iron-depleted. Does the increased expression of Ftl sufficiently account for the significant rise in ferritin levels? Was hemophagocytosis present? Is there evidence of liver cytolysis or necrosis?

Please see Response to C18 and C23 to Reviewer 3 above to show there is no evidence of hemophagocytosis or liver dysfunction. It is unclear whether the increased FTL expression intracellularly, which is about only 25% increased by mRNA expression, sufficiently accounts for the significant rise in extracellular ferritin. As noted, FTH1 depletion is accompanied by a marked increase in NCOA4 protein levels, which as prior studies indicate is key to ferritin secretion. Therefore, it is not completely contradictory that the modest intracellular ferritin upregulation is accompanied by a marked increase in the flux of ferritin secretion, the extracellular levels of which presumably accumulates over time in the animal. As indicated in the response to C23, there was no evidence of erythrophagocytosis in the liver and spleen of mice in hyperoxia. We have added this new data and extra discussion points to the Revised MS (See Pages 18 and 23).

C25. Line 378: data concerning serum and BALF iron are reported at different time points (72 and 96 hours, respectively); could the author why they used different time points?

See Response to Reviewer 3 C14 above.

C26. Lines 397-398: the authors affirm that "The above data suggests that upon loss of FTH1 in macrophages, FTL is increased and secreted into the extracellular space". However, while the authors hypothesize that it is ferritin L, they have not quantified the presence of ferritin H or L in the extracellular space. I suggest that the authors revise the statement accordingly.

Reviewer 3 is correct in that we had not quantified the presence of light or heavy changes in the extracellular space. To address this comment, we performed unbiased proteomic profiling to quantify the amount of extracellular FTL present in the BALF of the $Fth1^{fl/fl}$ mice relative to $Fth1^{\Delta LysM}$ mice at baseline (**new panel Figure 6G**), which confirmed our *in vitro* findings. We have also added extra discussion points to the Revised MS (See Page 20).

C27. Lines 400-403: Authors hypothesize that in the context of elevated serum ex-ferritin in human ARDS, ex-ferritin, specifically FTL, not only represents a marker of cellular damage but a way to curb extracellular iron levels to protect from further ferroptosis. Unfortunately, this hypothesis appears to contradict existing literature, which has indicated that higher ferritin levels are an independent predictor of adverse outcomes in some ARDS models, including COVID-related ARDS? Could the authors comment on this aspect?

We are unsure how prior studies showing serum ferritin is an independent predictor of adverse events contradicts our hypothesis. In our opinion, this strengthens our hypothesis that extracellular ferritin plays an important role in ARDS pathobiology. We hypothesize that in response to acute lung injury such as hyperoxia, macrophages upregulate ferritin, an adaptive phenomenon which may also occur as monocytes differentiate into macrophages in the injured lung (*J Immunol (2024) 213 (10): 1508–1515*). Increased ferritin inside the cell circumvents from iron-associated toxicity and is host-pathogen response to chelate iron making it inaccessible for invading pathogens. This ferritin is also secreted into the extracellular space by macrophages where it can chelate iron and lead to the activation of several downstream events that initially may be helpful. But if the injury persists and not resolved this ferritin accumulation inside and outside then cell may promote ferroptosis and is linked to detrimental outcomes.

C28. Paragraph "10" Serum ex-ferritin consists predominantly of FTL in human ARDS. General: The observation that patients with deceased ARDS had higher L-ferritin levels than survivors contradicts the prior paragraph's hypothesis and requires the authors' clarification. Could the authors please clarify at what time point the dosage of the FTL and FTH1 was performed?

Reviewer 3 is correct in that we had hypothesised that individuals with higher FTL:FTH1 ratios would have better survival outcomes. However, our hypothesis was wrong as we observed that those with higher FTL did worse. Our only explanation for this is that the secretion of FTL into the extracellular space, along with lower FTH levels in those that survived is reflective of the adaptive response to injury where there is an attempt to resolve injury and inflammation. Please also see Response C18 to Reviewer 3, whereby others have also speculated that FTL increases as a compensatory mechanism to protect cells from ferroptosis and ROS (*Diabetologia (2020*) *63, 1588–1602, JCI Insight. 2021 Jul 8;6(13):e141760, Front Immunol. 2019 Feb 4:10:131).* Also, please See response to Reviewer 1 C6. We therefore hypothesize that FTL is a protective response of the cell to injury/inflammation and therefore is higher in individuals who have severe lung injury.

Point-By-Point Responses to the Reviewers of Manuscript NCOMMS-24-66679

We appreciate the constructive and insightful comments from the final remaining Reviewers and the editors. We have performed additional experiments leading to the generation of 6 new figure panels. A point-to-point response to each of the comments is enclosed below:

Reviewer 4:

Zhang et al. explored the protective role of macrophage derived ferritin in ARDS. They start with presenting the enrichment of ferritin in individuals with ARDS and HALI mice model, compared to controls. This finding aligns with the strong association between ferritin level and severity of covid-19 induced ARDS reported by others. Using several genetic modified mice models, they demonstrated that FTH in macrophages is detrimental while secreted FTL is protective in HALI. The study brings new insights into the iron immunity field. However, some parts of their work seemed contradictory to current understanding of iron induced ferroptosis, and conclusions are not firmly supported by their data. I will focus on the concerns previously raised by the reviewer 3.

General comments:

The authors have properly addressed C1-C5, C7; although they didn't provide the data of ferritin bound iron for C6, the current version is sufficient to support that ferritin itself is more relevant in their context.

Study Specific Comments

The authors have properly addressed C8-C12, C14-C28.

C1: For C13, the authors provided data from BMDM to further support that hyperoxia induces FTH and FTL in macrophages. However, the blot in Figure 2L requires quantification due to uneven sample loading.

A: We have quantified the Western Blot in **Figure 2L** and have included that panel as **new panel (Figure 2M)**. This analysis supports the notion that macrophage FTH1 is induced by *in vivo* hyperoxia exposure.

One extra comment:

C2: The methodology for assessing ferroptosis in FTH KO BMDM and FTH LysMKO mice is deemed too simplistic. If FTHKO macrophages are more sensitive to ferroptosis, the protective phenotype they observed in HO FTHKO mice could be attributed to a loss of pathogenic macrophages. A more integrative experimental design could help clarify these inconsistencies and improve the power of their findings. Therefore, it is strongly recommended that:

1. Flox and FTHKO BMDM can be exposed to FAC, RSL-3 in HO/RA conditions for 4, 12 and 24hours. Cell death should be determined using ANNEXIN V/Sytox staining in a flow cytometer;
2. Lipid peroxidation in BMDM treated as above could be determined using C11-BODIPY staining, and determined in a flow cytometer;
3. The labile iron pool in the macrophages from FTH LysMKO mice and control mice exposed to HO/RA should be illuminated using ferroorange staining in BALF as well as single-cell suspension from digested lung, and then determined in a flow cytometer;
4. Lipid peroxidation in the macrophages from FTH LysMKO mice exposed to HO/RA could be

determined using C11-BODIPY staining in BALF as well as single-cell suspension from digested lung, and then determined in a flow cytometer.

The authors cannot make their conclusion that FTHKO protects ferroptosis in macrophages unless they are supported by the data generated from the above experiments.

A: We agree with the Reviewer that more evidence linking ferroptosis to our observed phenotype would greatly strengthen our manuscript. Due to time constraints of the Revision timeline, we prioritized conducting experiments using our *in vivo* ARDS model to generate the most informative data possible. We exposed *Fth1*$^{fl/fl}$ and *Fth1*$^{\Delta LysM}$ mice to hyperoxia for 72 hours, to mirror the timepoint we used for 4-HNE staining in our original manuscript (**Figure 4O**) and then performed bronchoalveolar lavage (BAL) followed by lung digestion to isolate single cells. We performed immunophenotyping on the immune cells but also quantified their levels of Fe^{2+} and lipid peroxides. Using FerroOrange, we found that AMs from *Fth1*$^{\Delta LysM}$ mice have increased Fe^{2+} at baseline (**new panel Supplementary Figure 9A**), consistent with our BMDM results. Additionally, we found that CD11c$^+$SiglecF$^+$ AMs have increased Fe^{2+} following hyperoxia exposure (**new panel Figure 4P**). Critically, both CD11c$^+$SiglecF$^+$ AMs as well as SiglecF^{Hi} macrophages (which include monocyte-derived AMs or monoAMs, via the gating strategy illustrated in **new panel Supplementary Figure 9B**) have increased lipid peroxides as quantified by Liperfluo (**new panels Figure 4Q-R**), with macrophages from *Fth1*$^{\Delta LysM}$ mice having lower levels compared to those from *Fth1*$^{fl/fl}$ mice, consistent with the decreased 4-HNE staining on cytospins. We chose Liperfluo (Dojindo Molecular Technologies, Gaithersburg, MD) instead of BODIPY C11 because Liperfluo is a direct marker of lipid peroxides, whereas BODIPY C11 is more a measure of lipid moieties that may or may not be oxidized in ferroptosis. We believe that these new data support our prior findings that ferroptosis occurs in this murine HALI model and that FTH1 depletion protects against ferroptosis, at least in AMs and other lung macrophages.

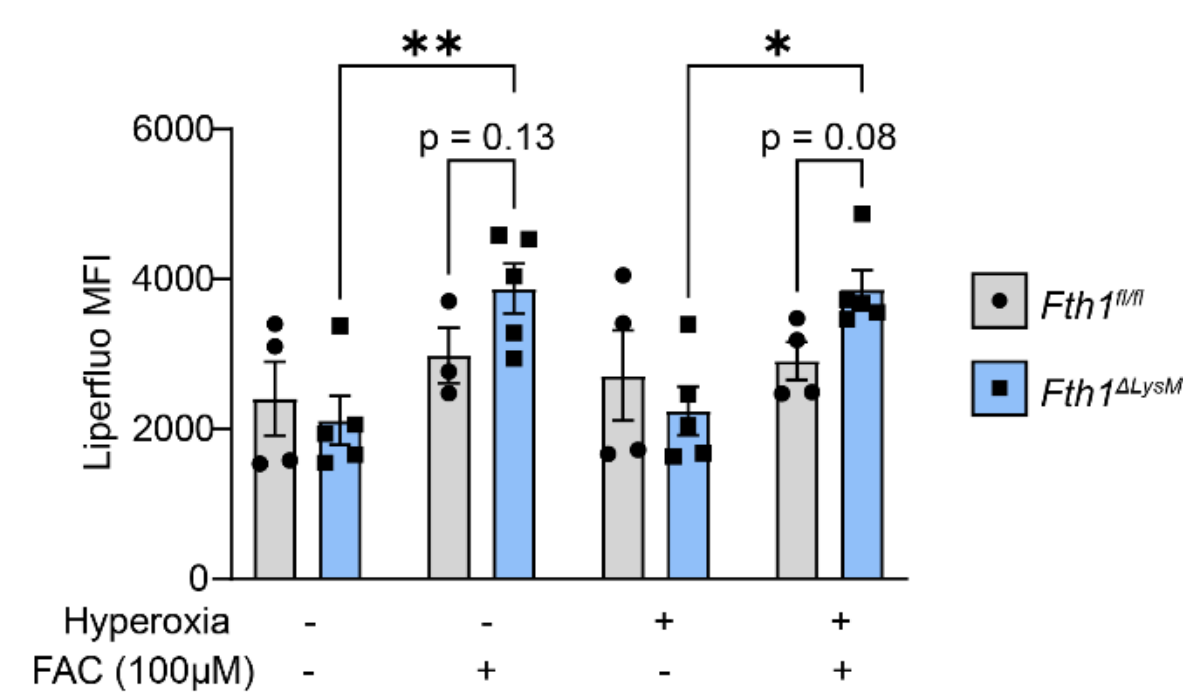

Although we utilized some data from our *in vitro* hyperoxia model in our original manuscript, we kept these data to a minimum because we believed that this model ultimately does not adequately represent what is occurring biologically in the lung in the *in vivo* model, much less human ARDS. The interplay of immune cells, both resident and infiltrating, with the local lung environment and the availability of iron and lipids (in the form of surfactant) cannot be simply replicated with oxygen exposure alone. Furthermore, attempting the experiments recommended by the Reviewer using multiple timepoints necessitates opening and closing the hyperoxia chamber multiple times, which causes large fluxes in oxygen concentration and is something we actively try to minimize in the *in vivo* hyperoxia model. Using Liperfluo, we found that the addition of 100μM ferric ammonium citrate (FAC) for 24 hours increased production of lipid peroxides for both *Fth1*$^{fl/fl}$ and *Fth1*$^{\Delta LysM}$ BMDMs, with *Fth1*$^{\Delta LysM}$ BMDMs being more sensitive to iron administration as these cells cannot store excess iron. This effect was not further boosted by hyperoxia exposure, but even after 24 hours of exposure of >90% oxygen there was little induction of cell death. We decided to not include this data in the main manuscript as we are uncertain if ferroptosis in this system is meaningful biologically with respect to the overall organism, a concern which has been raised by leaders in the field (Mishima E et al. Nat Rev Mol Cell Biol. 2025 Aug;26(8):615-630).

Reviewer #5:

C1. In the revised manuscript, the authors have appropriately addressed and responded to the comments raised by Reviewer #1. However, the reviewer has one remaining request. Regarding C8: "Specific ferroptosis inhibitors in vivo will help support the conclusion", the authors tested both Lip-1 and Fer-1 in the HALI animal model. Unfortunately, only the data for Lip-1 have been included in the manuscript, while the results for Fer-1 are mentioned only as “not shown.” Given that there are very few reports directly comparing the efficacy of Lip-1 and Fer-1 in the same animal model, even if the Fer-1 data were negative, presenting these results (at least as supplemental data) would be of value. It is well known that Fer-1 has much lower in vivo-stability compared with Lip-1, so limited efficacy of Fer-1 would be considered a reasonable outcome.

A: We have now included this data as **new panels Supplementary Figure 10C-D**.

Point-By-Point Responses to the Reviewers of Manuscript NCOMMS-24-66679

We appreciate the new comments from the Reviewer. We have performed additional analyses and provide a point-to-point response to each of the comments below:

Reviewer 4:

The authors have put some effort into responding to the raised concerns. However, they seemed to avoid the main question: whether there is more cell death in macrophages from FTH LysMKO mice. Meanwhile, the quality of the provided data needs improvement.

We apologize for this oversight and assure the Reviewer that this was not intentional avoidance. We include live/dead staining in all flow cytometry experiments, and we are pleased to share the results as the response to C2 below.

C1. The new Figure 2M indicates that only two wells of each group were included in the experiment. Moreover, the statistics did not reach significance, which compromises the conclusion.

We had N=3 replicates for these protein lysates (matching the Ns from the RNA-Seq experiment in Figure 2J-K) and chose to use two of the lysates because some of the protein concentrations were low and we intended this panel as a proof of principle to support our RNA-Seq data. We have added another replicate for quantification in **panel Figure 2L.**

C2. No measurement of cell death in the newly provided data was performed, which contradicts the author's claim that little death was induced and their observation in Figure 4M. It is important to have the cell death analysis via Sytox staining to support their hypothesis.

We include a live/dead stain in all flow cytometry experiments and strongly believe that this is essential for data quality and reproducibility. We apologize for not including this in the last revision – we had been so focused on the ferroptosis-specific readouts that this was an oversight. In the newly presented data from the last resubmission, we used Zombie NIR (Biolegend Cat# 423105), an amine-based live/dead fixable dye. Although this is not a nucleic acid-based dye like the Sytox family dyes the Reviewer referred to, functionally it has been well-validated as a death marker. As normal gating strategy excludes dead cells early in analysis, we reversed this workflow to gate for singlets and $CD11c^+$ $SiglecF^+$ AMs and then assessing for % live cells using Zombie NIR (gating strategy in **Supplementary Figure 9A**). As shown in **new panel Figure 4N,** there is significantly increased dead cells in BALF $CD11c^+$ $SiglecF^+$ AMs in $Fth1^{fl/fl}$ mice exposed to hyperoxia compared to room air mice, and this cell death is attenuated in $Fth1^{\Delta LysM}$ mice. These results complement the BALF lipid peroxides (**Figure 4R-S**) and FerroOrange (**Figure 4Q**) data from the last revision, as well as the BALF cell 4-HNE (**Figure 4P**) and TUNEL (**Figure 4M**) staining data to show that FTH1 deficient macrophages have lower cell death and possibly lower ferroptotic cell death in response to hyperoxia. To complete the dataset, we have added a **new panel Supplementary Figure 9C**, such that the Reviewer can see FerroOrange and Liperfluo evaluated in both BALF and lung $CD11c^+$ $SiglecF^+$ AMs.

Minor Comments

C3. The gating strategy in the panel Supplementary did not fully indicate the gating of CD11c+SiglecF+ AMs and SiglecFHi macrophages.

We have now included a more detailed gating strategy in **Supplementary Figure 9A**.

C4. Strangely, the authors use LysM-Cre to study AM, which is not an ideal Cre for gene deletion in AM (https://doi.org/10.1038/s41423-022-00936-4). The KO efficiency in AM from FTH LysMKO mice is unexpectedly high (Supplementary Figure 5C). Please explain.

We agree with the Reviewer that LysM-Cre is not an ideal system for targeting resident alveolar macrophages (AMs), which is why we incorporated an alternative Cd11c-Cre approach (**Figure 3H**). The knockdown efficiency for FTH1 in the BAL cells isolated from the LysMKo also surprised us, but we have validated this knockdown in FTH1 RNA levels by also assessing FTH1 protein expression, which we have added as a **new panel Supplementary Figure 5D**. Our only explanation for this is that these BAL cells may not be only resident macrophages but may also contain other myeloid cells. We would need to perform lineage tracing experiments in $Fth1^{\Delta LysM}$ mice, it is possible the AMs in $Fth1^{\Delta LysM}$ mice is a mixed population of resident AMs and infiltrating bone marrow-derived cells, which could potentially skew this KO efficiency. That said, we chose to proceed with the LysM Cre as hyperoxia induces the infiltration of monocytes and other bone marrow-derived myeloid cells and we wanted to ensure we also targeted ferritin in these cells too as they infiltrate the lung (Please see Response to Comment 13 from Reviewer 3 for the first revision for more detail on this).

Point-By-Point Responses to the Reviewers of Manuscript NCOMMS-24-66679

We appreciate the new comments from the Reviewer and agree that data quality is of utmost importance. We have performed additional experiments and analyses and provide a point-to-point response to each of the comments below:

Reviewer 4:

C1. The new Figure 4N should show the percentage of NIR+ AM and other myeloid cells (such as neutrophils and monocytes) in the gate of CD45 (the gating strategy in Supplementary Figure 9 for NIR should include CD45). The absolute number should also be plotted.

We apologize for the oversight in our gating strategy and present the data again using this gating strategy (**new panel Supplemental Figure 9A**) as recommended by the Reviewer: P1 > singlets > $CD45^+$ > $CD11c^+$ $CD11b^-$ > $CD11c^+$ Siglec-F^+ > Zombie NIR^- AMs. Since these data follow our initial observations of cytospin cell count differences, we investigated primarily the live/dead counts and percentages in BALF cells. In our last revision, we gated Zombie NIR^- live AMs from the $CD11c^+$ Siglec-F^+ population, which also exhibit $CD45^+$ expression. Therefore, it is not surprising that the new data after including the $CD45^+$ population in the gating strategy for AMs are consistent with our previous results. We observed significant differences in BALF Live AM % out of the parent $CD11c^+$ Siglec-F^+ AM population, which we gated from $CD45^+$ cells, between the $Fth1^{fl/fl}$ and $Fth1^{\Delta LysM}$ mice exposed to hyperoxia. We also include a new panel (**Figure 4O**) with counts of BALF Live AMs which mirror the differences in cell counts from our cytospins. Although there was a trend for increased $Ly6G^+$ neutrophil counts in $Fth1^{\Delta LysM}$ mice exposed to hyperoxia compared to control mice, there were no differences in the percentages of live neutrophils, unlike what was observed for AMs (**Supplementary Figure 9B-C**). It is clear from our gating strategy that there are classical, intermediate, and nonclassical monocytes in our $CD11b^+$ myeloid population. Since we are uncertain whether these monocyte subpopulations exhibit similar functions in regulating iron and ferroptosis in our model, and our primary data is focused on AMs, we decided not to include monocyte data so as not mislead the reader.

C2. Please provide the FMO control (cells stained with all the antibodies/dyes but NOT FerroOrange) for the FerroOrange staining (make a histogram plot merged with FMO and samples). This is because CD11c-PE-CY7 may interfere with the FerroOrange Channel.

The Reviewer is correct to point out this gap in our data. We have performed an independent experiment and can demonstrate that the FerroOrange signal we observed using our initial antibody panel with CD11c-PE-Cy7 was not an artifact (**Response Figure 1**), and the signal of FerroOrange dye in AMs detected via flow cytometry was higher in fully stained samples than in an FMO control for FerroOrange.

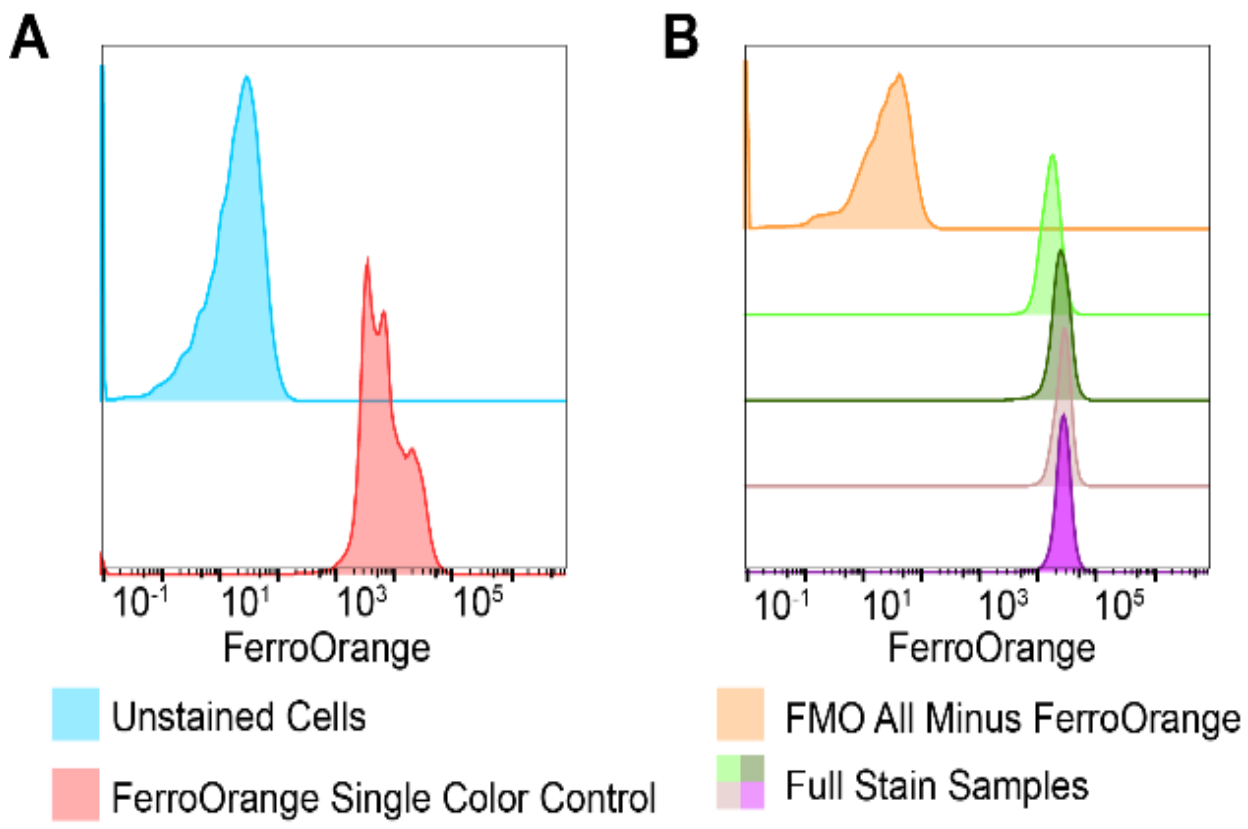

Response Figure 1. AM FerroOrange detection using flow cytometry. (A) FerroOrange dye signal in an unstained and single color control for FerroOrange in lung single cell suspensions from control mice and in a Fluorescence-Minus-One (FMO) for FerroOrange and full-stained samples (B).

To re-assure the Reviewer 4 the rigor of our work, we have also performed additional experiment with a new panel by replacing CD11c-PE-Cy7 with CD11c-BUV395. This resolved Reviewer 4's concern of interference of CD11c-PE-Cy7 with the FerroOrange PE channel altogether. Despite having fewer biological replicates, we observed a similar increase in FerroOrange fluorescence in lung CD11c+Siglec+ AMs in both *Fth1*$^{fl/fl}$ and *Fth1*$^{\Delta LysM}$ mice after hyperoxia exposure (**new panel Figure 4R**); the FMO for FerroOrange for this new panel is also shown in **Supplementary Figure 16A.** In addition, because Liperfluo emission (~535nm) could be influenced by green autofluorescence in the lungs, we also validated our findings on Liperfluo in AMs (with this new antibody panel) using an FMO control for the Liperfluo dye (**Supplementary Figure 16B**). We saw a similar observation whereupon AMs from *Fth1*$^{\Delta LysM}$ mice exposed to hyperoxia had markedly reduced Liperfluo fluorescence (**new panel Figure 4S**), which supports our extensive additional biochemical findings that *Fth1* depletion in macrophages protects these cells from ferroptosis.

We thank the Reviewer 4 and the editor for their careful consideration and constructive feedback throughout the review process. We hope that the extensive new data and revisions included in this fourth round fully resolve the remaining questions and demonstrate the rigor and completeness of the manuscript. We believe the study is now substantially strengthened and ready for final evaluation.
