# Supplementary material for: Targeting macrophage ferritin heavy chain mitigates ferroptosis and lung injury in experimental acute respiratory distress syndrome
Source: Nat Commun. 2026 Jun 27;17:8057. doi: 10.1038/s41467-026-74828-w (PMC13454589; doi:10.1038/s41467-026-74828-w)
Supplement: Supplementary file 4 [file 41467_2026_74828_MOESM4_ESM.pdf]

2B Hyperoxia BALF Protein

| RA | 72hr | 96hr |
|---|---|---|
| 1.150 | 0.818 | 2.953 |
| 0.287 | 0.591 | 3.503 |
| 0.500 | 1.191 | 2.498 |
| 0.447 | 0.711 | 4.177 |
| 0.586 | 0.536 | 2.297 |
| 0.467 | 1.563 | 2.584 |
| 0.782 | 0.771 | 3.083 |
| 1.549 | 1.429 | 1.287 |
| 1.834 | 1.735 | 1.633 |
| | 1.333 | 3.720 |
| | 0.813 | 3.431 |
| | 3.222 | 2.368 |
| | 1.892 | |
| | 2.006 | |
| | 1.677 | |

Male

Female

2C Hyperoxia Serum Ferritin

| Room Air | 72hr | 96hr |
|---|---|---|
| 966.222 | 1759.788 | 2378.845 |
| 868.775 | 1315.647 | 2553.104 |
| 953.453 | 2266.603 | 2286.521 |
| 1561.124 | 1542.527 | 2224.581 |
| | 1538.822 | |
| | 2065.170 | |

Male

Female

2D Hyperoxia BALF Ferritin

| RA | 72hr | 96hr |
|---|---|---|
| 235.486 | 162.810 | 380.763 |
| 197.660 | 280.180 | 565.953 |
| 143.037 | 331.566 | 228.296 |
| 213.885 | 503.529 | 545.286 |
| 259.273 | 325.613 | 234.440 |
| 295.585 | 297.499 | 295.392 |
| | | 356.987 |
| | | 321.936 |
| | | 1462.460 |
| | | 371.702 |
| | | 451.052 |
| | | 395.035 |
| | | 538.054 |
| | | 402.227 |

Male

Female

2E VILI BALF Ferritin

| No Vent | Low VT | High VT |
|---|---|---|
| 260.847 | 197.173 | 302.767 |
| 371.352 | 331.474 | 318.363 |
| 254.794 | 321.080 | 539.068 |
| 321.129 | 340.625 | 602.963 |
| | 327.344 | 474.023 |
| | | 429.350 |
| | | 413.247 |
| | | 480.449 |
| | | 471.779 |
| | | 773.157 |

From prior study
Sex not available

2F Flu BALF Ferritin

| PBS | D5 | D7 |
|---|---|---|
| 192.378 | 405.150 | 966.649 |
| 184.211 | 481.790 | 652.963 |
| 207.562 | 630.367 | 515.493 |
| 185.814 | 401.490 | 462.275 |
| | | 492.119 |
| | | 494.910 |
| | | 615.627 |
| | | 342.837 |
| | | 565.906 |
| | | 476.147 |
| | | 674.092 |
| | | 727.309 |
| | | 715.909 |
| | | 484.956 |

| | |
|---|---|
| Male | |
| Female | |

2G Sorted AM Fth1 qPCR

| RA | HO (96hr) |
|---|---|
| 0.967981 | 4.148232 |
| 1.00853 | 4.227919 |
| 1.027765 | |

2H Sorted AM Ftl qPCR

| RA | HO (96hr) |
|---|---|
| 1.015736 | 2.479585 |
| 0.786522 | 3.427224 |
| 1.254735 | |

2J Fth1 FPKM

| RA | 72hr | 96hr |
|---|---|---|
| 14657.33 | 15718.61 | 7718.37 |
| 11119.92 | 16254.33 | 14141.15 |
| 12140.60 | 14827.56 | 15315.41 |

Fold Change

| RA | 72hr | 96hr |
|---|---|---|
| 1.159665 | 1.243632 | 0.610665 |
| 0.87979 | 1.286017 | 1.118825 |
| 0.960545 | 1.173133 | 1.211731 |

2K Ftl FPKM

| RA | 72hr | 96hr |
|---|---|---|
| 7649.38 | 7589.90 | 7340.63 |
| 6308.01 | 7732.32 | 6759.26 |
| 6758.60 | 7220.29 | 7041.26 |

Fold Change

| RA | 72hr | 96hr |
|---|---|---|
| 1.10775 | 1.099137 | 1.063039 |
| 0.913499 | 1.119762 | 0.978847 |
| 0.978751 | 1.045611 | 1.019685 |

2L WB Quantification

| Lanes | ACTIN | FTH1 | FTH1/ACTIN | Normalized |
|---|---|---|---|---|
| 1 | 46329.17 | 38837.24 | 0.83828916 | 1.03147843 |
| 2 | 36066.23 | 28388.58 | 0.78712371 | 0.96852157 |
| 3 | 58234.44 | 63441.78 | 1.08942019 | 1.34048427 |
| 4 | 53347.03 | 52810.9 | 0.98995014 | 1.21809069 |
| 5 | 47542.2 | 37539.76 | 0.78960915 | 0.97157979 |
| 6 | 60311.34 | 59098 | 0.97988201 | 1.20570229 |
| RA Avg | 0.812706 | | | |

New Replicate

| | FTH1 | BACTIN | FTH1/BACTIN | |
|---|---|---|---|---|
| 1 | 54110.39 | 53952.76 | 1.00292174 | 1 |
| 2 | 47058.1 | 45399.69 | 1.0365292 | 1.03350955 |
| 3 | 40875.51 | 20542.69 | 1.98978357 | 1.98398687 |

3A LysM Fth1 Survival

| Hours | *Fth1* $^{fl/fl}$ | *Fth1* $^{\Delta LysM}$ |
|---|---|---|
| 90 | 1 | |
| 90 | 1 | |
| 111 | 1 | 1 |
| 111 | | 1 |
| 112 | | 1 |
| 116 | 1 | |
| 118 | | 1 |
| 124 | | 1 |
| 134 | 1 | 1 |
| 143 | | 1 |
| | | |
| 86 | 1 | |
| 86 | 1 | |
| 86 | 1 | |
| 86 | 1 | |
| 87 | 1 | |
| 90 | | 1 |
| 92 | | 1 |
| 98 | 1 | |
| 110 | 1 | |
| 110 | 1 | |
| 110 | | 1 |
| 111.5 | | 1 |
| 117.5 | | 1 |
| 136 | | 1 |
| 136 | | 1 |
| | | |
| 94.5 | 1 | |
| 97.5 | | 1 |
| 99 | 1 | |
| 99 | 1 | |
| 99.5 | 1 | 1 |
| 104 | 1 | |
| 118 | | 1 |
| 118.5 | | 1 |
| 121 | 1 | |
| 128 | | 1 |
| 128 | | 1 |
| 128.5 | | 1 |
| 140 | | 1 |
| | | |
| 80.5 | 1 | |
| 82.5 | 1 | |
| 92.5 | 1 | |
| 92.5 | 1 | |
| 95.5 | 1 | |
| 100.5 | 1 | |
| 100.5 | 1 | |
| 101.5 | 1 | |
| | | |
| 94 | 1 | 1 |
| 94 | 1 | 1 |
| 96.5 | 1 | |

3B LysM Fth1 BALF Protein

| Fth1fl/fl | | Fth1ΔLysM | |
|---|---|---|---|
| RA | HO (96hr) | RA | HO (96hr) |
| 1.150 | 2.953 | 0.466 | 1.569 |
| 0.287 | 3.503 | 0.361 | 1.250 |
| 0.500 | 2.498 | 0.293 | 1.135 |
| 0.447 | 4.177 | 0.214 | 2.497 |
| 0.586 | 2.297 | 0.044 | 1.310 |
| 0.467 | 2.584 | 0.305 | 2.641 |
| 0.782 | 3.083 | 0.871 | 2.676 |
| 1.549 | 1.287 | 0.849 | 1.822 |
| 1.834 | 1.633 | 1.488 | 0.945 |
| 1.476 | 3.720 | 2.088 | 1.120 |
| 0.776 | 3.431 | 0.617 | 1.573 |
| 1.082 | 2.368 | 1.675 | |
| 1.493 | | 1.435 | |

| |
|---|
| Male |
| Female |

3C LysM Fth1 BALF IgM

| Fth1fl/fl | | Fth1ΔLysM | |
|---|---|---|---|
| RA | HO (96hr) | RA | HO (96hr) |
| 745.51 | 2489.855 | 1313.36 | 1155.264 |
| 962.61 | 3146.548 | 362.94 | 1106.809 |
| 992.57 | 10997.43 | 1179.78 | 1073.725 |
| 1777.46 | 4643.699 | 907.66 | 2143.457 |
| 986.19 | 4499.004 | 135.04 | 1684.604 |
| 187.79 | 1443.52 | 903.87 | 4159.563 |
| 236.98 | 786.119 | 1115.18 | 1885.005 |
| 392.95 | 1355.713 | 1282.818 | 1274.666 |
| 505.73 | 3601.147 | 989.824 | 500.9764 |
| 1065.61 | 3751.551 | 1167.2 | 982.296 |
| 331.34 | 1556.389 | | 1030.17 |
| 548.77 | | | |
| 901.75 | | | |
| 343.57 | | | |
| 1801.43 | | | |
| 1223.143 | | | |
| 753.3399 | | | |
| 961.1845 | | | |
| 994.1455 | | | |
| 1211.177 | | | |
| 996.9584 | | | |

Male

Female

3D LysM Fth1 BALF LDH

| Fth1fl/fl | | Fth1ΔLysM | |
|---|---|---|---|
| RA | HO (96hr) | RA | HO (96hr) |
| 0.202 | 1.231 | 0.259 | 0.443 |
| 0.200 | 1.176 | 0.247 | 0.505 |
| 0.225 | 1.091 | 0.319 | 0.551 |
| 0.282 | 2.399 | | 1.246 |
| 0.152 | 1.502 | | 0.562 |
| 0.270 | 1.082 | | 0.930 |
| | 1.106 | | 0.832 |
| | 0.453 | | 0.896 |
| | 1.731 | | 1.065 |
| | 0.464 | | 0.908 |
| | 0.598 | | 0.396 |
| | 0.765 | | 0.421 |
| | 1.022 | | 0.673 |
| | 0.560 | | |

Male

Female

3E Histology Score

| Fth1fl/fl | | Fth1ΔLysM | |
|---|---|---|---|
| RA | HO (96hr) | RA | HO (96hr) |
| 6.2 | 30.5 | 2.8 | 55.5 |
| 1.4 | 77 | 4.2 | 24.5 |
| | 72 | 4.8 | 46.5 |
| | 77.2 | 1.4 | 41 |
| | 82 | | 26 |
| | 96 | | 55.3 |
| | 79 | | 25 |
| | 45.8 | | 54.1 |
| | 54.5 | | 79 |
| | 53.1 | | |

Male

Female

3F DAD Score

| Fth1fl/fl | | Fth1ΔLysM | |
|---|---|---|---|
| RA | HO (96hr) | RA | HO (96hr) |
| 0 | 2.5 | 0 | 2 |
| 0 | 3 | 0 | 1.5 |
| | 3 | 0 | 1.5 |
| | 3 | 0 | 2 |
| | 3.5 | | 1 |
| | 3.5 | | 2 |
| | 3 | | 1 |
| | 2 | | 2 |
| | 2 | | 2.5 |
| | 2 | | |

| |
|---|
| Male |
| Female |

3H Cd11c Fth1 Survival

| Hours | *Fth1* $^{fl/fl}$ | *Fth1* $^{\Delta Cd11c}$ |
|---|---|---|
| 86 | 1 | |
| 87.5 | 1 | 1 |
| 93.5 | | 1 |
| 94.5 | | |
| 94.5 | | |
| 96 | | 1 |
| 98 | 1 | |
| 110.5 | 1 | 1 |
| 110.5 | 1 | 1 |
| 110.5 | | 1 |
| | | |
| 85 | 1 | |
| 85.5 | | |
| 92 | 1 | |
| 93.5 | | 1 |
| 108 | 1 | 1 |
| 108 | 1 | |
| 108 | 1 | |
| 108 | 1 | |
| 108 | 1 | |
| 108 | 1 | 1 |
| 113.5 | | 1 |
| 117.5 | | 1 |
| 118 | 1 | |
| 132 | | 1 |
| | | |
| 85 | 1 | |
| 98 | 1 | 1 |
| 98 | 1 | |
| 98 | 1 | |
| 99.5 | 1 | |
| 101 | 1 | |
| 104 | 1 | 1 |
| 104 | | 1 |
| 105.5 | 1 | |
| 119 | | 1 |
| 119 | | 1 |

3I LysM Fth1 BALF Macs/Monos

| Fth1fl/fl | | | Fth1ΔLysM | | |
|---|---|---|---|---|---|
| RA | 72hr | 96hr | RA | 72hr | 96hr |
| 6.2 | 6.22 | 0.45 | 1.48 | 5.99 | 16.16 |
| 10.2 | 4.24 | 0.71 | 3.43 | 4.8 | 16.73 |
| 7.84 | 4.88 | 0.33 | 4.32 | 9.27 | 11.22 |
| 5.4 | 3.69 | 0.26 | | 6.48 | 7.19 |
| | 4.29 | 0.24 | | 3.71 | 16.4 |
| | 3.89 | 3.52 | | 4.2 | 3.91 |
| | 4.97 | 3.31 | | 3.58 | 9.69 |
| | 4.01 | | | 2.64 | 10.31 |
| | 5.12 | | | 5.32 | |
| | 5.1 | | | 7.52 | |
| | 5.08 | | | 3.68 | |
| | 6.02 | | | | |

Male

Female

3J LysM Fth1 BALF Neutrophils

| Fth1fl/fl | | | Fth1ΔLysM | | |
|---|---|---|---|---|---|
| RA | 72hr | 96hr | RA | 72hr | 96hr |
| 0 | 0 | 1 | 0.02 | 1.23 | 0.58 |
| 0.02 | 0.03 | 2.49 | 0.01 | 2.99 | 0.96 |
| 0 | 0 | 0.67 | 0.08 | 1.73 | 0.89 |
| 0.1 | 0.05 | 1.92 | | 2.41 | 4.66 |
| | 0.06 | 3.56 | | 4.39 | 3.65 |
| | 0.05 | 0.55 | | 3.35 | 3.15 |
| | 0.26 | 0.29 | | 4.34 | 1.48 |
| | 0.03 | | | 0.55 | 2.1 |
| | 0.27 | | | 0.64 | |
| | 0.06 | | | 0.32 | |
| | 0.24 | | | 0.64 | |

| |
|---|
| Male |
| Female |

3K LysM Fth1 BALF NM Ratio

| Fth1fl/fl | | | Fth1ΔLysM | | |
|---|---|---|---|---|---|
| RA | 72hr | 96hr | RA | 72hr | 96hr |
| 0 | 0 | 2.25 | 0.01 | 0.21 | 0.04 |
| 0 | 0.01 | 3.48 | 0 | 0.62 | 0.06 |
| 0 | 0.96 | 2.03 | 0.02 | 0.19 | 0.08 |
| 0.02 | 0 | 7.46 | | 0.37 | 0.65 |
| | 0.01 | 14.63 | | 1.18 | 0.22 |
| | 0.01 | 0.15 | | 0.8 | 0.81 |
| | 0.01 | 0.09 | | 1.21 | 0.15 |
| | 0.07 | | | 0.21 | 0.2 |
| | 0.01 | | | 0.12 | |
| | 0.05 | | | 0.04 | |
| | 0.01 | | | 0.17 | |
| | 0.04 | | | | |

| |
|---|
| Male |
| Female |

3N AM by Flow

| Fth1fl/fl | | Fth1ΔLysM | |
|---|---|---|---|
| RA | HO (96hr) | RA | HO (96hr) |
| 367 | 2 | 2655 | 1421 |
| 334 | 34 | 1373 | 438 |
| | 1 | | 1212 |
| | | | 738 |
| | | | 57 |
| | | | 5589 |

3O LysM Fth1 BALF CXCL1

| Fth1fl/fl | | | Fth1ΔLysM | | |
|---|---|---|---|---|---|
| RA | 72hr | 96hr | RA | 72hr | 96hr |
| 1.572 | 8.475 | 56.083 | 4.716 | 6.469 | 27.549 |
| 1.475 | 12.659 | 109.365 | 1.766 | 9.283 | 23.064 |
| 1.611 | 14.046 | 45.568 | 2.453 | 14.778 | 14.523 |
| 2.017 | 11.011 | 17.050 | 2.919 | 15.526 | 19.637 |
| | 13.538 | 64.150 | 4.643 | 13.208 | 13.878 |
| | 16.803 | 26.180 | | 11.313 | 29.047 |
| | 15.874 | 71.588 | | | 48.039 |
| | 16.908 | 42.513 | | | |
| | | 322.530 | | | |
| | | 21.278 | | | |

| |
|---|
| Male |
| Female |

3P LysM Fth1 BALF MCP-1

| Fth1fl/fl | | | Fth1ΔLysM | | |
|---|---|---|---|---|---|
| RA | 72hr | 96hr | RA | 72hr | 96hr |
| 13.131 | 22.620 | 354.904 | 18.433 | 87.604 | 177.531 |
| 0.000 | 29.165 | 1301.528 | 37.894 | 33.393 | 270.756 |
| 0.000 | 26.233 | 496.420 | 29.480 | 89.177 | 160.311 |
| | 179.158 | 523.124 | | 50.064 | 898.575 |
| | 32.468 | 321.599 | | 82.358 | 260.063 |
| | 33.168 | 469.150 | | 139.893 | 100.566 |
| | 53.640 | 409.756 | | 97.092 | 288.510 |
| | | 1076.746 | | | 311.785 |
| | | 226.095 | | | 468.712 |
| | | 447.746 | | | 343.400 |
| | | 611.339 | | | 231.861 |
| | | 525.766 | | | 102.791 |
| | | 165.210 | | | 328.676 |

| |
|---|
| Male |
| Female |

4C LysM Fth1 BMDM Iron

| *Fth1* $^{fl/fl}$ | *Fth1* $^{\Delta LysM}$ |
|---|---|
| 15.31404 | 1.043684 |
| 74.83419 | 1.357482 |
| 8.479542 | 36.22663 |
| 72.72321 | 1.496623 |
| 48.01964 | 4.335294 |

4D LysM Fth1 FerroOrange

| Fth1fl/fl | | | Fth1ΔLysM | | |
|---|---|---|---|---|---|
| CTRL | 30min | 4hr | CTRL | 30min | 4hr |
| 1235.4 | 2097.8 | 1631.7 | 2496.1 | 4965.3 | 4550.3 |
| 1957.5 | 2096.4 | 2112 | 3405.6 | 6182.3 | 5792.7 |
| 2660 | 4480.8 | 2983.7 | 4264.2 | 8629.4 | 9396.4 |
| 1471.3 | 1830.9 | 1891 | 2398.1 | 3911.3 | 4829.6 |

4E LysM Fth1 FAC Viability

| Fth1fl/fl | | | | Fth1ΔLysM | | | |
|---|---|---|---|---|---|---|---|
| FAC 0 | FAC 50 | FAC 100 | FAC 200 | FAC 0 | FAC 50 | FAC 100 | FAC 200 |
| 100 | 83.78679 | 82.68492 | 85.38147 | 100 | 76.29799 | 68.88229 | 38.89782 |
| 100 | 107.5693 | 112.602 | 116.3739 | 100 | 79.28181 | 39.39079 | 23.91873 |
| 100 | 102.2442 | 102.5327 | | 100 | 101.2603 | 68.16061 | 37.7077 |
| 100 | 107.6651 | 111.7681 | 98.47472 | 100 | 112.1583 | 51.06652 | |

4H LysM Fth1 RSL3 Viability

| *Fth1* $^{fl/fl}$ | *Fth1* $^{\Delta LysM}$ |
| --- | --- |
| 68.92274 | 90.07659 |
| 64.36548 | 81.99531 |
| | 87.10223 |

4I In Vitro Hyperoxia Fth1 qPCR

| CTRL | HO |
|---|---|
| 1.013649 | 2.40886 |
| 1.002469 | 1.918781 |
| 1.041021 | 1.680546 |

4J In Vitro Hyperoxia Ftl qPCR

| CTRL | HO |
|---|---|
| 1.014598 | 2.35528 |
| 1.001038 | 1.87741 |
| 1.04013 | 1.910066 |

4K LysM Fth1 In Vitro Hyperoxia Viability

| *Fth1* $^{fl/fl}$ | *Fth1* $^{\Delta LysM}$ |
|---|---|
| 72.5753 | 96.61916 |
| 84.95048 | 88.02115 |
| 90.48128 | 96.42023 |
| 92.59037 | 110.7593 |
| 67.98998 | 85.95942 |

4M LysM Fth1 Cytospin TUNEL

| Fth1fl/fl | | Fth1ΔLysM | |
|---|---|---|---|
| RA | HO (72hr) | RA | HO (72hr) |
| 6.521739 | 16.4557 | 8.187135 | 14.28571 |
| 7.142857 | 38.125 | 13.69863 | 22.38806 |
| 2.666667 | 34.66667 | 7.534247 | 42.98246 |
| 4.411765 | 13.93728 | 5.982906 | 22.05882 |
| | 29.62963 | 10.57269 | 17.83439 |
| | 18.21561 | | 10.12658 |
| | 20.38217 | | 35.38462 |
| | 34.21053 | | 25.5814 |
| | 36.58537 | | 8.707124 |
| | | | 5.116279 |
| | | | 0.699301 |
| | | | 11.18012 |
| | | | 5.789474 |
| | | | 18.5567 |

| |
|---|
| Male |
| Female |

4N BALF AM Zombie NIR Neg Percent

| Fth1fl/fl | | Fth1ΔLysM | |
|---|---|---|---|
| RA | HO (72hr) | RA | HO (72hr) |
| 92.8 | 86.5 | 88.8 | 91.1 |
| 95.9 | 82.5 | 91.9 | 90.6 |
| 90.3 | 93.5 | 84.7 | 92.2 |
| 94.1 | 85.1 | 89.8 | 96.1 |
| | 62.5 | 95.1 | 92.3 |
| | 73 | | 90.6 |
| | 67.4 | | |

| |
|---|
| Male |
| Female |

4O BALF AM Zombie NIR Neg Counts

| Fth1fl/fl | | Fth1ΔLysM | |
|---|---|---|---|
| RA | HO (72hr) | RA | HO (72hr) |
| 9260 | 340 | 5557.88 | 1601.333 |
| 9930 | 430 | 351.3333 | 1182.667 |
| 1836 | 3420 | 287.3333 | 2748 |
| 9620 | 1142 | 4648 | 2282.638 |
| | 474 | 700.0259 | 5469.054 |
| | 791.3333 | | 1972 |
| | 1216.667 | | |

| |
|---|
| Male |
| Female |

4Q LysM Fth1 4-HNE Quant

| Fth1fl/fl | | Fth1ΔLysM | |
|---|---|---|---|
| RA | HO (72hr) | RA | HO (72hr) |
| 0.556378 | 11.16716 | 1.7211 | 8.863127 |
| 0.890509 | 45.1647 | 1.108287 | 27.30749 |
| 2.10358 | 26.83807 | 5.552032 | 20.70968 |
| | 14.7704 | | 9.797613 |
| | 34.60355 | | 1.01468 |
| | 29.05747 | | 26.98889 |
| | | | 12.81067 |
| | | | 18.29308 |
| | | | 9.344347 |

4R Lung AM FerroOrange

| Fth1fl/fl | | Fth1ΔLysM | |
|---|---|---|---|
| RA | HO (72hr) | RA | HO (72hr) |
| 22568 | 31105 | 8166 | 21919 |
| 18154 | 29879 | 16223 | 25101 |
| 16903 | 30909 | 19114 | 32960 |
| | 28033 | | 29672 |
| | 25351 | | 11891 |

| |
|---|
| Male |
| Female |

4S Lung AM Liperfluo

| Fth1fl/fl | | Fth1ΔLysM | |
|---|---|---|---|
| RA | HO (72hr) | RA | HO (72hr) |
| 1719 | 1840 | 1991 | 1165 |
| 1699 | 1927 | 1841 | 1231 |
| 1789 | 2027 | 1810 | 1146 |
| | 2079 | | 999 |
| | 1755 | | 1760 |

| |
|---|
| Male |
| Female |

5B Lip-1 Hyperoxia BALF Protein

| DMSO | Lip-1 |
|---|---|
| 1.724 | 4.025 |
| 1.553 | 2.050 |
| 2.900 | 1.001 |
| 0.873 | 1.102 |
| 1.935 | 1.889 |
| 2.221 | 3.149 |
| 2.271 | 2.462 |

| |
|---|
| Male |
| Female |

5C Lip-1 Hyperoxia BALF LDH

| DMSO | Lip-1 |
|---|---|
| 0.987 | 0.636 |
| 0.8385 | 0.563 |
| 0.65 | 0.5535 |
| 0.4255 | 1.1225 |
| 0.4475 | 0.364 |
| 0.539 | 0.609 |
| 1.0895 | 1.06 |

| |
|---|
| Male |
| Female |

5D Lip-1 Hyperoxia BALF Cell Count

| DMSO | Lip-1 |
|---|---|
| 0.8 | 1 |
| 0.4 | 0.8 |
| 1.1 | 0.2 |
| 15.3 | 0.5 |
| 1.1 | 1.2 |
| 8.1 | 1.4 |
| 15.6 | 4.8 |

| | |
|---|---|
| 1.724 | 4.025 |
| 1.553 | 2.050 |
| 2.900 | 1.001 |
| 0.873 | 1.102 |
| 1.935 | 1.889 |
| 2.221 | 3.149 |
| 2.271 | 2.462 |

| |
|---|
| Male |
| Female |

5F DFP BALF LDH

| CTRL | | DFP | |
|---|---|---|---|
| RA | HO (96hr) | RA | HO (96hr) |
| 0.2325 | 1.087 | 0.197 | 2.451 |
| 0.2735 | 0.358 | 0.1795 | 1.956 |
| 0.251 | 0.316 | 0.2045 | 1.666 |
| | 0.893 | 0.2235 | 0.794 |
| | 0.752 | | 1.101 |
| | | | 0.815 |
| | | | 0.935 |
| | | | 1.371 |

5G DFP BALF Protein

| CTRL | | DFP | |
| --- | --- | --- | --- |
| RA | HO (96hr) | RA | HO (96hr) |
| 2.116 | 4.441 | 2.367 | 5.551 |
| 2.46 | 2.444 | 0.214 | 5.107 |
| 1.652 | 1.756 | 2.19 | 4.866 |
| | 4.401 | 1.046 | 3.307 |
| | 4.958 | | 4.462 |
| | | | 2.979 |
| | | | 3.937 |
| | | | 5.202 |

5H DFP BALF IgM

| CTRL | | DFP | |
|---|---|---|---|
| RA | HO (96hr) | RA | HO (96hr) |
| 988.741 | 3393.656 | 1792.907 | 4215.769 |
| 1033.295 | 2354.934 | 126.453 | 3930.315 |
| 607.209 | 1111.705 | 581.735 | 3485.747 |
| | 2079.815 | 360.361 | 2045.339 |
| | 3645.661 | | 14212.39 |
| | | | 4106.566 |
| | | | 6946.829 |
| | | | 6922.769 |

5I DFP BALF Macs/Monos

| CTRL | | DFP | |
|---|---|---|---|
| RA | HO (96hr) | RA | HO (96hr) |
| 1 | 13.59 | 1.06 | 4.21 |
| 0.88 | 12.4 | 1.96 | 5.51 |
| 2.66 | 16.12 | 1.8 | 2.17 |
| | 1.09 | 2.29 | 12.15 |
| | 4.58 | | 9.07 |
| | | | 0.1 |
| | | | 1.14 |
| | | | 4.42 |

5J DFP BALF Neutrophils

| CTRL | | DFP | |
|---|---|---|---|
| RA | HO (96hr) | RA | HO (96hr) |
| 0 | 0.69 | 0 | 2.1 |
| 0 | 0.17 | 0 | 0.42 |
| 0 | 0.9 | 0 | 4.33 |
| | 0.04 | 0 | 2.76 |
| | 4.58 | | 11.04 |
| | | | 7.96 |
| | | | 4.99 |
| | | | 5.44 |

5K DFP BALF NM Ratio

| CTRL | DFP |
|---|---|
| 0.05 | 0.5 |
| 0.01 | 0.08 |
| 0.06 | 2 |
| 0.03 | 0.23 |
| 1 | 1.22 |
| | 4.38 |
| | 1.23 |

5L Cd11c Ncoa4 RSL3 Viability

| *Ncoa4 fl/fl* | *Ncoa4 ΔCd11c* |
|---|---|
| 67.474 | 105.486 |
| 60.73394 | 97.525 |
| 57.75931 | 84.41863 |
| 74.48734 | 119.9858 |
| | 107.5496 |

5M Cd11c Ncoa4 BALF Protein

| Ncoa4fl/fl | | Ncoa4ΔCd11c | |
|---|---|---|---|
| RA | HO (96hr) | RA | HO (96hr) |
| 0.627 | 1.45 | 0.889 | 2.935 |
| 2.269 | 2.241 | 0.939 | 0.847 |
| 0.276 | 4.243 | 0.076 | 2.164 |
| | 2.452 | | 4.517 |
| | 2.318 | | 4.425 |
| | 5.228 | | 3.985 |
| | 3.74 | | 2.907 |

| |
|---|
| Male |
| Female |

5N Cd11c Ncoa4 BALF NM Ratio

| Ncoa4fl/fl | | Ncoa4ΔCd11c | |
|---|---|---|---|
| RA | HO (96hr) | RA | HO (96hr) |
| 0.00995 | 0.022026 | 0 | 0.03252 |
| 0 | 0.045752 | 0.005115 | 0.333333 |
| 0.003861 | 0.069767 | 0 | 1.05 |
| | 0.109375 | | 0.75 |
| | | | 0.061538 |

| |
|---|
| Male |
| Female |

6A LysM Fth1 BALF Iron

| Fth1fl/fl | | Fth1ΔLysM | |
|---|---|---|---|
| RA | HO (96hr) | RA | HO (96hr) |
| 164.4993 | 383.5823 | 243.4459 | 126.5259 |
| 164.0131 | 426.9791 | 98.19286 | 81.43198 |
| 140.4074 | 158.0359 | 151.0876 | 337.7595 |
| | 259.2322 | | 177.1818 |
| | 333.669 | | 196.3308 |
| | 294.9172 | | 163.7814 |
| | | | 141.5356 |

6B LysM Fth1 BALF Ferritin

| Fth1fl/fl | | Fth1ΔLysM | |
|---|---|---|---|
| RA | HO (96hr) | RA | HO (96hr) |
| 235.486 | 380.763 | 36060.69 | 8968324 |
| 197.660 | 565.953 | 20025.04 | 8711540 |
| 143.037 | 228.296 | 23656.6 | 9058572 |
| 213.885 | 545.286 | 35706.16 | 10781269 |
| 162.81 | 234.440 | 17754.61 | 8157533 |
| 280.18 | 295.392 | 34699.12 | |
| | 356.987 | | |
| | 321.936 | | |
| | 1462.460 | | |
| | 371.702 | | |
| | 451.052 | | |
| | 395.035 | | |
| | 538.054 | | |
| | 402.227 | | |

Male

Female

6C LysM Fth1 Serum Ferritin

| *Fth1* $^{fl/fl}$ | *Fth1* $^{\Delta LysM}$ |
|---|---|
| 912.2668 | 73525.24 |
| 1062.262 | 70052.08 |
| 882.0672 | 74653.4 |
| | 56010.52 |

6D Cd11c Fth1 BALF Ferritin

| *Fth1* $^{fl/f}$ | *Fth1* $^{\Delta Cd11c}$ |
|---|---|
| 637.6253 | 29870.21 |
| 724.2022 | 31382.91 |
| 559.8818 | 57767.62 |

6E Cd11c Ncoa4 BALF Ferritin

| Ncoa4fl/fl | | Ncoa4ΔCd11c | |
|---|---|---|---|
| RA | HO (96hr) | RA | HO (96hr) |
| 113.647 | 156.327 | 94.428 | 142.982 |
| 105.056 | 195.941 | 85.804 | 124.593 |
| 75.243 | 218.902 | 81.726 | 108.985 |
| | 180.502 | | 176.448 |
| | 187.04 | | 196.765 |
| | 258.237 | | 155.19 |
| | 150.707 | | 104.999 |

| |
|---|
| Male |
| Female |

6F LysM Fth1 Supernatant Ferritin Release

| Fth1fl/fl | | | | Fth1ΔLysM | | Fth1ΔLysM | |
|---|---|---|---|---|---|---|---|
| 0hr | 16hr | 24hr | 48hr | 0hr | 16hr | 24hr | 48hr |
| 95.195 | 106.878 | 104.790 | 114.899 | 265.946 | 716.675 | 915.961 | 1370.004 |
| 103.441 | 110.060 | 116.035 | 122.879 | 216.865 | 629.794 | 970.599 | 1275.218 |
| 104.716 | 95.188 | 125.768 | 147.674 | 319.705 | 1134.971 | 1490.417 | 1719.200 |
| 103.979 | 111.269 | 120.454 | 139.250 | 440.932 | 765.351 | 2299.090 | 4908.586 |

6J BALF Transplant Weight Loss

| *Fth1* $^{fl/fl}$ BALF | *Fth1* $^{\Delta LysM}$ BALF |
|---|---|
| 15.43 | 15.71 |
| 15.10 | 11.39 |
| 16.84 | 14.93 |
| 15.71 | 12.78 |
| 14.87 | 13.07 |
| 11.24 | 13.18 |
| 14.29 | 9.09 |
| 15.44 | 12.13 |
| 17.62 | 9.47 |
| 7.92 | 10.89 |
| 13.94 | 10.00 |
| 11.48 | 11.79 |

| |
|---|
| Male |
| Female |

6K BALF IgM

| *Fth1*$^{fl/fl}$ BALF | *Fth1*$^{\Delta LysM}$ BALF |
|---|---|
| 14181.770 | 15102.380 |
| 19400.610 | 6318.565 |
| 7174.002 | 8231.787 |
| 19981.370 | 17791.880 |
| 5731.205 | 6801.089 |
| 14360.070 | 18142.740 |
| 35961.560 | 23297.350 |
| 8376.500 | 9357.540 |
| 28602.110 | 10178.950 |
| 9644.023 | 2306.592 |
| 3254.136 | 3274.540 |
| 1362.021 | 2553.609 |
| 2712.073 | |

| |
|---|
| Male |
| Female |

6L Human Serum Ferritin in COVID

| Ferritin (ng/mL) | FTL | FTH1 |
|---|---|---|
| 486.9 | 205000000 | 58800000 |
| 729.6 | 266000000 | 110000000 |
| 903.2 | 207000000 | 72600000 |
| 1086.4 | 317000000 | 104000000 |
| 1559.8 | 440000000 | 102000000 |
| 3077.6 | 5250000000 | 655000000 |
| 378.9 | 71800000 | 6560000 |
| 508 | 72300000 | 37500000 |
| 1204.5 | 88100000 | 39900000 |
| 1335.4 | 123000000 | 90900000 |
| 1991.9 | 817000000 | 410000000 |
| 2554 | 557000000 | 82700000 |

6M Human Serum FTH1/FTL Ratio

| Dead | Alive |
|---|---|
| 8.01 | 2.85 |
| 10.94 | 3.05 |
| 2.21 | 4.31 |
| | 1.93 |
| | 1.99 |

S2C Human Monocyte Proteomics FTH1

| HC | ARDS |
| --- | --- |
| 9.688238 | 10.1221 |
| 13.08108 | 10.50907 |
| 8.154542 | 103.3215 |
| | 48.55952 |

S2D Human Monocyte Proteomics FTL

| HC | ARDS |
|---|---|
| 28.99777 | 127.1902 |
| 44.91515 | 74.67932 |
| 22.25504 | 278.0453 |
| | 268.4682 |

S3A S. pneumoniae BALF Ferritin

| PBS | Strep |
|---|---|
| 248.917 | 189.912 |
| 201.446 | 194.149 |
| | 205.399 |
| | 227.536 |
| | 482.618 |
| | 419.999 |

S3B BAL Cell Fth1 qPCR

| RA | HO (72hr) |
|---|---|
| 1.062095 | 11.66671 |
| 1.003126 | 2.373986 |
| 1.012837 | 9.589284 |
| | 8.164538 |
| | 4.029133 |
| | 13.40361 |

S3C BAL Cell Ftl qPCR

| RA | HO (72hr) |
| --- | --- |
| 1.200252 | 3.64135 |
| 1.063024 | 1.874262 |
| 1.010237 | 2.686423 |
| | 3.919912 |
| | 2.587963 |
| | 3.079872 |

S3D Fth1 BulkSeq TPM

| CTRL | LPS |
| --- | --- |
| 1522.26 | 5368.55 |
| 1841.38 | 6844.95 |
| 1236.65 | 7341.54 |
| | 7625.61 |
| | 7134.12 |
| | 6107.47 |
| | 4569.02 |

S5B LysM Fth1 BMDM Fth1 qPCR

| *Fth1* $^{fl/fl}$ | *Fth1* $^{\Delta LysM}$ |
|---|---|
| 1.000079 | 0.020255 |
| 1.001347 | 0.018804 |
| 1.000321 | 0.058831 |

S5C LysM Fth1 AM Fth1 qPCR

| *Fth1* $^{fl/fl}$ | *Fth1* $^{\Delta LysM}$ |
| --- | --- |
| 1.005061 | 0.018839 |
| 1.000811 | 0.08611 |

S5G LysM Fth1 BMDM LPS IL-6

| *Fth1 fl/fl* | | | *Fth1 ΔLysM* | | |
|---|---|---|---|---|---|
| Untreated | 4hr | 24hr | Untreated | 4hr | 24hr |
| 0 | 43.17755 | 415.0417 | 0 | 0 | 92.96013 |
| 0 | 16.60228 | 838.9556 | 0 | 14.88853 | 95.53727 |
| 0 | 68.9172 | 364.7379 | 0 | 11.4613 | 126.4796 |

S5H LysM Fth1 BMDM LPS IL-6

| *Fth1* $^{fl/fl}$ | | | *Fth1* $^{\Delta LysM}$ | | |
|---|---|---|---|---|---|
| Untreated | 4hr | 24hr | Untreated | 4hr | 24hr |
| 0 | 757.3988 | 296.5156 | 0 | 523.6516 | 211.6158 |
| 0 | 670.1435 | 465.5025 | 0 | 371.6404 | 234.3102 |
| 0 | 666.8065 | 334.7014 | 0 | 384.541 | 251.0216 |

S6A LysM Fth1 Influenza Weight Loss

| *Fth1* $^{fl/fl}$ | *Fth1* $^{\Delta LysM}$ |
|---|---|
| 18.8 | 12.5 |
| 22.8 | 19.0 |
| 21.8 | 25.3 |
| 19.6 | 15.3 |
| 20.7 | 21.9 |
| 20.5 | 12.4 |
| 25.3 | 21.0 |
| 27.5 | 20.5 |
| 19.2 | 29.1 |
| 26.6 | 19.0 |
| 25.3 | 22.9 |
| 28.1 | 19.4 |
| 24.2 | |
| 23.0 | |

Male

Female

S6B LysM Fth1 Influenza BALF Protein

| Fth1fl/fl | | Fth1ΔLysM | |
|---|---|---|---|
| PBS | Day 7 | PBS | Day 7 |
| 0.203 | 1.414 | 0.064 | 1.01 |
| 0.276 | 1.419 | 0.151 | 1.614 |
| 0.037 | 1.323 | | 1.543 |
| 0.123 | 1.46 | | 1.301 |
| 0.108 | 1.627 | | 1.202 |
| | 1.617 | | 1.103 |
| | 2.023 | | 1.44 |
| | 1.915 | | 1.66 |
| | 1.437 | | 1.552 |
| | 1.591 | | 1.827 |
| | 1.553 | | 1.66 |
| | 1.829 | | 1.135 |
| | 1.282 | | 1.662 |
| | 1.581 | | |

Male

Female

S7A Cd11c Fth1 BALF Cell Count

| | Fth1fl/fl | | | Fth1ΔCd11c | |
|---|---|---|---|---|---|
| RA | 72hr | 96hr | RA | 72hr | 96hr |
| 3.43 | 9 | 18 | 4.31 | 12.4 | 12.7 |
| 3.47 | 3.7 | 9.8 | 6.17 | 11.8 | 9.7 |
| 3.36 | 4.8 | 6.6 | 7.64 | 5.4 | 12.2 |
| 4.36 | 4.8 | 5 | 5.99 | 14.7 | 13.95 |
| 7.2 | | 0.9 | 1.5 | 13.3 | 21.05 |
| 7.18 | | 5 | 0.6 | 14 | 6.35 |
| 5.4 | | 12.15 | 1.05 | 12.6 | 12.2 |
| 8.05 | | 2.6 | 2.75 | 7.5 | 11 |
| 7.6 | | 7.25 | | | 12.2 |
| 4.1 | | 2 | | | 7.9 |
| | | 5.5 | | | 14 |
| | | 0.8 | | | 28.6 |
| | | | | | 8.8 |
| | | | | | 16.4 |

| |
|---|
| Male |
| Female |

S7B Cd11c Fth1 Macs/Monos Count

| Fth1fl/fl | | Fth1ΔCd11c | |
|---|---|---|---|
| 72hr | 96hr | 72hr | 96hr |
| 6.4 | 3.9 | 8.6 | 0.4 |
| 2.9 | 0 | 7.4 | 6.4 |
| 3.9 | 2.1 | 5 | 12.5 |
| 4.6 | 6.1 | 14 | 19.4 |
| | 16.4 | 11.6 | 0.1 |
| | 5.1 | 13 | 0.6 |
| | 6 | 11.8 | 10 |
| | 3.5 | 6.1 | 11.1 |
| | 0.5 | | 8.6 |

| |
|---|
| Male |
| Female |

S8A LysM Fth1 BALF MCP-3

| Fth1fl/fl | | | Fth1ΔLysM | | |
|---|---|---|---|---|---|
| RA | 72hr | 96hr | RA | 72hr | 96hr |
| 6.792 | 17.098 | 213.078 | 6.439 | 17.186 | 82.510 |
| 3.064 | 4.290 | 522.127 | 4.041 | 5.068 | 250.199 |
| 4.845 | 13.355 | 189.914 | 3.866 | 25.614 | 118.326 |
| | 165.632 | 274.413 | | 8.919 | 81.088 |
| | 4.041 | 383.340 | | 39.327 | 69.272 |
| | 29.302 | 519.353 | | 100.987 | 236.727 |
| | 17.516 | | | 35.267 | 269.476 |

| |
|---|
| Male |
| Female |

S8B LysM Fth1 BALF MCP-5

| | Fth1fl/fl | | | Fth1ΔLysM | |
|---|---|---|---|---|---|
| RA | 72hr | 96hr | RA | 72hr | 96hr |
| 5.147249 | 4.881927 | 33.7025 | 8.190147 | 8.321726 | 14.3293 |
| 5.103817 | 4.411203 | 35.99345 | 4.159774 | 8.437237 | 11.76666 |
| 4.411203 | 5.380947 | 33.7025 | 5.646792 | 11.53945 | 9.302879 |
| 4.411203 | 3.922405 | 20.38846 | 5.147249 | 6.655763 | 27.40816 |
| | 0 | 18.44957 | 12.47689 | 7.408099 | 28.7869 |
| | 4.904485 | 25.80742 | | 7.645045 | 15.3834 |
| | 4.159774 | 37.63198 | | | 24.20175 |
| | 4.411203 | 24.22291 | | | |
| | | 54.65251 | | | |
| | | 12.29945 | | | |

Male

Female

S9B BAL Live PMN Counts

| Fth1fl/fl | | Fth1ΔLysM | |
|---|---|---|---|
| RA | HO (72hr) | RA | HO (72hr) |
| 197 | 3305 | 104.6025 | 182 |
| 162 | 229 | 254 | 404 |
| 214 | 709 | 900 | 439 |
| 232 | 574 | 462 | 2030 |
| | 25 | 100 | 549 |
| | 187 | | 5772 |
| | 55 | | |

| |
|---|
| Male |
| Female |

S9C BAL Live PMN Percent

| Fth1fl/fl | | Fth1ΔLysM | |
|---|---|---|---|
| RA | HO (72hr) | RA | HO (72hr) |
| 81.1 | 98 | 100 | 86.9 |
| 96.4 | 92.7 | 96.9 | 87.3 |
| 93 | 97.7 | 85.8 | 96.7 |
| 84.7 | 97 | 97.1 | 97.2 |
| | 84.4 | 98.5 | 98.3 |
| | 94 | | 97.9 |
| | 83.8 | | |

| |
|---|
| Male |
| Female |

S10A Lip-1 Hyperoxia Survival

| Hours | DMSO | Lip-1 |
|---|---|---|
| 72 | 1 | |
| 72 | 1 | |
| 75 | | 1 |
| 76 | | 1 |
| 76 | | 1 |
| 95 | | 1 |
| 95 | | 1 |
| 95 | | 1 |
| 95 | 1 | |
| 95 | 1 | |
| 95 | 1 | |
| 95 | 1 | |
| 96 | | 1 |
| 97 | | 1 |
| 98 | 1 | |
| 98 | 1 | |
| 98 | 1 | |
| 98 | 1 | |
| 98 | | 1 |
| 98 | | 1 |

S10B Lip-1 Hyperoxia Weight Loss

| DMSO | Lip-1 |
|---|---|
| 9.2 | 9.9 |
| 15.2 | 18.5 |
| 10.4 | 14 |
| 15 | 16.9 |
| 7.5 | 14.6 |
| 18.7 | 12.5 |
| 16.5 | 8.6 |
| 14.6 | 11.2 |
| 16.9 | 13.3 |
| 13 | 10.3 |

| |
|---|
| Male |
| Female |

S10C Fer-1 Hyperoxia Survival

| Hours | DMSO | Fer-1 |
| --- | --- | --- |
| 75 | 1 | |
| 92 | | 1 |
| 96 | 1 | |
| 96 | | 1 |
| | | |
| 90 | 1 | |
| 90 | | 1 |
| 92 | 1 | |
| 92 | | 1 |
| 92 | | 1 |
| 96 | | 1 |

S10D Fer-1 Hyperoxia Weight Loss

| DMSO | Fer-1 |
|---|---|
| 13.1 | 20.1 |
| 18.8 | 18.6 |
| 19.7 | 19.4 |
| | 22.6 |

S11A LysM Fth1 Serum Hepcidin

| Fth1fl/fl | | | Fth1ΔLysM | | |
|---|---|---|---|---|---|
| RA | 72hr | 96hr | RA | 72hr | 96hr |
| 385.7531 | 797.4744 | 956.2599 | 427.8036 | 800.6884 | 1234.514 |
| 264.7334 | 520.0773 | 953.3684 | 84.72966 | 1238.329 | 1108.665 |
| 151.4275 | 1129.977 | | 201.9047 | 903.4786 | 1272.721 |
| 193.7071 | 777.3098 | | 326.2196 | 771.3928 | 1450.201 |
| | | | | 958.3362 | |
| | | | | 995.737 | |

S11B LysM Fth1 BALF Hepcidin

| Fth1fl/fl | | Fth1ΔLysM | |
|---|---|---|---|
| RA | 72hr | RA | 72hr |
| 6.541112 | 21.96285 | 2.426248 | 7.172421 |
| 14.49359 | 16.26417 | 4.200154 | 64.47726 |
| 10.3493 | 8.311764 | 13.48032 | 37.71338 |
| | 11.62757 | | 13.49858 |

S12A DFP FLAM Total Iron

| Ctrl | DFP |
|---|---|
| 25.36251 | 17.691 |
| 29.60428 | 15.51407 |
| 6.033274 | 2.44952 |
| 4.842563 | 2.3781 |

S12B DFP Hgb

| CTRL | DFP |
|---|---|
| 16.1 | 17.1 |
| 14.7 | 16.5 |
| 15.3 | 18.8 |
| 15.5 | 16.3 |
| 15.2 | 14.2 |
| 15.5 | |

S13A Cd11c x Ncoa4 AM Ncoa4 qPCR

| *Ncoa4 $^{fl/fl}$* | *Ncoa4 $^{\Delta Cd11c}$* |
|---|---|
| 1.0015 | 0.0102118 |
| 1.0012 | 0.0970563 |
| 1.015582 | 0.0227572 |
| 1.161 | 0.0118964 |
| 0.862 | 0.0749442 |
| 1.042691 | 0.1085198 |
| 0.961647 | 0.1899425 |
| 0.785806 | |
| 1.274478 | |
| 1.003293 | |

S13B Cd11c x Ncoa4 AM Fth1 qPCR

| *Ncoa4*$^{fl/fl}$ | *Ncoa4*$^{\Delta Cd11c}$ |
|---|---|
| 1.002328 | 1.273919 |
| 1.0045 | 0.855835 |
| 1.0008 | 1.624867 |
| 1.389364 | 1.477787 |
| 0.722142 | 1.145886 |
| 1.054599 | |
| 0.954681 | |
| 1.000076 | |

S13C Cd11c x Ncoa4 AM Tfrc qPCR

| *Ncoa4* $^{fl/fl}$ | *Ncoa4* $^{\Delta Cd11c}$ |
|---|---|
| 1.454729 | 1.541255 |
| 0.690889 | 1.547736 |
| 1.000068 | 1.008484 |
| 1.192997 | 2.877116 |
| 0.847933 | 3.823185 |
| 1.006169 | 3.088464 |
| 0.8999 | 2.482303 |
| 1.137043 | 3.993729 |

S13D Cd11c Ncoa4 AM Total Iron

| *Ncoa4*$^{fl/fl}$ | *Ncoa4*$^{DCd11c}$ |
|---|---|
| 1.179 | 0.951 |
| 2.399 | 1.703 |
| 1.266 | 0.918 |
| 1.835 | 2.989 |
| 1.322 | 1.599 |
| 1.242 | 1.571 |
| 1.130 | 1.423 |
| 1.469 | 1.507 |

S13E Cd11c Ncoa4 BALF Cell Count

| Ncoa4fl/fl | | Ncoa4ΔCd11c | |
|---|---|---|---|
| RA | HO (96hr) | RA | HO (96hr) |
| 7.3 | 7.8 | 4.55 | 3.7 |
| 2.3 | 6.85 | 4.7 | 1.75 |
| 4.85 | 3.5 | 4.05 | 1.8 |
| | 4 | | 1.75 |
| | 0.95 | | 2.2 |
| | 1.5 | | 1 |

| |
|---|
| Male |
| Female |

S13F Cd11c Ncoa4 BALF Macs/Monos

| Ncoa4fl/fl | | Ncoa4ΔCd11c | |
|---|---|---|---|
| RA | HO (96hr) | RA | HO (96hr) |
| 7.2 | 7.5 | 4.4 | 3.6 |
| 2.1 | 6.5 | 4.6 | 1.4 |
| 4.7 | 3.3 | 3.9 | 0.8 |
| | 3.6 | | 1.2 |
| | 0.3 | | 0.9 |

| |
|---|
| Male |
| Female |

S13G Cd11c Ncoa4 BALF Iron

| Ncoa4fl/fl | | Ncoa4ΔCd11c | |
|---|---|---|---|
| RA | HO (96hr) | RA | HO (96hr) |
| 39.5547 | 89.4771 | 31.3888 | 77.0995 |
| 83.5247 | 32.5865 | 26.1702 | 61.5896 |
| 22.749 | 61.4669 | 18.0078 | 119.94 |
| | 106.987 | | 102.814 |
| | 136.66 | | 142.231 |
| | 93.0699 | | 154.894 |
| | 70.196 | | 98.1927 |

| |
|---|
| Male |
| Female |

S14A LysM Fth1 Hyperoxia WBC

| Fth1fl/fl | | | Fth1ΔLysM | | |
|---|---|---|---|---|---|
| RA | 72hr | 96hr | RA | 72hr | 96hr |
| 11.3 | 3.62 | 2.26 | 9.34 | 4.61 | 6.95 |
| 5.02 | 3.61 | 2.44 | 8.08 | 6.66 | 3.24 |
| 6.62 | 3.71 | 3.95 | 8.8 | 5.55 | 4.78 |
| 7.77 | 2.38 | 4.1 | 8.46 | 2.14 | 3.56 |
| 11.3 | 1.86 | 7.78 | 6.22 | 3.67 | 5.75 |
| | 4.15 | 6.85 | 8.13 | 3.75 | 5.31 |
| | 3.31 | | 7.68 | 2.49 | 5.77 |
| | 3.22 | | | 3.13 | 3.88 |
| | | | | 2.64 | |
| | | | | 3.29 | |

Male

Female

S14B LysM Fth1 Hyperoxia Hgb

| Fth1fl/fl | | | Fth1ΔLysM | | |
|---|---|---|---|---|---|
| RA | 72hr | 96hr | RA | 72hr | 96hr |
| 15.5 | 18.4 | 20 | 17.2 | 18.6 | 18.8 |
| 17.5 | 17.3 | 18.8 | 16.6 | 18.2 | 18.2 |
| 14.7 | 18.4 | 19.4 | 15.8 | 17.9 | 18.2 |
| 15.4 | 19 | 16.5 | 15.7 | 18.3 | 14.8 |
| 16 | 18.5 | 16.9 | 14.1 | 18.9 | 17.9 |
| | 19.2 | 17.3 | 15.5 | 19.6 | 19.6 |
| | 18.9 | | 15.1 | 19 | 16.8 |
| | 17.9 | | | 17.6 | 17.7 |
| | | | | 20 | |
| | | | | 18.4 | |

Male

Female

S14D LysM Fth1 Plasma Cytokines (Z-Scores)

| | | | | | | | | | | | | | | | | | | | | | |
|---|---|---|---|---|---|---|---|---|---|---|---|---|---|---|---|---|---|---|---|---|---|
| | | Fth1fl/fl Ra | | | | Fth1ΔLysM RA | | | | | | Fth1fl/fl Hyperoxia | | | | | | Fth1ΔLysM Hyperoxia | | | |
| CXCL10 | 0.20432 | 0.876173 | 0.860823 | 0.310589 | 1.211926 | 0.716006 | 2.023873 | 2.317952 | -0.94359 | -0.5556 | -0.47656 | -0.0444 | -0.93664 | -0.85836 | -0.83544 | -1.04569 | -0.63715 | -0.93893 | -0.60082 | -0.64847 | |
| CXCL9 | -0.09455 | 1.114237 | 0.215654 | -0.52895 | 0.598352 | 0.006041 | 0.207225 | 1.098502 | -1.16453 | -0.97122 | 0.317932 | -0.88355 | -0.94705 | 3.146303 | -0.26651 | -1.48598 | 0.08865 | 0.303321 | -0.08106 | -0.67281 | |
| IFNγ | 0.072433 | -0.47081 | -0.47081 | -0.47081 | 1.656906 | 2.426507 | -0.47081 | -0.47081 | -0.47081 | -0.47081 | -0.47081 | 0.072433 | -0.47081 | -0.47081 | -0.47081 | 2.833943 | -0.47081 | -0.47081 | -0.47081 | -0.47081 | |
| IL-6 | -0.81216 | -0.86052 | -0.85194 | -0.85194 | -0.76302 | -0.84882 | -0.84882 | -0.88548 | -0.80592 | -0.24121 | 2.030106 | -0.58908 | 1.023925 | -0.07819 | 1.001306 | 1.016125 | 2.32026 | 0.459216 | 0.686192 | -0.10003 | |
| IL-10 | 0.854118 | -0.58649 | -1.48461 | -2.0271 | 0.492459 | -0.22483 | 0.136828 | -0.94212 | 1.933068 | 0.492459 | 1.215777 | 0.854118 | 0.136828 | 1.215777 | -0.22483 | 0.136828 | 0.854118 | -0.58649 | -1.30378 | -0.94212 | |
| IL-12p40 | -0.50122 | 0.689475 | -0.7407 | -0.26841 | 0.450005 | 0.210534 | -0.96686 | 1.161764 | -0.96686 | -0.96686 | 0.210534 | 0.210534 | 1.161764 | -0.26841 | -0.26841 | -0.59991 | -0.59991 | -0.59991 | -0.59991 | 0.689475 | |
| IL-12p70 | 0.586168 | -0.00446 | -0.51268 | -0.51268 | 0.290852 | -0.29978 | -0.51268 | -0.51268 | -0.51268 | -0.00446 | -0.51268 | -0.51268 | 0.586168 | -0.51268 | -0.51268 | -0.96686 | -0.96686 | -0.96686 | 0.689475 | -0.51268 | |
| TNFα | 0.255858 | -0.59991 | -0.59991 | 1.3474 | 2.534998 | -0.59991 | 2.002325 | -0.59991 | -0.59991 | -0.59991 | -0.59991 | -0.59991 | 1.3474 | -0.59991 | -0.59991 | 0.910783 | -0.59991 | -0.59991 | -0.59991 | -0.59991 | |

S15B Human ARDS Plasma Ferritin Subunits by Proteomics

| FTH | FTL |
| --- | --- |
| 22.29 | 77.71 |
| 29.25 | 70.75 |
| 25.97 | 74.03 |
| 24.69 | 75.31 |
| 18.82 | 81.18 |
| 11.1 | 88.9 |
| 8.37 | 91.63 |
| 34.15 | 65.85 |
| 31.19 | 68.81 |
| 42.54 | 57.46 |
| 33.44 | 66.56 |
| 12.94 | 87.06 |

S15C Human ARDS Ferritin and Iron

| Ferritin | Total Iron |
|---|---|
| 1399 | 1489.013 |
| 137.5 | 6091.68 |
| 1713.2 | 958.591 |
| 162.5 | 801.016 |
| 31.2 | 1662.057 |
| 729.6 | 506.554 |
| 1071 | 1055.2 |
| 177.5 | 645.837 |
| 22.9 | 861.418 |
| 1269.4 | 1345.779 |
| 481.9 | 909.015 |
| 378.9 | 575.119 |
| 903.2 | 323.686 |
| 1081.8 | 485.185 |
| 171.5 | 265.895 |
| 508 | 683.927 |
| 1988.3 | 1959.404 |
| 1300 | 1555.795 |
| 558.9 | 13258.23 |
| 1559.8 | 1763.176 |
| 1086.4 | 1366.854 |
| 1335.4 | 626.121 |
| 3077.6 | 2169.575 |
| 834 | 1152.608 |
| 486.9 | 1391.664 |
| 297.5 | 909.441 |
| 504.7 | 1914.353 |
| 222.2 | 840.681 |
| 438.2 | 727.738 |
| 411.9 | 5531.076 |
| 2554 | 1657.826 |
| 1991.9 | 1651.315 |
| 1204.5 | 2446.015 |

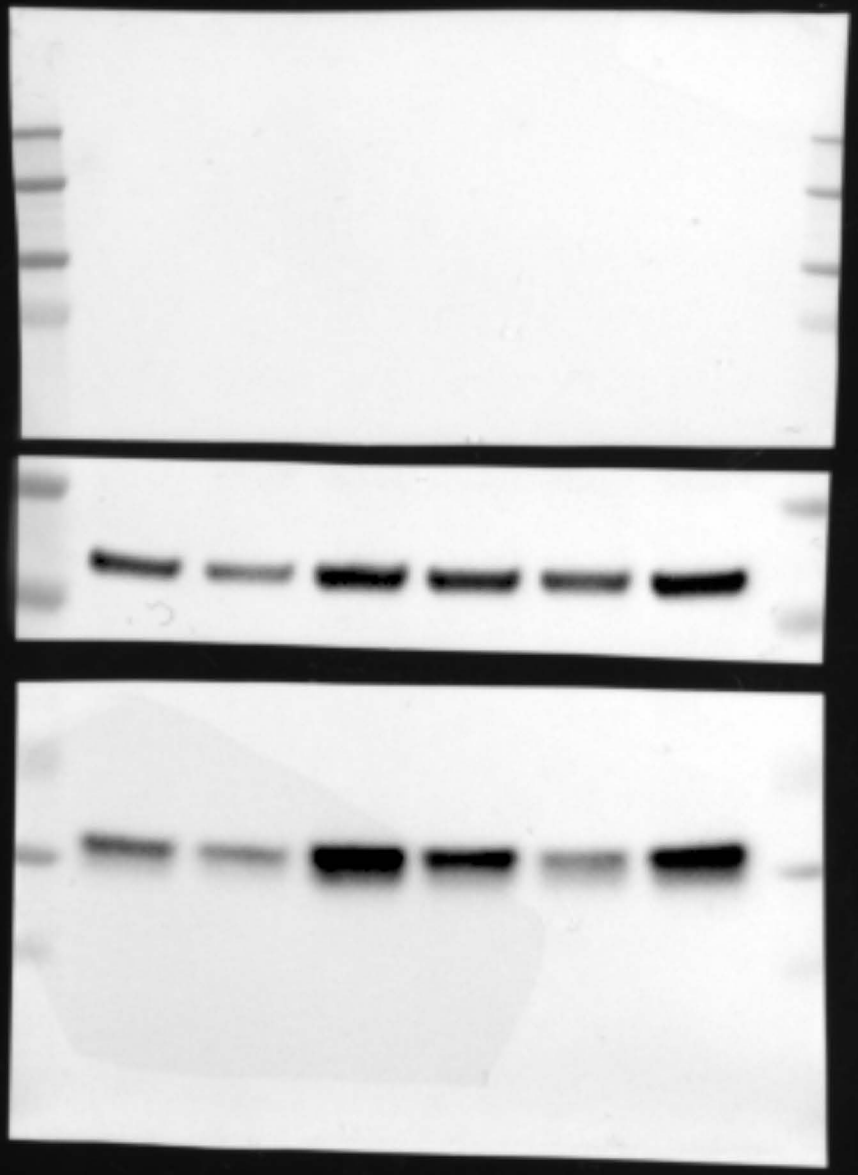

2M   BACTIN (middle) and
FTH1 (bottom)

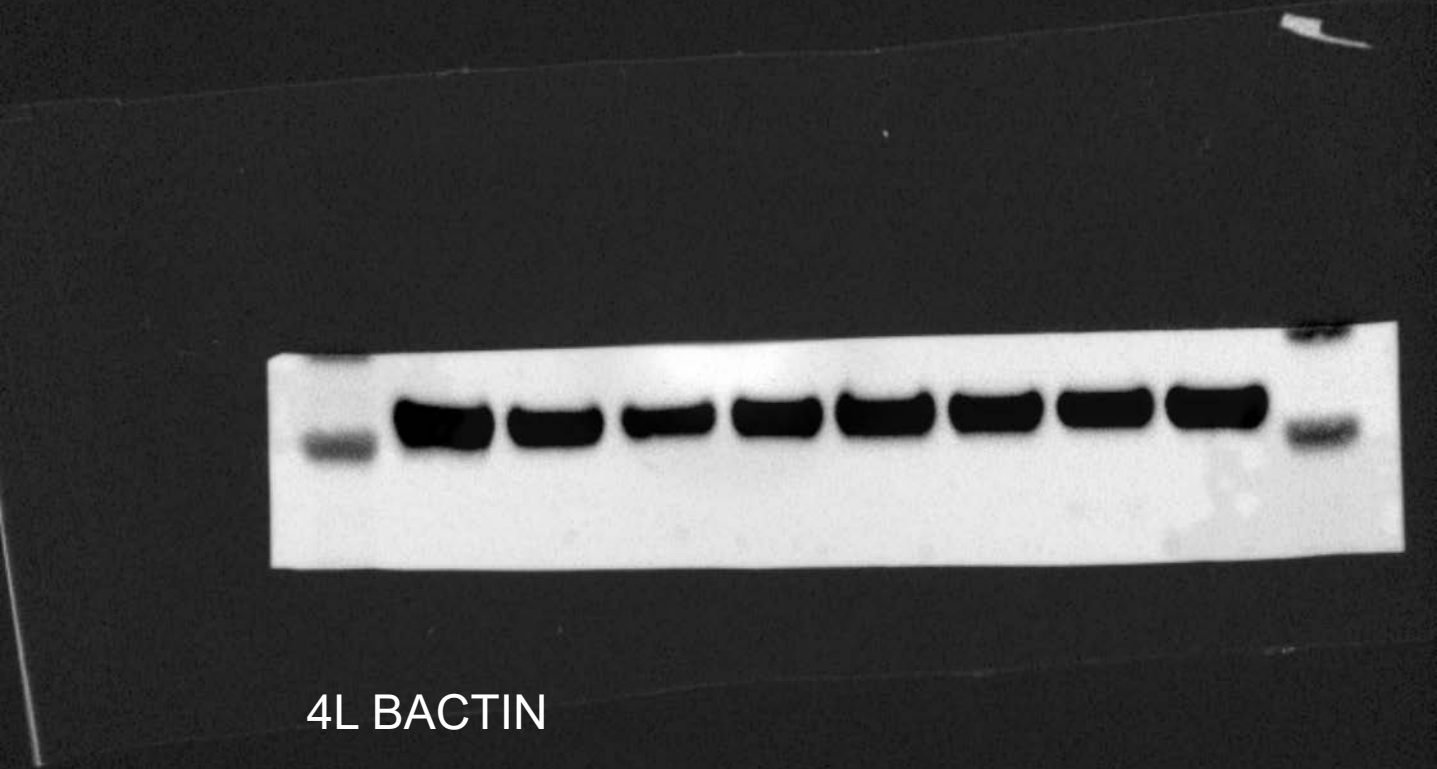

4L BACTIN

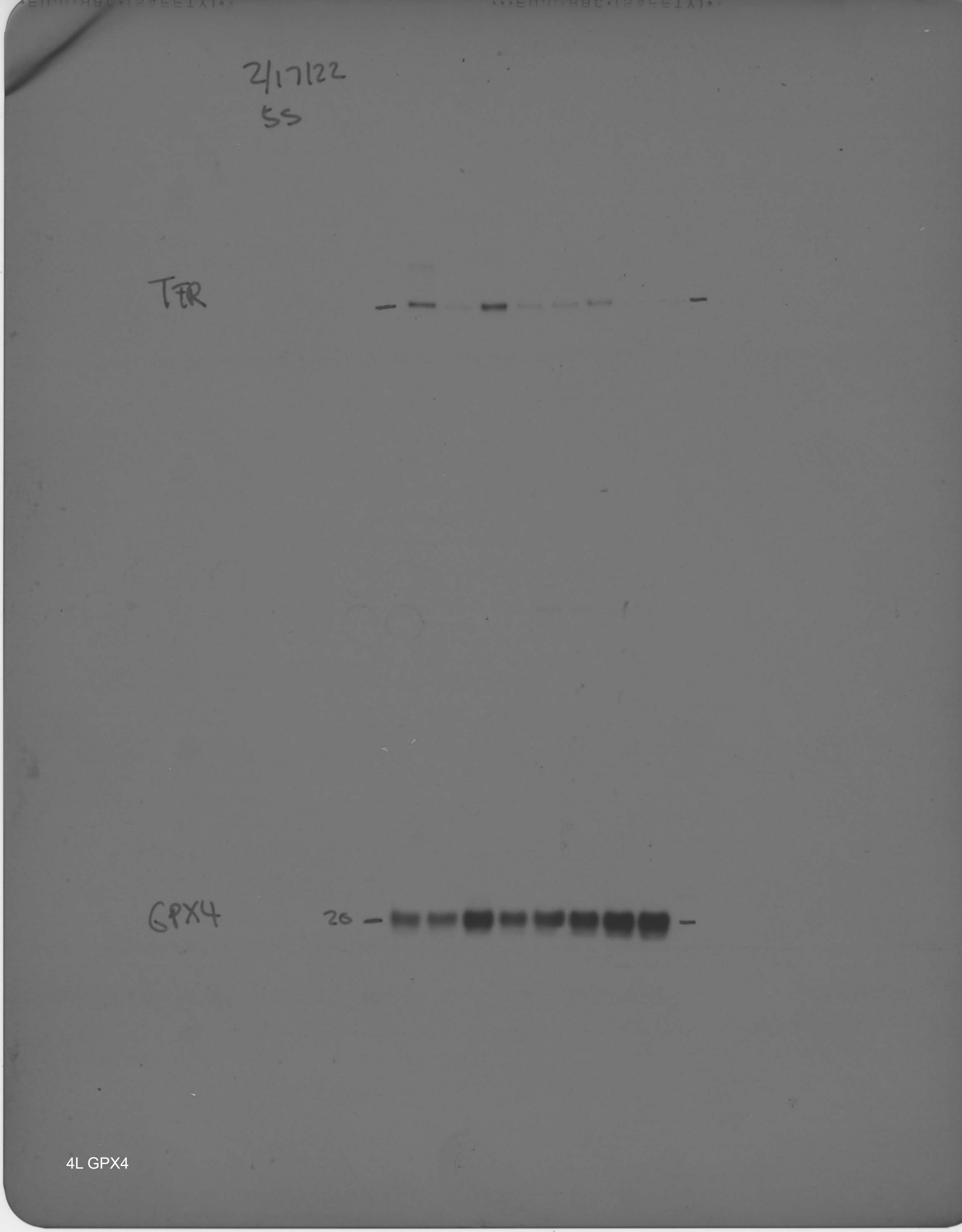
2/17/22
5S
TFR
GPX4
20
4L GPX4

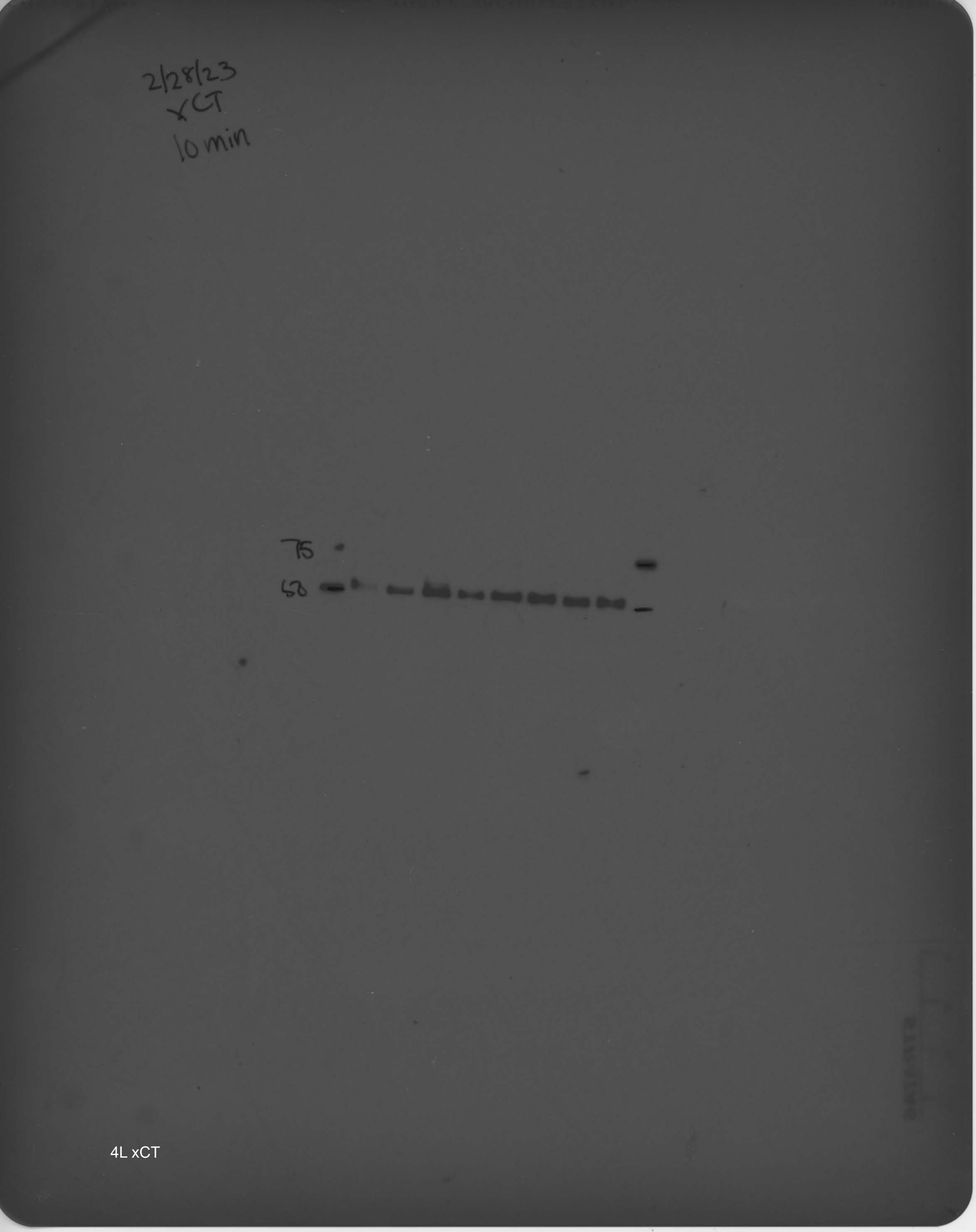
2/28/23
xCT
10 min
75
50
4L xCT

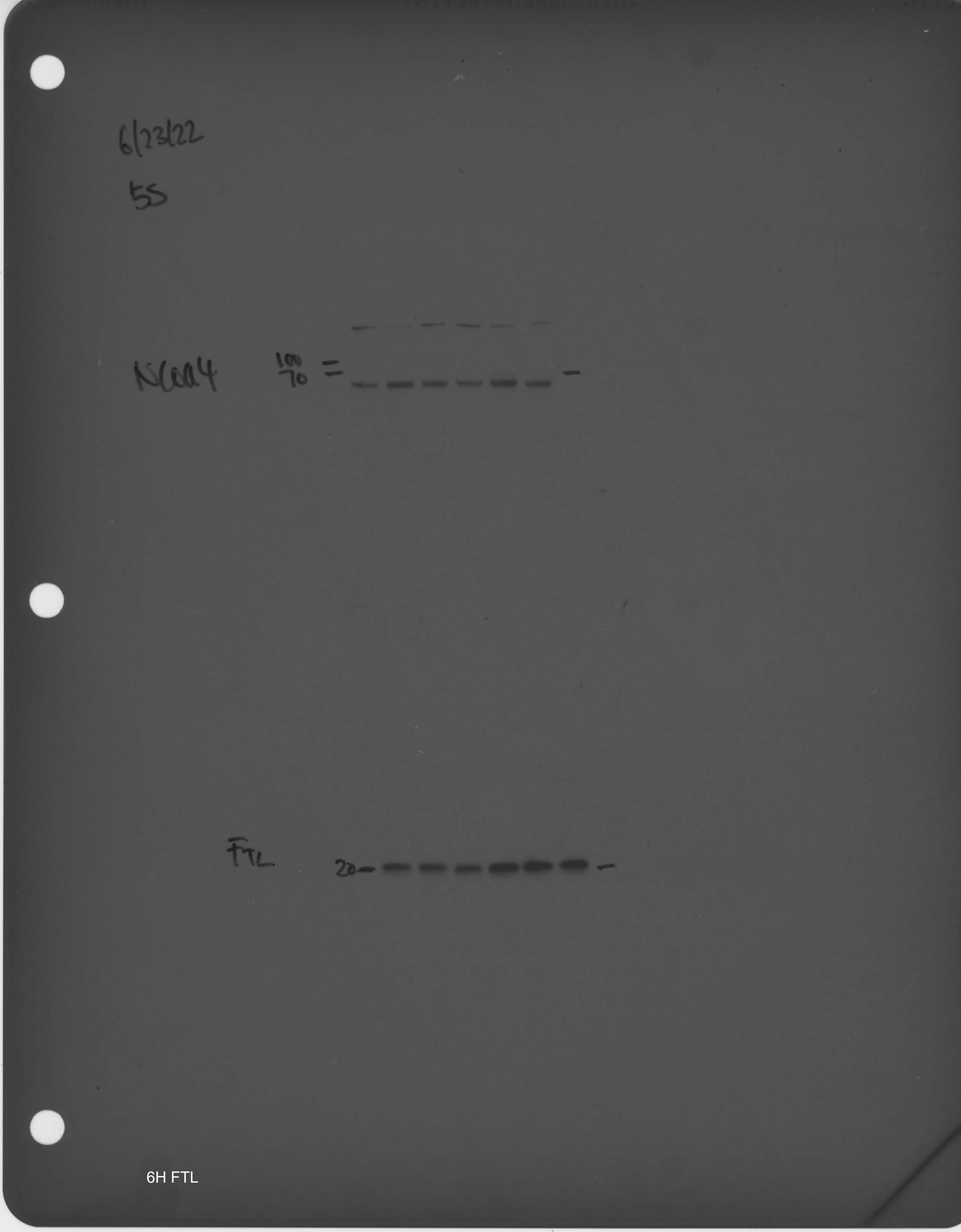


6H FTL

S5D BACTIN Lanes 1,
2, 5, 6

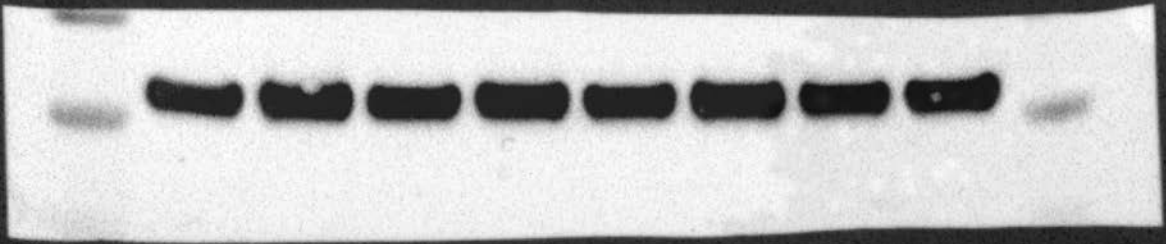

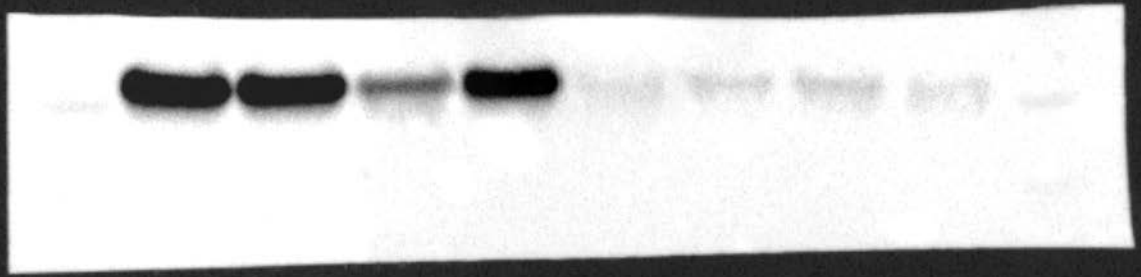

S5D FTH1 Lanes 1, 2,
5, 6

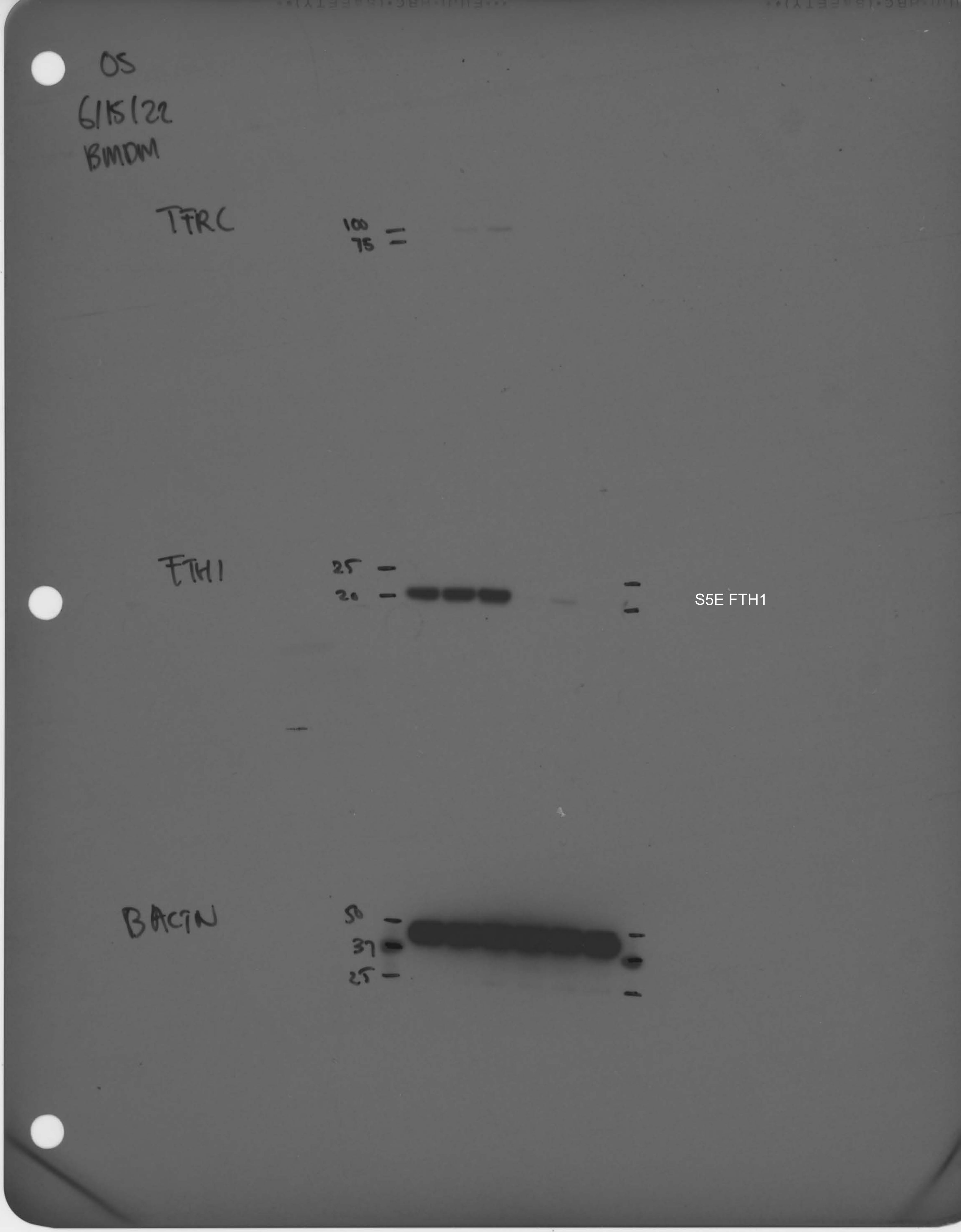
OS
6/15/22
BMDM
TFRC
100
75
FTH1
25
20
S5E FTH1
BACTIN
50
37
25

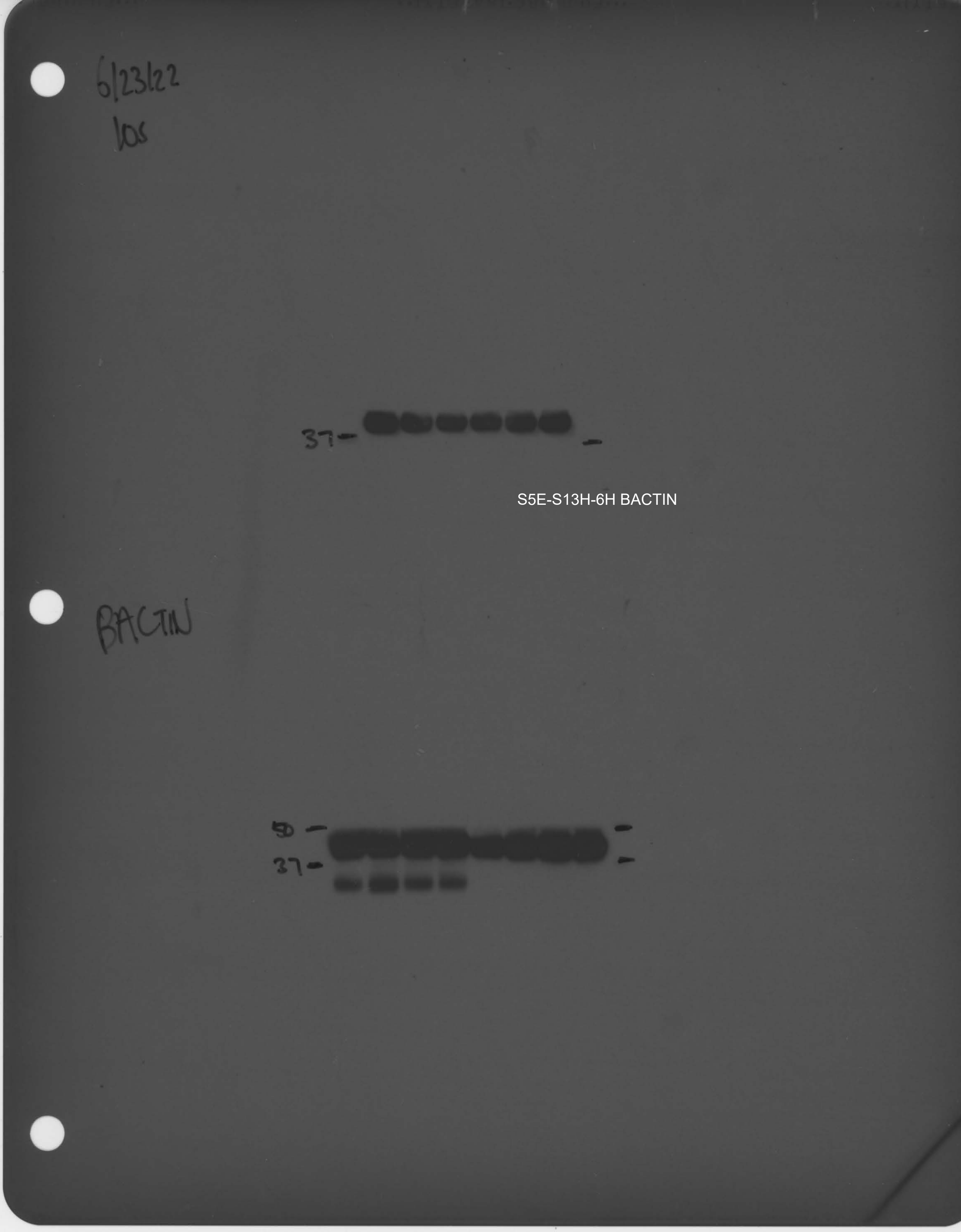
6/23/22
10s
37
S5E-S13H-6H BACTIN
BACTIN
50
37

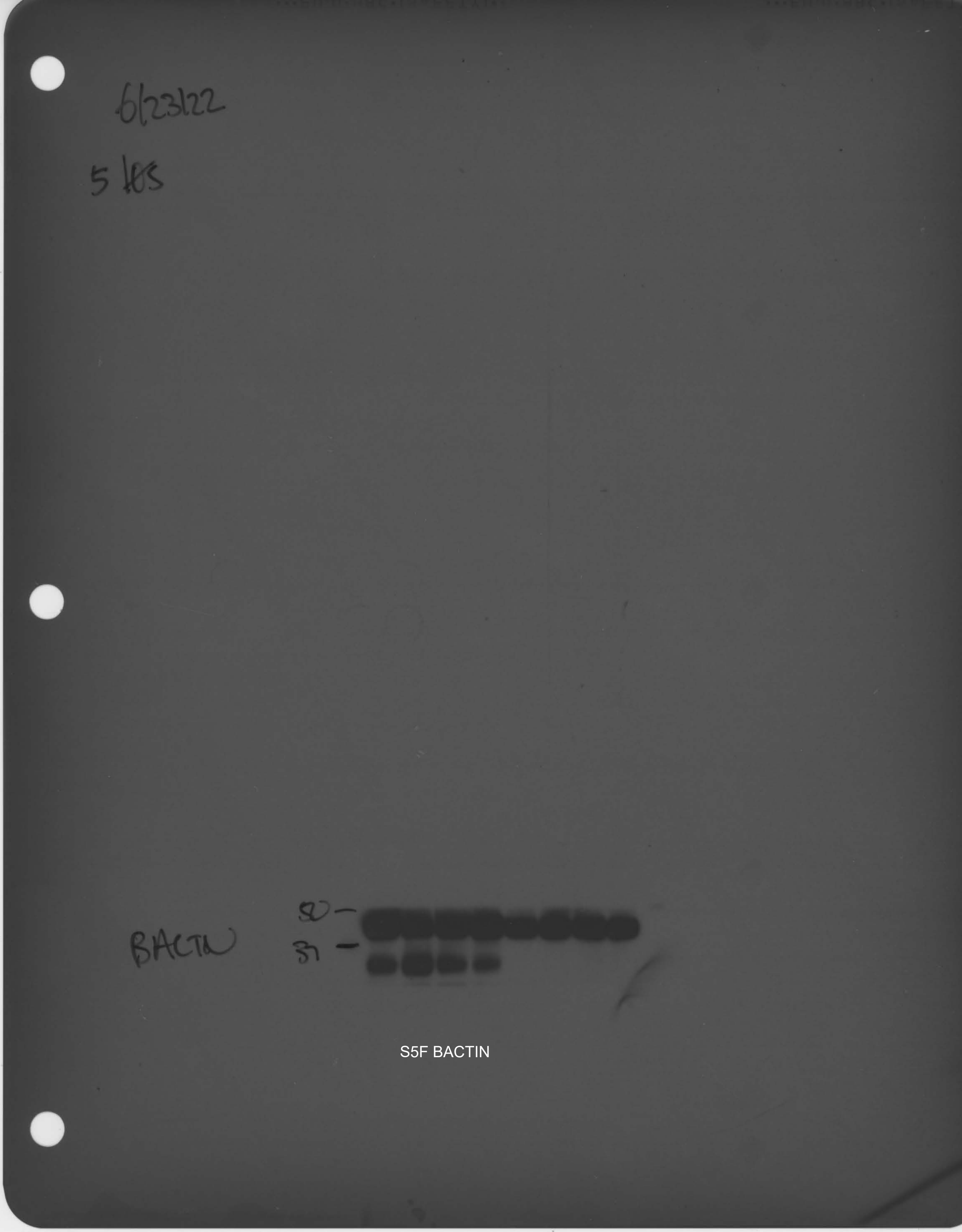
6/23/22
BACTIN
50
37
S5F BACTIN

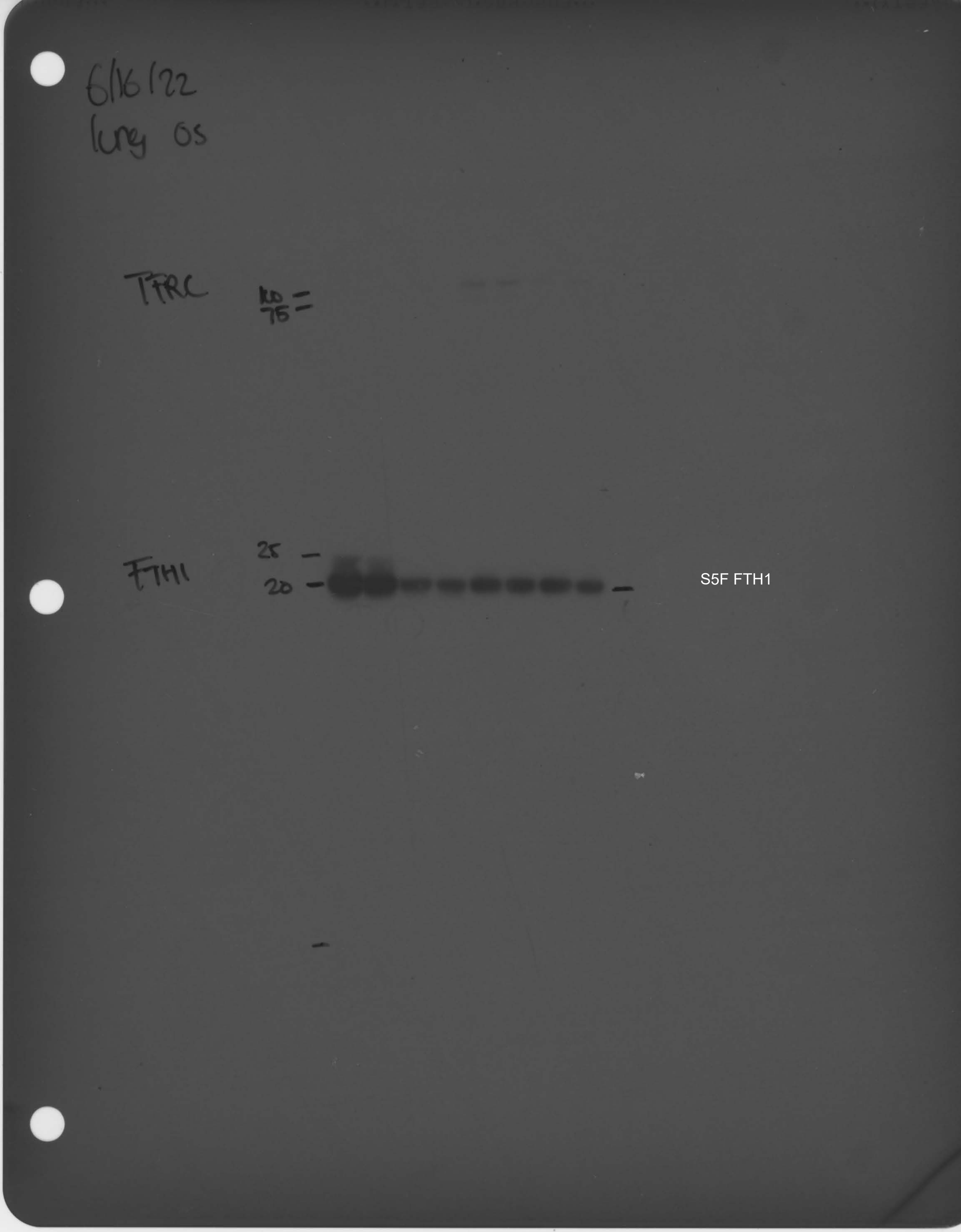
6/16/22
lung OS
TFRC
100
75
FTH1
25
20
S5F FTH1

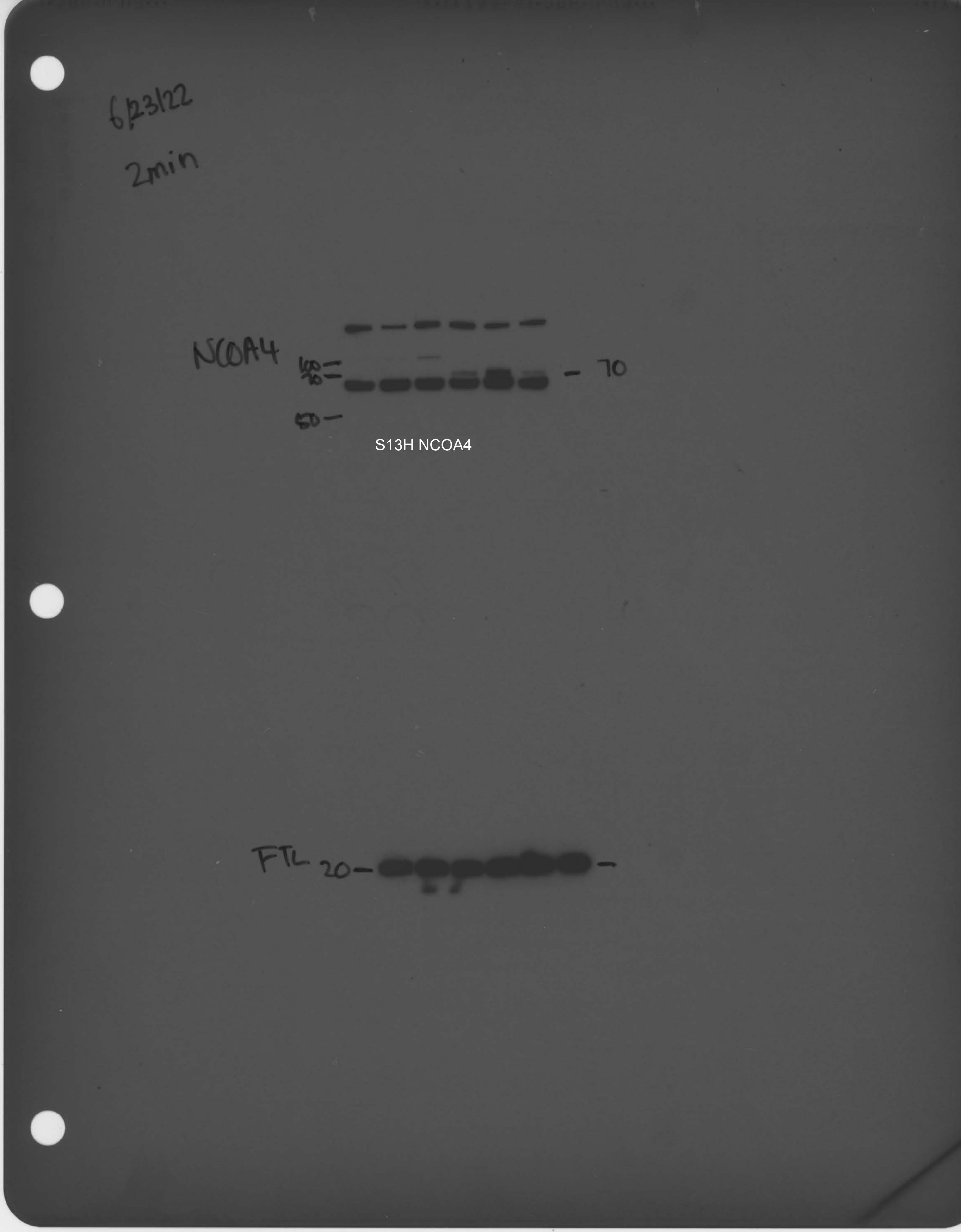
6/23/22
2min
NCOA4
70
50
S13H NCOA4
FTL
20

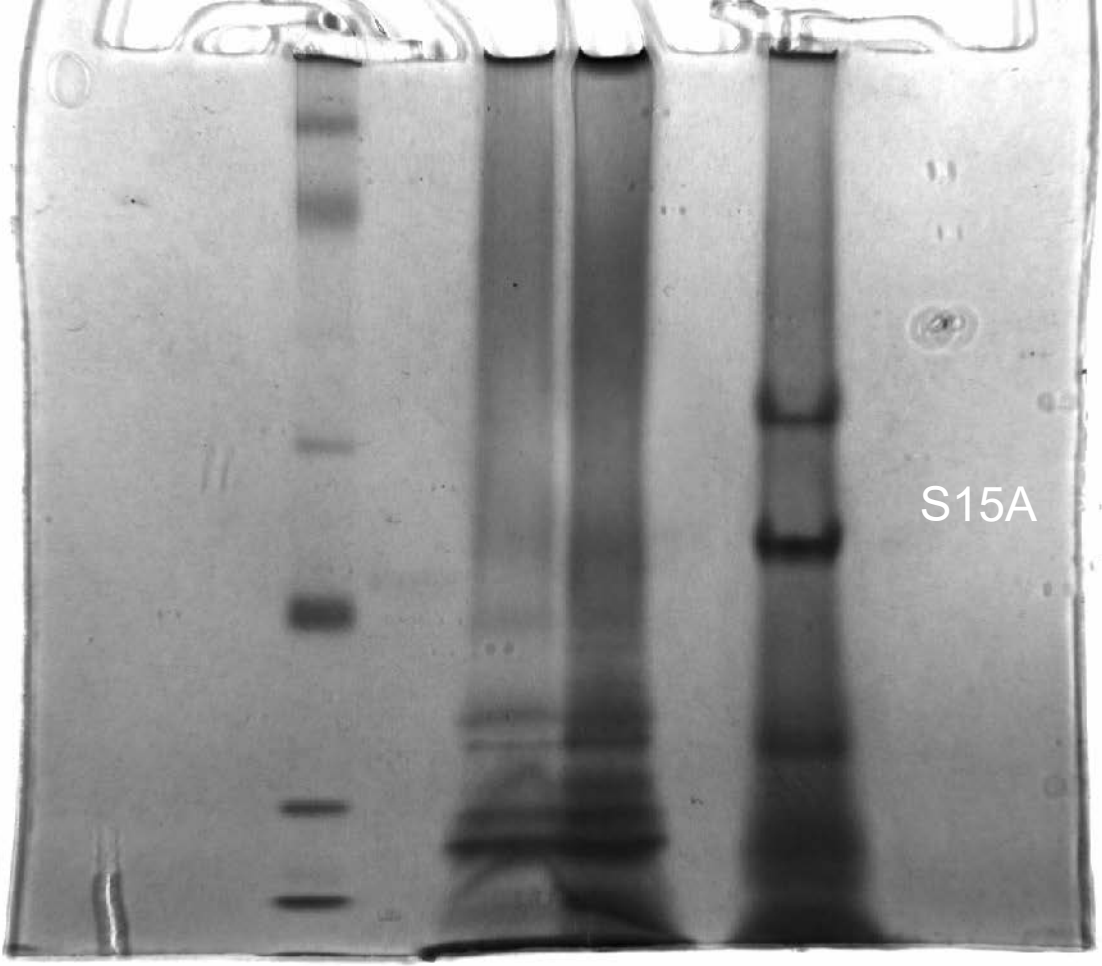
S15A
